# Modeling for COVID-19 college reopening decisions: Cornell, a case study

**DOI:** 10.1073/pnas.2112532119

**Published:** 2022-01-04

**Authors:** Peter I. Frazier, J. Massey Cashore, Ning Duan, Shane G. Henderson, Alyf Janmohamed, Brian Liu, David B. Shmoys, Jiayue Wan, Yujia Zhang

**Affiliations:** ^a^School of Operations Research & Information Engineering, Cornell University, Ithaca, NY 14850;; ^b^Center for Applied Mathematics, Cornell University, Ithaca, NY 14850

**Keywords:** epidemiological modeling, parameter uncertainty, asymptomatic screening

## Abstract

We consider epidemiological modeling for the design of COVID-19 interventions in university populations, which have seen significant outbreaks during the pandemic. A central challenge is sensitivity of predictions to input parameters coupled with uncertainty about these parameters. Nearly 2 y into the pandemic, parameter uncertainty remains because of changes in vaccination efficacy, viral variants, and mask mandates, and because universities’ unique characteristics hinder translation from the general population: a high fraction of young people, who have higher rates of asymptomatic infection and social contact, as well as an enhanced ability to implement behavioral and testing interventions. We describe an epidemiological model that formed the basis for Cornell University’s decision to reopen for in-person instruction in fall 2020 and supported the design of an asymptomatic screening program instituted concurrently to prevent viral spread. We demonstrate how the structure of these decisions allowed risk to be minimized despite parameter uncertainty leading to an inability to make accurate point estimates and how this generalizes to other university settings. We find that once-per-week asymptomatic screening of vaccinated undergraduate students provides substantial value against the Delta variant, even if all students are vaccinated, and that more targeted testing of the most social vaccinated students provides further value.

## Significance

Decisions surrounding how to safely reopen universities directly impact 7% of the US population (students, staff) and indirectly impact tens of millions more (families, communities). After witnessing large COVID-19 outbreaks among students from August 2020 to the present, universities want to provide safety while minimizing social and financial costs, despite uncertainty about vaccine hesitancy, vaccine efficacy, more transmissible variants with the potential for immune escape, and community prevalence. When the Delta variant is dominant, we find substantial risk reduction in moving student populations from mostly (75%) to fully (100%) vaccinated, in testing vaccinated students once per week even when all students are vaccinated, and in more frequent testing targeted to the most social groups of students.

When is it safe to offer in-person university instruction during the COVID-19 pandemic? What interventions, if any, provide the level of safety required? Colleges and universities across the globe faced this question in summer 2020 as they considered whether to offer in-person instruction. They continue to face this question today as they contemplate partially vaccinated student populations, waning immunity, booster shots, and the potential for new variants to emerge.

These questions are significant because outbreaks in university student populations have occurred regularly (1) and may harm the health of students and more-vulnerable employees and community members that interact with them (2). Even when vaccination protects the bulk of the population against the most severe health outcomes of severe acute respiratory syndrome coronavirus 2 (SARS-CoV-2) infection, widespread breakthrough infections would threaten the health of unvaccinated and immunocompromised individuals in their midst. At the same time, social distancing, masking, asymptomatic screening, the migration of in-person instruction to a virtual format, vaccine mandates, and other interventions that can be brought to bear against university outbreaks all incur social and financial costs (3, 4). Better understanding the protection offered by these interventions would support providing safety while minimizing these costs.

These questions remain difficult to answer because vaccination levels, SARS-CoV-2 variants, and other conditions continue to change and because experiences at the city, state, and national level do not easily generalize to university populations. Indeed, university populations are younger than the general population and thus have increased rates of contact (5) that may elevate virus transmission (2, 6). In addition, universities can implement interventions that would be substantially more difficult for the general population, such as mandatory vaccination and mandatory asymptomatic screening (7, 8).

Universities have responded to this central question in dramatically different ways. In the 2020–2021 academic year, many schools went fully online, while many others opened for in-person instruction with a modest set of interventions centered around symptomatic testing, contact tracing, and social distancing (9). Moreover, those schools that opened for in-person instruction pursued dramatically different testing strategies (10). Some tested only symptomatic students, others tested all students once on arrival, and others tested all students at least once per week. In the fall 2021 semester, schools differ in whether they mandate vaccines, their testing strategies, and masking policies (11).

This diversity in approach reflects, in part, a diversity of circumstance, such as proximity to, and interaction with, population centers, prevalence in those population centers, availability of housing to quarantine students, and the desires of the surrounding community (12). However, it also reflects substantial continued uncertainty about how policy translates into outcomes. Such uncertainty and diversity in approach among universities reflects the larger response to the pandemic, in which US states and national governments adopted dramatically different responses to the pandemic despite apparently similar circumstances.

Simulation-based epidemic models would seem to offer the power to resolve this uncertainty in support of high-quality decisions. They allow prediction, customized to the circumstances of a university, city, state, or nation. By varying the interventions in silico and observing predicted outcomes, one can hope to choose the best course of action. Unfortunately, epidemic models only approximate reality (13). Ever-present uncertainty in model input parameters coupled with the potential for exponential growth significantly limit accuracy. Small differences in behavioral and biological parameters can cause huge differences in predicted case counts. As a consequence, epidemic models have been maligned for producing inaccurate point estimates (13, 14).

This article demonstrates that simulation models can support effective selection of COVID-19 interventions even when they are unable to provide accurate point estimates of epidemic outcomes. We demonstrate this through a case study of how simulation models supported the design of COVID-19 interventions that were subsequently implemented at Cornell University. We also present a modeling framework that can support decisions at other universities. (The use of epidemic models in the presence of significant parameter uncertainty is also discussed in, for example, ref. 15. In such settings, clear communication of uncertainties is key; see, for example, ref. 16).)

In close communication with Cornell University’s administration, we conducted a simulation-based analysis in summer 2020 using a compartmental Susceptible, Exposed, Infectious, and Recovered model with multiple subpopulations; see refs. 17–19 for closely related models. Our work was the basis for the decision to reopen Cornell’s Ithaca campus for residential instruction in fall 2020 (20) and was used to design an asymptomatic screening program that was and remains a critical part of Cornell’s strategy.

Based on these modeling recommendations, all students were invited to return to Cornell’s Ithaca campus for residential instruction during the 2020–2021 school year under an asymptomatic screening program, and 75% of students returned (21). The surveillance program used pooled PCR testing with the testing frequencies obtained through our modeling. The surveillance program used less-sensitive but more-comfortable anterior nares (AN) sampling over nasopharyngeal (NP) sampling, because modeling suggested that the benefits of comfort to test compliance outweighed a potential loss in sensitivity. Asymptomatic surveillance was enabled at Cornell through a major effort to support large-scale sample collection and develop a new COVID-19 testing laboratory based on diagnostic expertise in Cornell’s College of Veterinary Medicine, and through a unique partnership with a local health care provider. Based on recommendations from our simulation modeling approach, this strategy was updated for the spring 2021 semester to test varsity athletes and students in Greek-life organizations more frequently (contact tracing data showed them to have more social contact than other individuals) and again in fall 2021 to adjust for the Delta variant, changes in social distancing policies, and the protection offered by vaccination. Over the course of the 2020–2021 academic year, there were fewer than 1,044 infections among students and employees, fewer than many schools with similar student populations offering only virtual instruction (1, 22).

Our modeling approach hinges on delineating those simulation model input parameters yielding epidemics that can be successfully controlled versus those that cannot. If the set of plausible input parameters are contained within the set of safe parameters, then we can be highly confident, although never certain, that the epidemic can be controlled. At Cornell in summer 2020, we demonstrated this to the university administration for a suite of interventions available with in-person instruction: frequent asymptomatic screening, testing students on arrival to campus, contact tracing, social distancing on campus, limits on student and employee travel, masking requirements, and a behavioral compact curtailing student social gatherings. It was also possible that we would have found that plausible ranges of the input parameters overlapped the portion of parameter space where epidemics would grow out of control, in which case we would not have been able to recommend reopening.

We found that access to regular asymptomatic screening (7, 23), with an ability to increase testing frequency if needed, was critical. Indeed, those few universities employing a similar asymptomatic screening approach succeeded, by and large, in controlling campus outbreaks (24–27). See also refs. 28–33 for explorations of the interaction of pooled testing and asymptomatic surveillance for controlling epidemics.

We also found it was critical to analyze epidemic growth if in-person instruction were not offered, to quantify the relative merits of the alternative to in-person instruction. Survey results (20, 34) suggested that a significant number of students would return to the Ithaca area even if in-person instruction were not offered. Without the benefits of the legal framework offered by in-person instruction, frequent asymptomatic screening would have been difficult to mandate for this population. Moreover, our analysis suggested that many of those parameter settings in which asymptomatic screening would not ensure safe in-person instruction would also be ones in which a significant outbreak would occur in the local student population under virtual instruction. This resulted in the decision to reopen Cornell’s Ithaca campus with a fully residential semester in fall 2020 (20).

We additionally measure key parameters of a university population needed for understanding the dynamics of epidemic spread, including university subpopulations’ intergroup and intragroup rates of viral transmission and how it has changed over time with vaccination, the Delta variant, and relaxation in social distancing. We find that a small group of students has significantly more intergroup viral transmission than other groups and plays an important role in determining the risk of an outbreak. We find that targeting interventions to this group provides substantial protection against outbreaks. Unlike students, we find that employees have very little transmission at work and are well separated from students, with extremely little transmission across the two groups. This has implications for understanding the risk to older and more vulnerable individuals from student infections.

When considering a range of interventions against the Delta variant, we find that achieving high levels of vaccination provides significant protection, but that, even in a 100% vaccinated student population, there is significant potential for breakthrough outbreaks in the absence of asymptomatic screening and social distancing. This is consistent with findings from other modeling studies (19). While once per week asymptomatic screening of vaccinated students might be sufficient in many situations, we find that testing vaccinated student groups with high rates of social contact twice per week substantially reduces risk even when the entire population is vaccinated. We also find that moving from 75% vaccination to full vaccination provides substantial additional protection.

To summarize, the key contributions of this paper are 1) providing a simulation framework for supporting the design of COVID-19 interventions despite parameter uncertainty; 2) demonstrating this framework through its implementation at Cornell University; 3) measuring key parameters of the dynamics of the spread of SARS-CoV-2 in university populations and the effectiveness of interventions; and 4) providing a framework for making decisions moving forward, including the design of asymptomatic screening strategies in the presence of partial vaccination and the Delta variant.

Our work adds to the broader literature using epidemic modeling in the context of universities. See, for example, ref. 35 for a perspective on the challenges of reopening as informed by a variety of epidemic models, refs. 36 and 37 for the use of agent-based modeling to evaluate mitigation strategies to enable safe in-person instruction, ref. 38 for probabilistic modeling of strategies to suppress virus spread in dorms and classrooms, and ref. 39 for a study of interventions for generic small residential campuses.

## Results

### Model Structure and Fit.

We focus on Cornell’s main campus, located in Ithaca, NY (40). Ithaca is located in Tompkins County with a population of ∼102,000. Approximately 12,000 undergraduates (of which 35% live on campus), 8,500 graduate students (of which 6,200 were local in Ithaca during the 2020–2021 academic year due to the pandemic), and 10,000 employees study or work on the Ithaca campus. Ithaca is 4 h to 5 h by car from major city centers such as New York, Boston, Philadelphia, and Toronto.

**Fig. 1.**
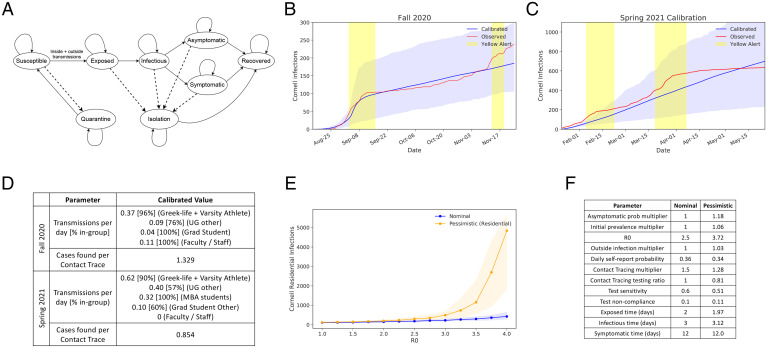
(*A*) The dynamics of our compartmental simulation across compartment categories (ellipses). Population counts are maintained for each compartment on each day, and compartments comprise a category, for example, “susceptible,” a demographic group, for example, undergraduates in high-density housing, and the elapsed time in that compartment category. Solid lines represent virus transmission, disease progression, and the end of quarantine/isolation. Dashed lines represent the effect of testing, self-reported symptoms, and contact tracing, which puts individuals testing positive into isolation and their contacts into quarantine. (*B* and *C*) Average cumulative case trajectories (and their 10th to 90th percentiles) for all Cornell cases under calibrated parameters and observed fall 2020 and spring 2021 cases, including presemester periods. Asymptomatic surveillance began on 3 September 2020. Students arrived to campus 16 August to 2 September and then again 21 January 2021 to 8 February before departing at the end of horizons shown on the respective graphs. The yellow regions indicate when the campus alert level was raised to yellow; it never went above this level. Increased testing and a lockdown were initiated on 26 March 2021 for 1 wk for MBA students. (*D*) Table summarizing the key fitted parameters from fall 2020 and spring 2021 calibration. (*E*) The number of infections (lines provide the median; shading indicates the 10th to 90th percentile range across simulation replications) as two key parameters vary while holding the others fixed, under nominal and pessimistic scenarios. (*F*) Parameter values for nominal and pessimistic scenarios for fall 2020.

In June 2020, we developed a compartmental simulation model to predict infections and hospitalizations for Cornell’s fall 2020 semester (Fig. 1*A*). The model contains compartments by subpopulation, stages of symptom development and infectivity, and quarantine/isolation status (*Methods A–C*). Since our model is stochastic, there is variance in case trajectories with fixed parameters. Infections in the Cornell population are roughly proportional to other outcomes of importance (*SI Appendix*, section 3.F), and therefore figures here focus on this outcome.

To demonstrate that the model can accurately capture reality, we use deidentified aggregated data obtained from surveillance testing in the 2020–2021 academic year (these data were unknown at the time recommendations were made) to fit model parameters and retrospectively model, with hindsight, the 2020–2021 academic year.

In this retrospective model fit, we first separated individuals into groups based on risks observed during the fall 2020 and spring 2021 semester. Employees constituted a single group. Students were separated into undergraduates who are in social Greek-life organizations or on varsity athletic teams, other undergraduates, and graduate/professional students. In the spring 2021 semester, graduate/professional students were additionally separated into those in the MBA program and other graduate/professional students.

Contact tracing data showed that transmission between students and nonstudent employees was extremely rare, so we set transmission parameters between employees and students to zero. This caused the model to treat them as two completely separated groups that do not infect each other.

We then used observed data to estimate other key model parameters: one governing the effectiveness of contact tracing for students and employees, the rate of transmission between the three student groups up to a proportionality constant, and the rate of infection from outside sources for students and employees.

After, one free parameter remained for employees (transmission rate) and one for students (a proportionality constant giving the transmission rate between each student group). We estimated these parameters, separately for employees and for students, by calibrating simulation results to observed data, varying each parameter to minimize the sum of squared differences between the mean of the model’s predictions and the observed cumulative infection count (*SI Appendix*, section 2).

Fig. 1*B* and *C* compares observed total (student + employee) cases to simulation trajectories from the calibrated model using the test frequencies implemented in practice. In the fall 2020 semester, undergraduates were tested twice per week, graduate students were tested once per week, and employees were tested at a frequency between twice per week and once every 2 wk specific to how often they were on campus. Over the spring 2021 semester, students affiliated with Greek-life organizations and varsity athletes were tested more frequently at three times per week. In late March 2021, a cluster of cases among students in the Masters in Business Administration (MBA) program led to elevating their test frequency to twice per week for the duration of the semester. As indicated in the figure, a color-coded campus alert level was used to warn students of elevated risk during four periods in this timeframe.

The average calibrated trajectory closely tracks observed cases for both fall 2020 and spring 2021. There is some deviation during both semesters when a single high-transmission individual or event led to a burst in the number of cases not captured by our model. Nevertheless, observed trajectories are contained within the 10th to 90th percentiles on nearly all days, indicated by the shaded regions in Fig. 1 *B* and *C*.

Fig. 1*D* summarizes key calibrated parameter values. Groups of students participating in Greek-life social organizations and varsity athletics are found to have a significantly higher rate of transmission than others. We find later that screening these groups more often significantly reduces risk. For most groups, estimated transmission in spring 2021 is higher than in fall 2020, consistent with colder weather and fatigue with social distancing restrictions driving more indoor gatherings and the Alpha variant becoming more prevalent in the United States. In spring 2021, the testing frequency for students in Greek life and varsity athletes was raised. This likely led to the reduction in the number of cases found per contact trace, as a contact trace was more likely to be initiated before a student had infected others. Lastly, the vast majority of cases spread within the same group. More detailed information on the spread between groups can be found in *SI Appendix*, section 2.

### Sensitivity of Model Predictions to Parameters.

While the model developed in the summer of 2020 had the capacity to model trajectories seen over the following academic year (2020–2021), key input parameters had not been measured accurately in college populations for SARS-CoV-2 in June 2020. Moreover, sensitivity analysis (*SI Appendix*, section 3.E) demonstrated that model predictions were sensitive to these unknown parameters. Fig. 1*E* shows the result of varying one of the uncertain parameters with the largest effect on outcomes while holding the others fixed at parameter values from the two scenarios in Fig. 1*F*. These scenarios were chosen as described in *Results C*.

To represent this uncertainty, we first identified ranges containing plausible values for each simulation parameter (*SI Appendix*, section 3.A) based on information available in June 2020.

**Fig. 2.**
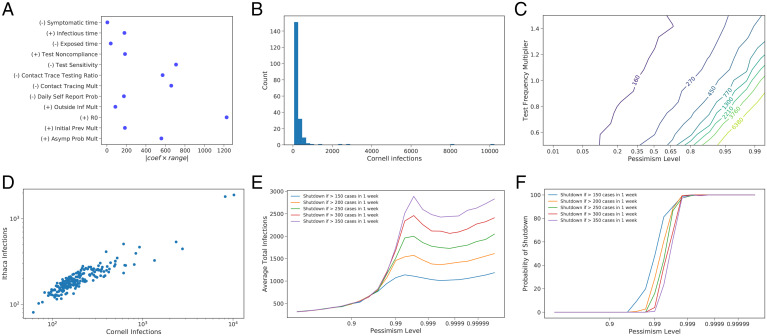
(*A*) The first-order effect of parameter uncertainty on predicted infections, using a linear model to estimate sensitivity of infections to each parameter and a range of plausible parameter values. Each dot shows the estimated effect of uncertainty on predicted infections (absolute value of the regression coefficient times the uncertainty range’s width) with the sign of the regression coefficient indicated in the label. The uncertainty of this estimated effect (derived from the regression coefficient’s 95% CI) is approximately ±60 for all parameters. Mult, multiplier; Prob, probability; Inf, infection; Prev, prevalence. (*B*) Histogram of median Cornell infections when parameters are sampled from the prior. (*C*) Contour plot showing the number of Cornell infections as test frequency and the level of pessimism changes. (*D*) Scatter plot showing the median number of Cornell and Ithaca infections for each of the 200 points sampled from the prior. (*E*) The average number of infections and (*F*) probability of shutdown under each of a set of shutdown policies that model the more nuanced decision-making process available to leaders in practice.

To understand the sensitivity of model predictions to uncertain parameters, we then evaluated infections assuming residential instruction at 2,000 parameter configurations chosen using a Latin Hypercube design (41) over the collective set of parameters defined by these plausible ranges (while also supporting a sensitivity analysis over four additional parameters used for predicting outcomes under virtual instruction; *SI Appendix*, section 3.E). We fit a linear model to quantify the first-order impact of each parameter on infections. We then multiply the estimated rate at which residential infections change as we vary a parameter (from this linear model) by the width of the range quantifying uncertainty for this parameter (Fig. 2*A* and *SI Appendix*, Table S22). To first order, this value is the change in predicted infections resulting from moving the parameter from the lower to the upper bound of its corresponding range. Parameters for which this value is largest are those that lead to the greatest uncertainty about infections, whether because our uncertainty about the parameter is large or because outcomes are sensitive to it.

The effect of uncertainty is substantial, with uncertainty about several individual parameters creating uncertainties of more than 500 infections relative to a baseline of ∼250 infections. The parameters that most drive uncertainty about infections are those that influence 1) transmission of the virus, especially *R*_0_ as well as two contact tracing parameters and a parameter governing the likelihood an infected individual develops symptoms, and 2) our ability to control virus transmission through testing (test sensitivity). These parameters are described in detail in *SI Appendix*, section 1.

### Coping with Parameter Uncertainty.

Uncertainty about parameters prevented accurate point estimates for the number of infections and presented the central challenge when deciding whether it would be safe to bring students back to campus. Against this challenge, we hypothesized that asymptomatic screening can prevent epidemic growth over some set of parameter settings. More frequent testing creates a larger set of safe parameter settings.

To understand whether a candidate value of 2×/wk asymptomatic screening would be enough to make residential instruction safe in the 2020–2021 academic year under plausible parameter values, we formed a Bayesian prior probability distribution over parameters consisting of independent normal distributions for each parameter. The (marginal) mean and variance of the prior over each parameter was chosen so that the resulting symmetric 95% Bayesian credible interval corresponded to the previously selected plausible ranges for each parameter (*Results B* and *SI Appendix*, section 3.A).

The use of a multivariate normal distribution as a prior reflects both modeling concerns and tractability concerns. We required a unimodal prior with ellipsoidal contours to permit the analysis that follows. The multivariate normal was the natural candidate, as discussed in more detail in *SI Appendix*, section 3.A.

We then drew random sets of parameters from this Bayesian prior, appropriately truncated to ensure feasible values for parameters known to be positive and/or bounded, and ran our simulation for each, forming a prior distribution over infections accounting for parameter uncertainty (Fig. 2*B*). Under most parameter settings, 2×/wk testing is sufficient to achieve substantial infection control, but large outbreaks occur under some parameter settings.

To better understand the impact of interventions like testing on robustness to parameter uncertainty, we developed a one-dimensional family of parameter configurations with varying levels of pessimism regarding the number of infections (*Methods D* and *SI Appendix*, section 3.A). This family of parameter configurations is indexed by a pessimism level between zero and one, with larger levels corresponding to parameter configurations with more infections. The parameter configuration at pessimism level *q* is the most likely configuration under the prior for which median infections is equal to the *q* quantile of infections under the prior, assuming that infections for a given parameter configuration are given by the previously fitted linear model.

Within this family, we name two for more frequent use, given in Fig. 1*F*: the nominal and pessimistic scenarios, corresponding to pessimism levels 0.5 and 0.99, respectively. We indicate the latter in plots as “pessimistic (residential)” to distinguish it from a scenario that is pessimistic about a different outcome defined in *Results D*. (The nominal scenario also corresponds to setting each parameter to the middle of its range, that is, to the mean of the prior.) Relative to the nominal scenario, the pessimistic scenario significantly increases the asymptomatic ratio and transmission rate while decreasing test sensitivity and contact tracing effectiveness. These parameters also have the largest absolute normalized effect on infections (Fig. 2*A*). This is visualized in *SI Appendix*, Fig. S15.

To understand the impact of the assumption of linearity used when creating these scenarios, we compared the median number of infections under *q* pessimistic scenarios against the *q* quantile of median infections, drawing parameters from the prior (*SI Appendix*, section 3.B), for varying *q*. We found strong agreement ( *SI Appendix*, Fig. S16), suggesting that the linearity assumption had little impact on the level of pessimism inherent in our scenarios.

**Fig. 3.**
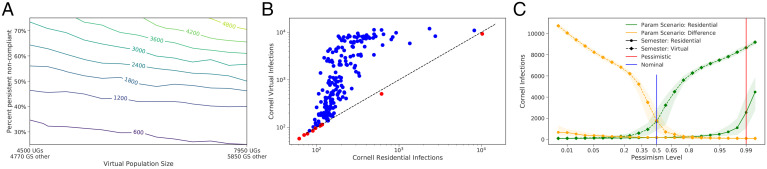
(*A*) The number of infections in the Cornell community under virtual instruction under the nominal scenario, varying the fraction of off-campus students engaged in virtual instruction that do not use the offered testing (“persistent noncompliance”) and the total number of off-campus students. If off-campus students’ willingness to comply with 2×/wk optional testing is not sufficiently high, a large number of infections result. (*B*) Under 200 parameter configurations drawn from the prior, the number of infections under virtual and residential instruction. Infections are smaller under residential instruction than under virtual instruction in most parameter configurations (blue dots), and when they are not (red dots) they are not substantially larger. (*C*) Number of infections under two pessimistic configurations (maximizing residential infections and residential - virtual infections) and two types of semesters (virtual and residential) for different levels of pessimism. Param, parameter.

We then plotted simulated infections using 2×/wk testing at parameter configurations across a range of pessimism levels (Fig. 2*C* at test frequency multiplier = 1.0; Fig. 3*C*). This level of testing is sufficient to keep the number of infections below 1,000 in all but the most pessimistic parameter configurations. Still, there are some parameter configurations where more than 1,000 infections arise, and infections grow rapidly as the parameter configuration grows more pessimistic. Additional testing can mitigate this risk (Fig. 2*D*). When the test frequency is high enough, predicted infections remain low even at pessimism levels as high as 0.99.

Plots of infections versus test frequency and simulation parameters were distributed in public reports (42) and at Cornell Faculty Senate meetings (43). These reports also included nominal and pessimistic scenarios (*SI Appendix*, section 3.C) similar to the ones detailed here, although highlighting more concerning outcomes. They were a central component of deliberation at Cornell on whether testing could allow a safe residential reopening. They resulted in the decision by Cornell leadership that, if the campus were to open for residential instruction, we would use a test frequency that was as large as could be provided reliably, to maximize the range of parameter settings with effective infection control.

While we focus here on a single outcome, infections in the campus population, other outcomes are important: infections in the surrounding community created by clusters in the campus population, and hospitalization and deaths. As shown in Fig. 2*E* and *SI Appendix*, section 3.F, these outcomes tend to move together when varying parameters and the overall frequency of testing. Thus, for the purposes of understanding the overall level of risk and deciding whether to reopen, considering only on-campus infections tends to produce the same decisions as would more holistic consideration of on-campus and community outcomes examining infections, hospitalizations, and deaths.

In addition to testing, which was instituted at the start of the semester, a second measure combating parameter uncertainty is recourse: the opportunity to change interventions based on data as it is observed, ranging from, for example, enforcement of behavioral policies or modified social distancing policies targeted to certain areas of the campus to a broad decision to shut the campus down. Indeed, a reopening decision that was likely to be safe but not guaranteed to be so under a fixed set of interventions (testing frequency) can be made more safe by the ability to bring additional interventions to bear if needed.

Modeling decision makers’ ability to shut down the campus upon observing substantial transmission, Fig. 2 *E* and *F* shows outcomes under plans of the form “initiate campus shutdown if confirmed cases exceed more than *Y* cases in *X* weeks” as a function of the pessimism level. While simpler than the more nuanced decision-making process available to leaders in practice, these plans take a simple interpretable form to support broad understanding and are reminiscent of cumulative sum control charts used for monitoring industrial processes for defects (44). They essentially “learn” whether reality is such that infection control is not being provided, and then respond by shutting down when a threshold is reached. Varying *X* and *Y* trades acting quickly against “false alarms” in which the campus would be shut down when infection control would have been achieved had it stayed open. Fig. 2 *E* and *F* demonstrates that adding recourse through shutting down can reduce the risk of reopening. Average total infections are eventually decreasing with pessimism level because the shutdown happens more quickly at high pessimism levels.

### Virtual vs. Residential Instruction.

While substantial focus was given to what would happen if universities reopened for residential instruction in the late summer of 2020 (35–39), an equally important consideration is what would happen if they did not. If a university chooses to offer only virtual instruction in place of in-person classes, many students may still elect to return to the university’s local area. This was the case at Cornell, where a significant number of students had signed leases with local landlords for fall 2020 before the severity of the pandemic became clear, and a survey revealed that many students were planning to return to Ithaca even if residential instruction and on-campus housing were not offered (20, 34).

For universities like Cornell, located in college towns, where the student population is significant relative to the overall population, this influx of students may represent a significant increase in the number of young people in the area. This could be dangerous because these young people may be socially cohesive, and, during normal times, young people have elevated rates of social contact (5, 45). Moreover, a university may have reduced ability to mandate and enforce behavioral restrictions and asymptomatic screening for students taking classes virtually even if they are nearby.

Thus, when deciding whether to reopen, in addition to whether the number of infections can be kept reliably low during a residential semester, an additional key consideration is the risk of an outbreak among virtual instruction students. In other words, a key trade-off is whether to invite back all students and have stronger behavioral and screening interventions, or to have a smaller number of students return but have weaker interventions.

To study this trade-off, we extended our model to capture virtual instruction at Cornell’s Ithaca campus. Under virtual instruction, staff and faculty, along with some research-focused graduate students, stay on campus. They are tested 2×/wk and are subject to the same behavioral compact governing student behavior under residential instruction. We also model some other students returning to Ithaca to live while taking classes virtually, outside of the control of the university. We assume that Cornell offers twice-weekly testing to these students, but noncompliance is higher than in a residential semester.

This extended model included four additional parameters, about which we also had uncertainty (*SI Appendix*, Table S21). We thus extended our parameter uncertainty framework to understand the range of outcomes possible under virtual instruction. We first generated ranges for these additional parameters based on information available in June 2020 (*SI Appendix*, section 3.A), extending our prior probability distribution to include independent normal priors for these new parameters. We also extended our nominal scenario for residential instruction to set each of these additional parameters to the center of its range.

In this extended model, reduced population density lowers transmission for unmonitored off-campus students relative to off-campus students during residential instruction, but lower compliance with social distancing and masks raises transmission. These effects can offset each other, or one can be more dominant, depending on parameters. Reduced use of now-optional testing blunts its benefits. Fig. 3*A* shows that reduced use of testing by the off-campus population can lead to a substantial number of infections, while outcomes are less sensitive to the number of off-campus students.

To understand the relative safety of residential and virtual instruction under plausible parameter configurations, we sampled 200 parameter configurations from the prior. Fig. 3*B* then plots the number of infections under residential and virtual instruction for each of these configurations. We see that infections are fewer under residential instruction in almost all parameter configurations. In those parameter configurations where there are more infections under residential instruction, the number of additional infections is small. This suggests that residential instruction is a safer strategy than virtual instruction, given the information available in June 2020.

Exploring further, we extended each of the 2,000 parameter configurations used for our residential infection sensitivity analysis (*Results C*) to include the four additional virtual instruction parameters (*SI Appendix*, section 3.E) and used simulation to predict virtual instruction infections (in the on-campus students and employees and the off-campus virtual instruction students in Ithaca) under each parameter configuration, enabling a comparison with predicted residential instruction infections for each configuration.

We then identified a new collection of parameter configurations of varying pessimism about the relative safety of residential instruction compared to virtual instruction. To do so, we adopted the same approach used to identify configurations of varying pessimism about residential infections but taking our primary outcome as the difference in infections between residential instruction and virtual instruction (a positive value indicates residential has more infections) ( *SI Appendix*, section 3.A). We refer to this difference as residential-virtual infections. The nominal scenario remains the same and corresponds to pessimism level 0.5. We obtain a new pessimistic scenario, corresponding to pessimism level 0.99. Unlike the pessimistic scenario for residential infections, this new pessimistic scenario (*SI Appendix*, Table S22 and Fig S15) decreases *R*_0_ relative to nominal. This is because the most likely parameter configurations with large residential - virtual infections (according to the fitted linear model) are those in which transmission is small regardless of instruction method.

Fig. 3*C* plots residential and virtual instruction infections for the two families of parameter configurations, one varying our pessimism about residential infections and the other varying our pessimism about residential - virtual infections. In almost all scenarios, virtual infections are larger than residential infections, and are sometimes much larger. Those few scenarios where residential infections are larger have few infections in both modes of instruction.

Thus, modeling suggests that virtual instruction presented a substantial risk, while residential instruction would result in lower infection counts under a broad range of the most reasonable parameter settings. This was a primary basis for Cornell’s decision to reopen for residential instruction (20).

### Design of Asymptomatic Testing Protocol.

Our modeling approach was also an important tool for supporting detailed design of Cornell’s surveillance testing strategy.

A first key question was the testing frequency for students and employees. Operational constraints limited the total number of tests that could be completed per day. We hypothesized that targeting more frequent testing to those groups likely to have higher rates of transmission would provide more robust infection control within this constraint.

**Fig. 4.**
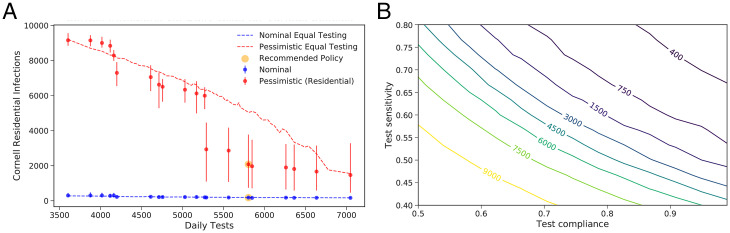
(*A*) Select Pareto-efficient testing policies (frequencies for each group) according to median simulated infections. Vertical bars depict the distribution of infections, ranging from the 10% to the 90% quantile over simulation outcomes. The point highlighted in yellow corresponds to the testing frequencies Cornell selected for the fall semester. Policies shown are Pareto-efficient policies from the set of policies where each on-campus group is tested either once or twice a week. The dotted lines are simulation estimates of the expected number of infections if tests are split homogeneously among the on-campus population. (*B*) Contour plot showing the number of infections in the Cornell community as test compliance and test sensitivity vary under the pessimistic (residential) scenario.

We enumerated testing policies consisting of a testing frequency (1× or 2× per wk) for the six groups spending significant time on campus (e.g., undergraduates living on campus) under both the pessimistic (for the residential infections outcome) and nominal scenarios, producing 64 testing policies. We then discarded those policies not on the Pareto frontier under the (residential) pessimistic scenario. Fig. 4*A* shows the resulting Pareto frontier and highlights the policy that was selected.

A second key question was the sampling methodology for surveillance testing. We considered AN and NP sampling. While NP is more sensitive (46), it is also less comfortable, and we hypothesized that this discomfort might lower test compliance. Fig. 4*B*, generated using our simulation under the pessimistic (residential) scenario, evaluates this trade-off between test sensitivity and test compliance. AN was chosen for Cornell’s surveillance sampling methodology in part based on this analysis, because the risk of a substantial loss in test compliance caused by NP sampling would not be known until after the launch of the program, while the test sensitivity of AN was measured before launch and was known to be sufficient for robust infection control.

### Retrospective View of Fall 2020 to Spring 2021.

**Fig. 5.**
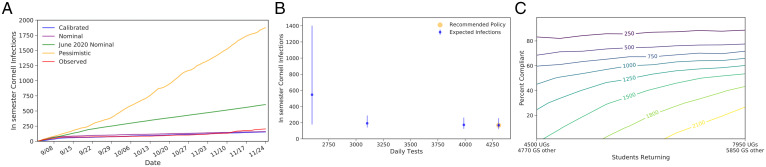
(*A*) Cornell fall 2020 cases relative to the calibrated trajectory and to the nominal, pessimistic, and June 2020 nominal scenarios. (*B*) Recreating the Pareto-efficient testing frontier based on calibrated parameter values. (*C*) Using calibrated parameters to recreate the contour plot showing expected Cornell infections (including students) under a virtual instruction scenario as the number of returning students and their test compliance varies; UGs, undergraduates; GS, graduate students.

We use the model calibrated in *Results A* to retrospectively evaluate the quality of the projections and decisions made in summer 2020 for the 2020–2021 academic year, focusing on two measures of quality: consistency with the range of plausible scenarios identified earlier and quality in hindsight of the decision made. We picture these results in Fig. 5.

First, we evaluate whether the calibrated model is consistent with the range of likely scenarios identified in *Results C*. A comparison of calibrated parameters (*SI Appendix*, Table S25) shows that the calibrated parameters are consistent with our prior. Fig. 5*A* compares observed infections to a range of scenarios. Observed infections were close to the nominal scenario and well under the pessimistic scenario for almost the entire semester, consistent with their design.

While the observed values are quite close to the nominal scenario’s predictions, this was accidental. Indeed, it was clear, a priori, that model outputs are sensitive to inputs, and these inputs were unknown, so the predictive accuracy of the range across scenarios, in the sense of whether it contained reality or not, is more important than whether a point prediction was close to the realized trajectory.

Fig. 5*A* also includes predictions from the nominal scenario in our June 2020 report (42). This scenario was nearly identical to the nominal scenario reported here, except that three key parameters were set to conservative values, given the urgent need to generate recommendations without enough time to identify a plausible range for these parameters (*SI Appendix*, section 3.C).

Second, we study the quality of the decisions made, relative to potentially better decisions that could have been made with the benefit of hindsight. We focus on two key decisions: the design of the testing policy and whether to reopen campus.

Fig. 5*B* shows the expected number and range of Cornell infections under Pareto-optimal testing policies, testing each of the three student groups from the retrospective analysis at either 1× or 2× per wk, and testing employees at a frequency averaged across those used in practice. Retrospectively, we selected one of many testing policies resulting in few infections, but we did not select the most efficient. The most efficient testing policies require segmenting those with the most transmission (who our data suggest are often in Greek-life organizations or are varsity athletes), which we did not recognize at the beginning of the semester. The figure also shows that there would have been limited benefit in increasing the total testing capacity.

Turning to the question of whether to reopen campus, Fig. 5*C* shows the expected Cornell infections for a virtual fall semester under our calibrated model, varying the number of returning undergraduate students and their test compliance. More than 80% of the returning students would need to remain test compliant throughout the entire semester to achieve a number of infections comparable to reopening campus (where fewer than 250 occurred). As discussed previously, enforcement of test compliance would have been significantly more challenging in a virtual scenario, and, therefore, the decision to reopen campus was robust.

While the benefit of hindsight would have modestly improved testing efficiency, the decision we selected gave health outcomes similar to the best policies possible with hindsight.

### Analysis for Fall 2021.

We further analyzed the need for asymptomatic screening to protect the campus population during the fall 2021 semester at Cornell, during which the Delta variant was widespread, vaccines were mandatory for students (but not for employees), and masking was required on campus, but social distancing measures were relaxed, and classes were operated in-person with normal density. Our analysis also extends to similar universities with varying vaccination rates. Here we provide a summary. *SI Appendix*, section 4 provides further detail.

During summer 2021, the appetite for aggressive interventions had softened, partly due to the prevailing confidence in vaccination, and Cornell’s capacity in both the testing laboratory and contact tracing was reduced relative to the previous academic year. Modeling nevertheless suggested that an outbreak of the Delta variant in a fully vaccinated subpopulation of highly social students was possible in the fall 2021 semester. Balancing this against test capacity, once per week testing for vaccinated students and twice per week for unvaccinated students was planned. (Although vaccines were mandated, students might become vaccinated after arrival or receive medical or religious exemptions.) As the start of the semester approached, studies emerged showing lower vaccine effectiveness (47–50) than previous studies, suggesting that the decision to test vaccinated students at the start of the semester was prudent but that testing all vaccinated students once per week might not be sufficient. Analysis of case counts upon student return led to changes initiated on August 29, 2021 increasing test frequency for students in Greek-life organizations, strengthening enforcement of test compliance, and adding contact tracing personnel to reduce the time from sampling to isolation of a positive case from 2 d to 1 d.

We leveraged the infection counts observed over this period and later into the fall 2021 semester within a Bayesian analysis to understand the effect of asymptomatic testing frequency and vaccination rate. We additionally leveraged parameters calibrated to the 2020–2021 academic year data reported above, and information from the scientific literature on vaccine effectiveness and the transmission under the Delta variant.

We began by elevating transmission rates in our model by a factor of 2.5 to account for the Delta variant. We added parameters to account for the effectiveness of vaccines against 1) transmission and 2) susceptibility to infection. We modeled 95% of the Cornell student population as vaccinated, a level that was later exceeded once newly arriving international students were vaccinated.

Significant changes had occurred since previous semesters: Social distancing restrictions had been lifted, prevalence outside the campus had changed, contact tracing faced a larger number of contacts per trace (meaning less attention can be paid to each contact), and asymptomatic vaccinated close contacts were no longer required to quarantine. This created uncertainty in several parameters: transmission rates within and from outside campus, and the number of cases quarantined or isolated per initiated contact trace. We placed a (joint) prior on these parameters, sampled parameters from that prior, ran simulations at each parameter set, and updated the prior to a posterior; see *Methods F* and *SI Appendix*, section 4.

**Fig. 6.**
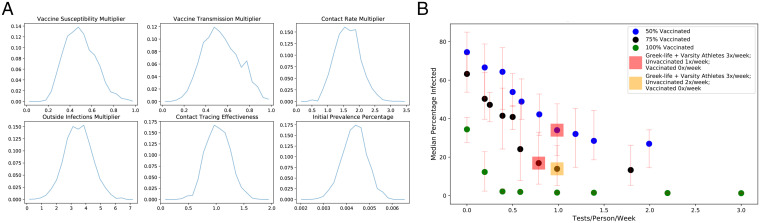
Fall 2021 modeling. (*A*) Marginal posterior distribution for the key parameters. Vaccine susceptibility and vaccine transmission are multipliers on the probability of becoming infected given exposure and the probability of infecting, respectively, compared to interaction between an unvaccinated source and exposed individual. The contact rate multiplier, outside infections multiplier, and contact tracing effectiveness parameters are all multipliers on the calibrated 2020–2021 parameters. (*B*) Estimated median cumulative infections over the semester for 50%, 75%, and 100% student vaccination levels versus the average number of tests per student per week, under a collection of Pareto-optimal testing strategies.

Fig. 6*A* shows the marginal posteriors of each of the parameters. While there is substantial uncertainty about each of the first three parameters, the posterior on their product is more concentrated near 0.4 (*SI Appendix*, section 4D), suggesting that the net effect of vaccination and relaxing social distancing restrictions in a fully vaccinated population is to reduce transmission by 60%. We find that the rate of infection from outside campus is ∼3× higher than the same time in the previous year (the fall 2020 semester).

Sampling parameters from the posterior, we then simulated potential trajectories for infections over the fall semester. This enabled a comparison of the effectiveness of different testing frequencies under different vaccination levels, as might be seen at universities similar to Cornell; Fig. 6*B* shows results for 50%, 75%, and 100% vaccination. We see that a nonuniform testing policy that focuses on highly social students is highly effective, but that, if vaccination levels are not sufficiently high, then substantial infections can result irrespective of testing policy. *SI Appendix*, Fig. S21*B* includes additional results that show that, for 50% and 75% vaccination levels, testing students once per week brings the great majority of the benefits of testing that are possible, and, at 100% (and lower levels), there is still important benefit in some testing, even of vaccinated students.

In contemplating projected infections for vaccinated populations, including Cornell’s fall 2021 semester, it is important to keep in mind that vaccination substantially reduces the possibility that an individual COVID-positive patient will become seriously ill. Indeed, considering a vaccine whose estimated efficacy is 95% in preventing serious illness given infection (see, e.g., ref. 51), one needs 1/(1 – 0.95) = 20 cases in vaccinated individuals to create the same expected number of cases with serious illness as one case in an unvaccinated individual. Thus, the gap in health outcomes between different vaccination levels is larger than the gap in infections seen in Fig. 6*B*. Moreover, in Cornell’s overwhelmingly vaccinated population (96% as of October 9, 2021), the chance of a serious negative health outcome during the fall 2021 semester is likely lower than in the fall 2020 semester despite a higher projected number of cases.

## Discussion

### Use at Cornell.

As described above, our analysis played a significant role in the decision to reopen Cornell University in the fall of 2020. It also determined the overall testing frequency required to limit spread, as well as the refined policy of varying test frequency across university subpopulations, and was an important factor in the choice to use AN instead of NP swabs for screening.

The modeling effort played a central role in a broad range of other tactical and operational policy decisions. First, it was essential to estimate, and then plan for, quarantine and isolation capacity. In doing so, the model was used to understand the requirements both for the initial arrival period and for the stochastic evolution of demand for these resources as the semester unfolded. Analysis focused on understanding the distribution of the maximum number of rooms that would be needed at any one time and ensuring that Cornell would either have enough rooms on hand or could rent them from local hotels. Second, the model quantified the impact of shortening the time between collecting samples and isolating positive individuals, which supported the design of the testing infrastructure. Third, the Cornell academic calendar was restructured to limit impact of a potential “second wave”: In-person education ended prior to Thanksgiving, and the final 3 wk of instruction were given virtually. Similar to the primary question of virtual vs. in-person education, the epidemiological modeling demonstrated the imperative of continued surveillance testing for the Cornell community residing in Ithaca beyond Thanksgiving. Fourth, the model indicated the need to increase the frequency of surveillance testing for the most socially active students from 2× per wk to 3× per wk at the beginning of the spring 2021 semester. Fifth, the model was the basis for the summer 2021 decision to screen vaccinated students. Sixth, as cases grew quickly at the start of the fall 2021 semester, the model was used to make the case for prioritizing additional contact tracing staffing to reduce test delays and to elevate testing frequency for students in Greek-life organizations. Upon implementing the interventions, daily cases fell dramatically.

Finally, the modeling effort also played an important role in enhancing communication with the community—as a tool for explaining the considerations in making decisions, and for explaining what might happen and why.

### Use at Other Universities.

Considering other universities, we find that testing students, on average, once per week, even in the presence of high vaccination rates, is important in limiting infections when the Delta variant is dominant. Further testing beyond that level can help, but, for maximum benefit, it is sufficient to target this testing to the most social students.

This finding is consistent with student outbreaks observed in cohorts with high vaccination rates that were being tested once per week at Duke and Harvard Universities (52, 53).

When applying results contingent on vaccination level, keep in mind that our analysis assumes unvaccinated individuals have immune systems naive to SARS-CoV-2. Infection-acquired immunity shares at least some characteristics with vaccination, in that (future) infection probability and severity if infected is lowered (54). Moreover, many universities had large outbreaks in the 2020–2021 school year, and the most social students on campus would have been among the most likely to be infected. Thus, in applying our results, a university’s level of immunity may be higher than what its vaccination level would suggest.

Our finding that there is little or no interaction leading to transmission between Cornell university employees and students is important for understanding and mitigating risks to employees at other universities. At Cornell, we have found that the biggest risk to employees is through their interactions outside the university.

Our finding that a small group of highly social students plays a large role in viral transmission in the Cornell student population may be important for designing interventions at other universities. If a comparable group can be identified at other universities and specific interventions brought to bear that prevent spread within this group, then this may provide outsized protection for the resources required.

In applying our results to other universities in the future, it is important to keep in mind several specific characteristics of the Cornell population and environment that lent themselves to the strategies developed. 1) Beginning early in the pandemic, Cornell’s College of Veterinary Medicine had substantial capacity to provide PCR testing, exploiting pooling. 2) Ithaca has had lower prevalence than much of the rest of the country and is several hours’ drive from major cities. 3) Cornell’s student population is largely residential, with few commuting from nearby communities. 4) Cornell’s students have been largely willing to comply with social distancing, masking, and testing policies. 5) Cornell has access to a hotel on campus and other buildings that have been used for quarantine and isolation capacity.

Beyond universities, our modeling framework can be applied to other closed (or nearly closed) communities such as cruise ships, prisons, retirement homes, homeless shelters (55), military bases, or professional sports bubbles. While differences in the behavioral and health characteristics of the populations involved, as well as the interventions available, would require adjustments to model parameters, any of these relatively closed communities could be modeled and analyzed in a similar way, coping with parameter uncertainty as discussed here.

Like most epidemiological modeling, ours has limitations. Most importantly, our results are highly sensitive to input parameters for which there was significant uncertainty. Although our results showed that our conclusions were remarkably robust, our June 2020 nominal scenario was cautious in selecting conservative values for parameters, based on literature and other relevant data. Cumulatively, this caused the predictions we released in the summer of 2020 to be conservative. Conversely, the interventions we helped design over the summer of 2021 in preparation for the 2021 fall semester needed modification in late August before infections seen in the early part of that semester were fully controlled.

Beyond parameter uncertainty, our model has limitations due to its simple structure. For example, we assume that all members of a subgroup are homogeneous, and, therefore we do not model contact network structure. As a result, while it is similar to that used in other analysis (19), our model of contact tracing has limitations; for example, we assume that all quarantined contacts are in the preinfectious exposed state. Further, within a single compartment, we assume that infectiousness does not vary across individuals or over time, in contrast to, for example, ref. 7. Moreover, we model the impact of age and its effect on the distribution of infection severity in a discretized way. We do not model overdispersion in contacts (for example, one individual having many more contacts than another), because our estimated transmission parameters are based on empirical *R*_0_ estimates often driven by the most social individuals. When infections are driven by spread among subpopulations with the highest transmission, empirical estimates of *R*_0_ naturally correspond to transmission rates in these subpopulations.

## Methods

Here we provide an overview; *SI Appendix* gives full details.

### Simulation Model: Overview.

We model COVID-19 transmission using a compartmental model with Susceptible, Exposed, Infectious, and Recovered compartments along with additional compartments to reflect specific characteristics of COVID-19 and the interventions applied (Fig. 1*A*). To account for asymptomatic and presymptomatic transmission, the infectious phase is split into three compartments: Infectious, Asymptomatic, and Symptomatic. Individuals formerly in the Exposed compartment enter the Infectious compartment before randomly being assigned to either the Asymptomatic or Symptomatic compartments. We also add compartments for quarantined noninfected individuals (Quarantine) and isolated infected individuals (Isolation). Due to significant age and social heterogeneity in a university community, we replicate the compartments described above for each of several university groups. The groups interact via cross-group contacts. In addition, since the severity of COVID-19 varies significantly with age, the probabilities of symptom severities are determined by a group’s age distribution.

### Simulation Model: Transmission.

Modeled COVID-19 transmission is governed by two variables: contact rates between groups, represented by a contact matrix, and the probability of transmission during any interaction. The contact matrix is estimated using prepandemic measurements of age-based socialization patterns. The probability of transmission is calibrated to match external estimates of *R*_0_ (2.5 for the nominal scenario). Transmission is modeled as stochastic, and the number of new infections in each group has a Poisson distribution with a mean determined by the product of the contact matrix and the transmission probability and the number of nonisolated infectious individuals in each group.

### Simulation Model: Quarantine and Isolation.

We model three mechanisms for identifying and quarantining/isolating infected individuals.

1) Testing is the first mechanism. Every day, a fraction of each group is selected uniformly at random to be tested, and test results are available the same day. The number of individuals selected to be tested per group per day is determined by the group’s testing frequency. (This is a conservative approximation to what had been implemented, where each student is tested at a specified frequency.)
2) Symptomatic self-reporting is the second mechanism. Every day, each symptomatic individual not in Isolation has a constant probability of self-reporting their symptoms, upon which they enter Isolation the same day.
3) Contact tracing is the final mechanism. Each case found through testing or self-reporting is contact traced. (Cases found through contact tracing are not, themselves, modeled as contact traced. This is an approximation of reality that is necessary because our simulation tracks counts within groups, not individuals.) Each contact trace moves a Poisson-distributed number of people to Quarantine or Isolation after a 1-d delay. We assume that contact tracing only finds contacts within the same group as the source case, reflecting the social dynamics of college campuses.

### Parameter Configurations of Varying Pessimism.

To summarize the effect of parameter uncertainty on an outcome (infections in a residential semester, or the difference in infections between residential and virtual instruction), we developed a one-dimensional family of parameter configurations with varying levels of risk for each outcome. For each real number *y*, we consider the set of parameter configurations *A*(*y*) whose median outcome is equal to *y* according to the fitted linear model. (All configurations are in exactly one *A*(*y*), so this partitions the parameter configuration space.) For each *y*, we select a representative configuration from *A*(*y*): the one that is most likely under the prior. As we increase *y*, outcomes under the representative configuration tend to degrade. We then graph the outcome under this configuration versus $P(\cup_{y\prime \leq y}A(y\prime ))$, that is, the probability under the prior of seeing a parameter configuration whose outcomes (under the linear model) are no worse than those in *A*(*y*). We refer to this probability as the “pessimism level.” It ranges from zero to one, with larger values corresponding to representative parameter configurations that are more pessimistic. For details, see *SI Appendix*, section 3.A.

### Retrospective Parameter Estimation and Model Calibration.

We estimate most model parameters for the fall semester (initial prevalence, outside infection rate, a contact matrix with entries that are proportional to the intergroup transmission rates, and contact tracing effectiveness) directly from fall semester data available in operationally focused nonresearch public communications (56). These public health communications were based on deidentified positive case, testing, and contact tracing stored along with student life, housing, and employee data in a Health Insurance Portability and Accountability Act (HIPAA)–compliant database. These data were collected and analyzed pursuant to the urgent public health need presented by the pandemic. Additional aggregations of deidentified data for research purposes were provided by Cornell University and deemed by Cornell’s institutional review board to not meet the regulatory definition of human subjects research.

Remaining parameters, that is, the proportionality constant scaling the contact matrix for the student group and the rate of transmission for the employee group, were estimated by calibrating the model output to the infection trajectories observed during the fall 2020 semester. As already discussed, we consider employees and students to be two completely separate groups that do not infect each other. The calibration results are robust to the addition of a small but positive employee–student transmission term, although such a term can change outcomes more substantially in extreme regimes where student or employee prevalence is much higher than observed in the fall.

### Bayesian Analysis for Fall 2021 Decisions.

We leveraged information gathered from the fall 2020 semester but adjust for changes such as the Delta variant and vaccination level. Then, we identify a set of key parameters with uncertainty and perform a Bayesian analysis for fall 2021 projections based on recently observed student case counts at Cornell in the fall 2021 semester. Specifically, we placed a range on each of these parameters, which we treated as the symmetric 95% credible interval of a normal prior distribution. We then derived a truncated multivariate normal prior by limiting each parameter to its valid range. We then sampled parameter configurations from the prior distribution and ran simulations at each parameter configuration. To approximate the posterior distribution for the parameters, we used a lognormal likelihood function for the trajectory observed to date in the fall 2021 semester, with hyperparameters estimated from the sampled trajectories. Finally, we sampled parameters from the approximated posterior distribution and simulated trajectories based on these sampled parameter sets. These trajectories represent potential epidemic outcomes for the fall 2021 semester. For details, see *SI Appendix*, section 4.

## Supplementary Material

Supplementary File

## Data Availability

Code and simulation results have been deposited in GitHub, https://github.com/peter-i-frazier/group-testing (57). Data collected from Cornell students and employees during surveillance testing is HIPAA-protected and not approved for public release. Aggregates from this data approved for release are reported in the main text and *SI Appendix*.
