# Supplementary material for: Modeling for COVID-19 college reopening decisions: Cornell, a case study
Source: Proc Natl Acad Sci U S A. 2022 Jan 4;119(2):e2112532119. doi: 10.1073/pnas.2112532119 (PMC8764692; doi:10.1073/pnas.2112532119)
Supplement: Supplementary File [file pnas.2112532119.sapp.pdf]

PNAS
www.pnas.org

Supplementary Information for

Modeling for COVID-19 College Reopening Decisions: Cornell, A Case Study

P. I. Frazier, J. M. Cashore, N. Duan, S. G. Henderson, A. Janmohamed, B. Liu, D. B. Shmoys, J. Wan, and Y. Zhang

Corresponding Peter I. Frazier.
E-mail: pf98@cornell.edu

This PDF file includes:

Supplementary text
Figs. S1 to S22 (not allowed for Brief Reports)
Tables S1 to S28 (not allowed for Brief Reports)
SI References

Contents

1 Model **2**
A Individual Disease Progression . . . 4
B Severity of Symptoms . . . 4
C Contact Tracing . . . 6
D Outside Infections . . . 7
E Students Returning and Initial Prevalence . . . 7
E.1 14-day self quarantine . . . 7
E.2 Move-in weekend and low-prevalence states . . . 8
F Testing Details . . . 9
G Group Details . . . 9
H Transmission . . . 10
I Virtual Instruction . . . 12
J Matrix Input for Simulation . . . 14

2 Model Calibration **15**
A Model Calibration for Students in the Fall 2020 semester . . . 15
B Model Calibration for Employees in the Fall 2020 semester . . . 19
C Model Calibration for Students in the Spring 2021 semester . . . 21
D Model Calibration for Employees in the Spring 2021 semester . . . 25

3 Parameter Uncertainty **27**
A Parameter Scenarios . . . 27
B Assessing Pessimism Level of Parameter Scenarios . . . 30
C Scenarios from June 2020 report . . . 31
D Comparison of Prior to Calibrated Outcomes . . . 36
E Sensitivity Analysis for Individual Parameters . . . 38
F Correlation of Infection and Hospitalization metrics . . . 41

4 Bayesian Analysis for Fall 2021 Projections **42**
A Parameter Adjustments . . . 42
B Parameter Range Justification . . . 42
C Posterior Approximation and Projections . . . 44
D Supplemental Results for Fall 2021 Projections . . . 47

Supporting Information Text

This appendix is split into four sections. In the first section, we describe our model and methodology for estimating its parameters. Papers we reference in this section are from the summer of 2020 since this is when we were estimating the parameters for the fall 2020 semester. The second section relates to calibration of our model in the retrospective study. The third section shows the sensitivity of our model to varying input parameters. The last section describes a Bayesian analysis for fall 2021 projections.

Portions of this appendix have been previously released as part of the communication of our public health work (1–5).

Code implementing the simulations described is available at https://github.com/peter-i-frazier/group-testing.

1. Model

Model Overview. We model the spread of COVID in the Cornell and surrounding greater Ithaca community using a multi-group stochastic compartmental simulation model. Each group is modelled using a discrete-time Markov chain (DTMC) with the state described below. All these DTMCs are linked together by the transmission process.

- Number people in Susceptible
- Number people in Exposed with x days remaining until they become Infectious (ID) for x in $\{0, 1, ..., 7\}$
- Number people in Infectious with x days remaining until they become Symptomatic/Asymptomatic for x in $\{0, 1, ..., 8\}$
- Number people in Symptomatic with x days remaining until they recover for x in $\{0, 1, ..., 20\}$
- Number people in Asymptomatic with x days remaining until they recover for x in $\{0, 1, ..., 20\}$
- Number people in Recovered
- Number people in Quarantine

- Number people in Isolation
- Number people who will be contact traced in future days (allows us to account for contact tracing delay)

We only maintain counts of the aggregate number of people in each state, not the trajectories of each individual. We use the term 'free individuals' to refer to everyone not currently in Quarantine or Isolation. Similarly, we use 'free and infectious' individuals to refer to all free individuals that are Infectious, Symptomatic, or Asymptomatic.

Every day corresponds to 1 state transition of the DTMCs. The transition kernel reflects five key dynamics:

1. Natural disease progression of infected individuals
2. Surveillance testing
3. Symptomatic self-reporting
4. Contact tracing
5. Transmission and new infections

1. Natural Disease Progression. Figure S1 shows the compartments we use to model the progression of COVID. The probability that someone transitions from Infectious to Symptomatic depends on the age distribution of their group. Once someone has been infected, we assume that they cannot be re-infected.

The Isolation compartment is for isolated individuals who are infected and the Quarantine compartment is for quarantined individuals who are not infected. Once an infected person has been identified and isolated, they cannot create any new infections and leave quarantine/isolation after they are no longer contagious. Every day, each person in Quarantine or Isolation has a constant probability of being released (to Susceptible and Recovered respectively).

If a free individual is infected (Exposed, Infectious, Symptomatic or Asymptomatic) and not isolated, they transition from their current compartment with x days remaining to the same compartment with $x - 1$ days remaining. If there are no remaining days in their current compartment, they transition to the next compartment. At this time, the length of stay in their next compartment is realized and the state of the DTMC reflects this realization. Transitions from Susceptible to Exposed occur due to transmission events and at that time their length of stay in Exposed is realized.

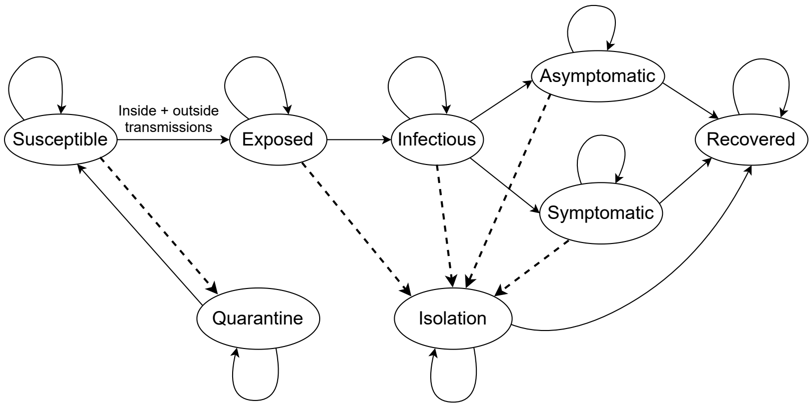

Fig. S1. Timeline of disease progression in an infected individual.

2. Surveillance testing. Every day a fraction of the group's free population is independently randomly selected. This fraction selected for testing can vary by group but is constant over the horizon of the simulation. We assume that people in compartments Infectious, Symptomatic and Asymptomatic are detectable by testing. Each test has a constant independent probability of producing an incorrect result (false positive or negative). False positives move people from Susceptible to Quarantine while false negatives do not change the state of the individual. True positives move people from an infectious state (Exposed, Infectious, Symptomatic or Asymptomatic) to Isolation. Test results are assumed to be available the same day. Each positive case identified through surveillance testing produces a contact trace.

3. Symptomatic self-reporting. Every day, each symptomatic individual has an independent, constant probability of self-reporting symptoms. Upon self-reporting, they are moved to Isolation and generate a contact trace. The probability of self-reporting every day is calibrated to data provided by the CDC.

4. Contact tracing. Each contact trace removes a random number of people from the free and infectious population and from the susceptible population. Symptomatic self-reports remove more susceptible and free and infectious people since these cases have likely been in the community longer than people identified via surveillance testing. We also assume a deterministic (1 day) delay between initiating a contact trace and isolating the contacts. The number of people and infectious cases removed is calibrated to Tompkins County contact-tracing data.

We do not contact trace positive cases found via contact tracing. Contact tracing only removes individuals in the same group as the source.

5. Transmission and new infections. We model two sources of new infections. The first is outside infections which refers to infections imported from interactions outside of Tompkins County. This is a daily rate per person estimated from travel-related Tompkins County cases.

The second source of new infections is local transmission due to free and infectious individuals. The rate of local spread is governed by two parameters: the contact rates between groups and the probability of transmission during an interaction. The contact rates are estimated using pre-pandemic contact surveys and account for age-varying compliance with wearing a mask and social distancing. The probability of transmission is calibrated to match the R0 of the disease.

Model Details. We first discuss the intra-group dynamics (disease progression, symptom severity, contact tracing, surveillance testing) followed by inter-group dynamics (transmission).

Intra-group Dynamics.

A. Individual Disease Progression. Our simulation assumes that the disease progresses through several stages in each infected individual, represented in Figure S1.

Parameters for the length of time in each state are given in Table S1.

Table S1. Parameters for disease progression in an individual.

Parameter description	Nominal parameter value(s)	Sources
Time from exposure to infectious	Poisson(2) days	(6); (7); (8); (9)
Time from infectious to symptom onset	Poisson(3) days	
Time in symptomatic state	Poisson(12) days	(10)
P(self-report each day | asymptomatic)	0	Conservative assumption
P(self-report each day | symptomatic)	0.22	CDC planning scenario (11)

To justify the choice of time in the Exposed and Infectious states: (6) does a pooled analysis and finds the median incubation period to be 5.1 days, with a confidence interval of 4.5 to 5.8 days. (9) and (7) find that transmissions can occur 2-3 days before symptom onset. Thus we set the time in the Infectious state to be Poisson(3), and subtract its mean (3 days) from the incubation period mean to get a mean of 2 days for the exposed state.

In the simulation we model the time to self-report symptoms (for symptomatic patients) as being geometrically distributed with a single parameter that is the probability of self-reporting each day. This was chosen to match the average time from symptom onset to hospitalization for influenza-like illness (ILI) according to the CDC (11), which is based on (12). The latter paper reports that

- 35% of symptomatic individuals seek care in ≤ 2 days,
- 47% of symptomatic individuals seek care in $3 - 7$ days,
- 18% of symptomatic individuals seek care in ≥ 8 days.

We model this as a random number of days that is conditionally uniform(0,2) with probability 35%, conditionally uniform(3,7) with probability 47%, and conditionally uniform(8,12) with probability 18%. The resulting mean of this distribution is $.35 \times 1 + .47 \times 5 + .18 \times 10 = 4.5$ days. The daily probability of self-reporting for symptomatic individuals is then chosen to be $1/4.5 \approx 0.22$ so that the mean time to self-report, $1/0.22 = 4.545$, approximately matches this value.

B. Severity of Symptoms. Our simulation model separates symptomatic from asymptomatic individuals. Over the course of the simulation, symptomatic individuals self-report each day with some probability, while asymptomatic individuals do not self-report. Symptomatic infections can be of different levels of severity, ranging from mild pneumonia symptoms to critical life-threatening conditions. Thus we divide the symptomatic individuals into three different severity levels. In total, we consider four different severity levels, defined as follows:

- Severity level 1: patient is asymptomatic.
- Severity level 2: patient shows mild symptoms, but does not require hospitalization.
- Severity level 3: patient needs to be hospitalized, but does not require intensive care.

- Severity level 4: patient requires intensive care.

At the end of each simulated period, we allocate the symptomatic individuals to severity levels 2-4 with certain proportions. These proportions are estimated from data as explained below. Once an individual is assigned to a severity level they remain there; further transitions between severity levels are not modeled.

Let $P(\text{sev } i)$ be the probability that, as a result of a single contact with an infected person, an individual becomes infected and falls within severity level i. Thus the sum of these probabilities over $i = 1, 2, 3, 4$ is the probability of infection as a result of a single contact. Then, the probabilities that as a result of a single contact an individual becomes infected and asymptomatic, respectively infected and symptomatic, are

$$P(\text{asymptomatic}) = P(\text{sev } 1), \text{ and}$$
$$P(\text{symptomatic}) = P(\text{sev } 2) + P(\text{sev } 3) + P(\text{sev } 4).$$

We want to find $P(\text{sev } i)$ for the population while considering age-based factors. Specifically, we model how the severity of the disease varies with age, and that older age groups are more likely to become infected after an interaction with an infectious person. To that end,

$$P(\text{sev } i) = \sum_{\text{age}} P(\text{sev } i|\text{infected, age})P(\text{infected}|\text{age})P(\text{age}), \text{ where}$$
$$P(\text{infected}|\text{age}) = P(\text{infected}|\text{contact, age})P(\text{contact}|\text{age}) \propto P(\text{infected}|\text{contact, age}).$$

The proportionality in the second equation comes from the assumption of a homogeneous well-mixed population within each group. Therefore, the distribution of the age of contacts is the distribution of the age of the population in the group.

Severity Calculation Part 1: Severity and Infection given Age We obtain values for the probability of infection as a function of age from (13), which reports the probability of infection through a close contact for different age groups among 4941 close contacts traced from early cases in Guangzhou, China. These estimates are given in the first row of Table S2.

Later, we will estimate the age distribution ($P(\text{age})$) for Cornell's fall semester.

Table S2. Parameters for age-stratified infection probability and severity level distribution. Sources: (13–17).

	Age grp 1 (0-17)	Age grp 2 (18-44)	Age grp 3 (45-64)	Age grp 4 (65-74)	Age grp 5 (75+)
P(infection | age)	1.8%	2.2%	2.9%	4.2%	4.2%
P(sev 1 | infected,age)	17.0%	52.0%	31.0%	13.0%	13.0%
P(sev 2 | infected,age)	81.6%	47.2%	65.9%	80.6%	80.6%
P(sev 3 | infected,age)	1.1%	0.6%	2.2%	4.7%	4.7%
P(sev 4 | infected,age)	0.3%	0.2%	0.9%	1.7%	1.7%

The severity level distribution for each age stratum is estimated from a combination of data sources.

We first estimate $P(\text{sev } 1|\text{infected, age})$, the asymptomatic rate for each age group, as follows.

1. Fix the asymptomatic rate for the 75+ age group, $P(\text{sev } 1|\text{infected, age grp } 5)$ to 13%. The 13% figure comes from (18), where a nursing home in Seattle had 3 asymptomatic cases out of 23 confirmed cases.

2. To estimate the asymptomatic rate of the remaining four age groups, we attempt to match the following data points by minimizing the sum of squared errors, subject to the (assumed) constraint that the asymptomatic rates decrease over age groups 2 through 5.

   (a) The CDC estimated that the population asymptomatic rate in the USA was 35% (Source: (11)). Weighting our age-stratified asymptomatic rates by the age distribution for the US population we should obtain a value close to 35%. (Sources for age demographics: (19) and (20).)

   (b) The Diamond Princess cruise ship had an estimated 17.9% asymptomatic rate (Source: (21)). Exactly as we did for the CDC US-population rate, we use age strata for the infected passengers on the Diamond Princess to attempt to match the 17.9% rate.

   (c) A study of 78 infected patients from Wuhan had the following age profile for the 33 asymptomatic patients: 25th percentile: 26 yrs, 50th percentile: 37 yrs, 75th percentile: 45 yrs (Source: (22)). We attempted to match these percentiles. We use the age demographics of China for this purpose. (Source: (23).)

To this point then, we have estimated the asymptomatic rate for each of the 5 age groups, $P(\text{sev } 1|\text{infected, age})$. We next divide the remaining probability within each age group into severity levels 2, 3 and 4 using CDC numbers for hospitalization rates and ICU rates in the nominal planning scenario (11). By our definition, hospitalization includes both severity levels 3 and 4, and ICU corresponds to severity level 4. The three equations we need for the three unknowns (probability of each of severity levels 2, 3 and 4) are

1. $P(\text{symptomatic}|\text{infected,age}) = P(\text{sev } 2, 3, 4|\text{infected,age}) = 1 - P(\text{sev } 1|\text{infected,age})$.
2. Given that a patient is symptomatic, the probability they will be hospitalized is
$$P(\text{sev } 3, 4|\text{infected,age})/P(\text{symptomatic}|\text{infected,age}).$$
3. Given that a patient is hospitalized, the probability that they will be admitted to the ICU is
$$P(\text{sev } 4|\text{infected,age})/P(\text{sev } 3, 4|\text{infected,age}).$$

The CDC (11) estimates the symptomatic case hospitalization ratio to be 1.7% for age 0-49, 4.5% for age 50-64, and 7.5% for ages 65+. The percent admitted to ICU among those hospitalized is 21.9% for age 0-49, 29.2% for age 50-64, and 29.8% for ages 65+. We recognize that the age cutoffs are slightly different to ours. We match the CDC's estimates for age 0-49 to our first two age groups, those for age 50-64 to our second age group, and those for 65+ to our fourth and fifth age groups. The probabilities of severity levels 2, 3, 4 are calculated accordingly to fit these estimates.

Severity Calculation Part 2: Age Distribution To complete our severity calculation, we first identify different groups on Cornell's campus and estimate their distribution over the five age groups. The parameter values are given in Table S3.

Table S3. Information for different population groups on Cornell's campus. The size of each group as well as the faculty age distribution are provided by (24); the age distribution for academic professionals, staff, and students are assumed.

	Group size	Age group 1 (0-17)	Age group 2 (18-44)	Age group 3 (45-64)	Age group 4 (65-74)	Age group 5 (75+)
Faculty	1684	0%	33.1%	46.1%	17.9%	2.9%
Academic professionals	1114	0%	90%	10%	0%	0%
Staff	7485	0%	50%	50%	0%	0%
Students	24027	0%	100%	0%	0%	0%

For the Fall reopen, each of the 7 Cornell groups has an age distribution based on the table above. This age distribution dictates the severity distribution for each group. We assume that the remaining group (Greater Ithaca) has the same age distribution as the US population.

C. Contact Tracing. In our model, each positive case identified through self-reporting and a fraction of cases identified through asymptomatic surveillance initiates a contact trace. Contact tracing is not recursive, in that we do not model contact tracing of cases identified in a contact trace. This is for simplicity, but also because the number of contacts of those identified in a contact trace are likely to have had fewer contacts than those identified by self reporting or asymptomatic surveillance, since their detection was not triggered by one of these two mechanisms. (Here we use the term "contact" in the sense of potentially leading to infection, rather than a more restrictive sense used by the Tompkins County Health Department (TCHD).) Our model of contact tracing is necessarily simplistic, since we do not model individuals and their contact networks in our compartmental simulation.

Every positive case identified through self-reporting initiates a contact trace. Each contact trace results in some number of individuals isolated and quarantined. We take the number of isolations per contact trace to be a Poisson random variable and the number of quarantines per contact trace to be a constant. We assume that the contacts of each positive case do not overlap, so in generating the total number of individuals isolated or quarantined based on, e.g., n new positive cases identified through self-reporting, we can simply generate a single Poisson random variable with a mean that is n times that for a single case. It remains to specify the mean of the Poisson random variable for the number of isolations per initiated contact trace, and the constant number of quarantines per initiated contact trace. We assume that the positive case has had, on average, c contacts per day for t days, for a total of ct contacts. Contacts are infected independently of one another with probability p. Contacts, whether infected or not, are assumed to be remembered by the positive case with probability r. The value of p is estimated to be 1.8% in Section H below. The value of c is on the order of 12 or 13, depending on the group, as discussed in Section H below. Given that the positive case self-reported, they must be symptomatic, and so t is taken to be the sum of the means of the times in the Infectious and Symptomatic states. Under our nominal parameters, this gives $t = 3 + 1/0.22 = 7.55$ days. The value of r is taken to be 0.5, in line with anecdotal evidence from the TCHD. Accordingly, the expected number of contact-traced infected contacts is $ctpr = 0.85$. It is reasonable to expect the expected number of contact-traced non-infected contacts to be $ct(1-p)r = 46.3$, but this number reflects a great deal of double counting of individuals. Anecdotal evidence from TCHD suggests that on the order of 7 individuals are identified through contact tracing on average, suggesting that the number of contact-traced non-infected contacts should be taken to be $7 - ctpr = 6.15$ under nominal parameters. We adopt this figure instead.

Positive cases identified through asymptomatic surveillance are modeled in the same manner, except that cases identified in this manner would typically be identified earlier in the course of their disease, at which point they would have infected fewer people. We model this by only initiating contact traces for a fraction of the positive cases identified through asymptomatic

surveillance. We take the number of contact traces initiated on each day to be Poisson with mean $N/2$, where N is the number of surveillance positives from the relevant day.

All infected cases identified through contact tracing are pulled from the Exposed, Infectious, Symptomatic and Asymptomatic states, in that order of precedence, and enter the Isolation state. All non-infected cases identified through contact tracing are pulled from the Susceptible state and enter Quarantine.

Table S4. Parameters for contact tracing.

Parameter description	Nominal parameter value(s)	Sources
Fraction of contacts recalled, r	0.5	
Contact tracing delay	1 day	(25)
Contact traces initiated per screening positive	0.5	
Contact traces initiated per self-report positive	1	
(Implied) New isolations per initiated contact trace	0.85	Calculation in text
(Implied) New quarantines per initiated contact trace	6.15	(25)

D. Outside Infections. We estimate the probability of outside infection per person per day, which arises from infections imported from outside the modeled groups due predominantly to travel outside Tompkins County. TCHD data reports 13.2 travel-related COVID cases per month from March 2020 to July 2020. The asymptomatic rate at that time was estimated to be approximately 50%, so the actual number of cases is estimated to be twice this number, or 26.4 cases per month. Assuming that during this period there were 75,000 people in Tompkins County, we arrive at a figure of $26.4/30/75,000 = 1.2 \times 10^{-5}$ for the probability of outside infection per person per day.

An additional source of outside infections comes from students returning at the start of the fall semester, which we model next.

E. Students Returning and Initial Prevalence. In advance of the fall 2020 semester, New York state required all travellers from high-prevalence states to self-quarantine for two weeks upon arrival. The list of high-prevalence states changed throughout August 2020, in advance of the Fall Semester. Our analysis is based on New York State's list of High Prevalence states on August 7, 2020. We model the return of students to campus in two phases: (1) a 14-day period when students from high-prevalence states arrive and self-quarantine, followed by (2) move-in weekend when other students arrive.

The modeled student arrival process is summarized below.

- Some students get tested remotely and are isolated if positive. Others come without being tested. Students coming from high-prevalence states are less likely to have test access at home.
- Students traveling to campus risk additional infection after being tested at home prior to departure (if they are tested) and during travel.
- Students are required to be tested upon arrival as a condition for enrollment. Students are strongly encouraged to use the first available testing date, though some will instead choose to be tested later. Positives are isolated, including some false positives. If a student comes from a high-prevalence state, then the student is required to self-quarantine for 14 days.
- Some positive cases already exist on campus due to infections from the greater Ithaca area.
- Some positive cases among incoming students are missed because of false negatives and because some students are early enough in their infection to not be PCR-detectable.
- These two sources of cases (existing and new) combine to create an on-campus prevalence.
- This on-campus prevalence creates additional cases on campus. Some additional cases are also created on campus due to outside infections from the greater Ithaca area.
- During the two-week period before the move-in weekend, regular surveillance testing had not begun, but contact tracing was underway.

E.1. 14-day self quarantine. Here we discuss the model for the arrival of students from high-prevalence states for which New York State requires a mandatory 14-day self-quarantine. The students among these that have access to housing in which they can self-quarantine are modeled as arriving in Ithaca two weeks before classes start. Other students in this group without such housing are modeled as either choosing to start classes virtually or, in a few cases, coming to Ithaca without complying with the required quarantine period in violation of state law.

Incoming Student Population Sizes: Student data suggested that roughly 33% of the undergraduate students and 23% of the graduate / professional students have homes in states designated by New York State as "high prevalence" requiring mandatory quarantine.

We assume that many such students with off-campus housing will spend the mandatory quarantine period in Ithaca in that housing. For students that originally planned to be in on-campus housing, we assume that the majority will not come to

Ithaca at the start of the semester but rather will begin the semester online; a small fraction will quarantine somewhere outside Ithaca and return during the move-in weekend; while another small fraction will fail to comply with the law, either using non-compliant quarantine in shared housing in Ithaca, or by arriving during move-in weekend without having quarantined. Assuming that 10% of continuing undergraduates and 75% of continuing graduate / professional students have stayed in Ithaca, the total number of students arriving 2 weeks in advance from high prevalence states is 3750, including 2500 undergraduate students and 1250 graduate / professional students.

Compliance: Despite the mandatory self-quarantine order, we do not assume full compliance. We estimate the daily transmission rate to be reduced by 40% compared with the nominal setting. We do this to model several kinds of non-compliance with quarantine. First, some students required to quarantine may do so in non-compliant locations shared with others. Second, some students may break quarantine and have social interaction. Third, although students were asked to test on arrival (so that positives can be isolated and monitored, reducing the danger of transmission), testing was offered only three times a week so there may be a delay between arrival and the first available test date.

Testing Before Departure: Cornell students were asked to test before departing to come to campus, but this was not mandated due to a lack of test access for some students. We assume that $\frac{1}{3}$ of students from high-prevalence states were tested at home, and $\frac{2}{3}$ from low-prevalence states, both using nasopharyngeal (NP) sampling with 90% sensitivity (26).

Testing on Arrival: As discussed above, we assume that students are tested once on arrival. We assume NP sampling with 100% compliance. Because the semester had not begun, and mandatory asymptomatic screening had not started, we assume that no other testing is done.

Prevalence Estimation for High-Prevalence States: Prevalence at the origin of students from high-prevalence states is assumed to be 4%. This estimate was obtained by multiplying daily new positive cases, an underreporting factor (assumed to be 10, i.e. for each reported positive case there are 9 positive cases not reported), and the average number of days an infected individual is active (assumed to be 20).

Population Already in Ithaca: The total number of students that either stay in Ithaca during the summer or come to Ithaca early from other “low prevalence” states is estimated to be 4090 (including 1130 undergraduate students, 2960 graduate / professional students). All 10280 employees are assumed to remain in Ithaca throughout the summer. The prevalence among the group of unquarantined students and the group of employees is assumed to be 0.1%, which is consistent with the estimated persistent prevalence level in the greater Ithaca area during summer 2020. (See below)

Assuming 31 confirmed cases, which is what was observed over the first 21 days of July 2020, that cases last 20 days, and 2x-4x underreporting in Tompkins County (less than elsewhere due to excellent testing access), gives 60 - 120 active cases, or 0.075% - 0.15% prevalence.

Interactions: During the two-week period before classes start, we assume no interaction between students and employees. We use a multi-group simulation consisting of four groups – self-quarantined students, unquarantined students, employees, and the greater Ithaca community – to model different behaviors (reflected by daily transmission rate) within and across the groups. As noted elsewhere, we assume 40% compliance with quarantine requirements amongst self-quarantining students. The transmission matrix for the self-quarantine period is summarized in Table S5.

Table S5. Inter- and intra-group transmissions per day during the self-quarantine period, based on the multi-group simulation, which use contacts from the literature, choose an infectivity calibrated to an estimate of R0, and then multiply to get transmission. Each entry gives the expected number of transmissions per day from one infected member of the row group to each of the column groups.

Group (pop. size)	Self-quarantined students	Unquarantined students	faculty / Staff	greater Ithaca community
Self-quarantined students (3748)	0.031	0.010	0	0.018
Unquarantined students (4087)	0.0087	0.053	0	0.031
Faculty / staff (10283)	0	0	0.031	0.027
Greater Ithaca community (62000)	0.0011	0.0020	0.0044	0.060

Simulation results give us that the initial prevalence among Cornell students in Ithaca immediately prior to move-in weekend is 0.17% and 0.087% for faculty and staff.

E.2. Move-in weekend and low-prevalence states. *Prevalence Estimation for Low-Prevalence States:* NY state designated a state as “high prevalence” if its daily reported number of new positive cases exceeded 10 per 100,000 population. Assuming an under-reporting factor of 10 and an average active period of 20 days, this daily new positive case threshold translates to a prevalence of 10 / 100,000 * 10 * 20 = 2%. Hence, the overall prevalence in student origins that are not designated as “high prevalence” is at most 2%. This prevalence is prior to any testing at the origin prior to departure for Cornell.

Incoming Student Population Sizes: As discussed previously, in addition to students from low prevalence states we assume that a small fraction of the students (300) from high-prevalence states that plan to live on-campus will return during the move-in weekend. Although these students will have presumably self-quarantined for 14 days elsewhere, we pessimistically assume non-compliance and consider their prevalence upon entering Ithaca to be 4%. Given it is a small population compared to students from low-prevalence states (with prevalence $< 2\%$), and the assumed under-reporting factor of 10 is large given the access to testing in low-prevalence states at the time, we assume that the overall prevalence among students returning during the move-in weekend is exactly 2%. We estimate the total number of students returning during the move-in weekend to be 10770, including 8180 undergraduate students and 2590 graduate / professional students.

Prevalence of Returning Students: Students were asked to test before departure, but this was not mandated due to a lack of test access. We assume that $\frac{2}{3}$ of the students from low-prevalence states were tested at home, using nasopharyngeal (NP) sampling (90% sensitivity). Hence, the fraction of returning students that are infectious is estimated to be $2\% * (1 - \frac{2}{3} * 90\%) = 0.8\%$. In addition, we also assume a small per-day infection probability during travel. The travel duration and the likelihood that students use public transportation (with an associated elevated daily infection probability) depends on the geographic origin of students. Weighting these probabilities by geographic origin of students, we estimate that an additional 0.1% of the returning students are infected during travel to campus. Among them, 45% are estimated to be in the Infectious state upon arrival (which can be detected with probability 90%), and 55% are estimated to be in the Exposed state upon arrival (which cannot be detected by arrival testing). Assuming arrival testing with NP sampling and 100% compliance, the fraction of returning students that are infected and not identified by arrival testing is $(0.8\% + 45\% * 0.1\%) * 10\% + 55\% * 0.1\% = 0.14\%$.

The initial prevalence estimates for the student groups combine the initial prevalence estimates from the 14-day simulation (local students and self-quarantine of high-prevalence states) and move-in weekend (low prevalence state students) to reflect the composition of each group. The initial prevalence of all the groups after arrival and immediately prior to the semester is summarized in Table S6.

Table S6. Initial prevalence estimates for modelling of Cornell Fall semester.

	UG high	UG low	GS research	GS class	FS student	FS not student	FS off	Greater Ithaca
Initial prevalence	0.156%	0.161%	0.166%	0.1628%	0.087%	0.087%	0.087%	0.08%

F. Testing Details. For asymptomatic surveillance we assume a sensitivity of 60% for PCR testing from observed self-collected anterior nares (AN) sampling, using the same test sensitivity for both pooled and individual testing. This is based on preliminary results from a validation effort at Cornell in which paired AN and nasopharyngeal (NP) swabs were collected and tested from the same individuals. Testing of AN samples identified 75% of the positives found via NP. As before, we assume a sensitivity of 90% for NP (26), that all of the positives missed by NP (10% of all positives) are also missed by AN (since these individuals would likely have low viral loads), and that an additional 25% of the 90% of the positives found by NP are missed by AN (or $0.25 \times 0.90 = 22.5\%$ of positives). This results in a sensitivity of $1 - 0.1 - 0.225 = 67.5\%$. Since AN samples are self-collected in surveillance testing, which is subject to the risk of improper sample collection, we adopt a pessimistic estimate of 0.6 for the sensitivity of surveillance tests using AN.

This estimate may be somewhat pessimistic, since some studies suggest that NP's sensitivity is higher than 90% (27), and some positives may be missed by NP sampling because of improper sampling technique (28).

On the other hand, this calculation does not explicitly account for the loss in sensitivity due to pooling. Cornell uses pools of size 5 in surveillance testing and retests the original sample when a pool tests positive. Based on existing mathematical models for pooled testing, this procedure should diagnose the same set of positives as does unpooled surveillance, unless the sample has a Ct value within $\log_2(5) = 2.3$ cycles of the limit of detection. Because SARS-CoV-2 viral loads vary by several orders of magnitude (29), the fraction of samples with a viral load in this range is small.

Inter-group Dynamics.

G. Group Details. We model the spread of COVID by splitting the campus into 8 groups and considering the interactions between groups and among themselves. We also track infections and hospitalizations in each group. The abbreviation for each group is in brackets after its name.

1. Undergraduates living in high-density housing (dorms, fraternity and sorority houses) [UG high]
2. Undergraduates living in low-density housing [UG low]
3. Graduate students primarily engaged in research [GS research]
4. Graduate and professional students primarily engaged in classroom instruction [GS class]
5. Faculty / staff working on campus who are student facing [FS student]
6. Faculty / staff working on campus who are not student facing [FS not student]
7. Faculty / staff working off campus [FS off]
8. Greater Ithaca community [Greater Ithaca]

Table S7. Group sizes for modelling of Cornell Fall semester.

Groups	UG high	UG low	GS research	GS class	FS student	FS not student	FS off	Greater Ithaca
Group size	8123	3645	4921	3598	3598	1907	4778	62000

H. Transmission. Transmission within and between each group is governed by the "transmission rate matrix." This is estimated first by estimating a rate of contacts within and between each group, and then calibrating the transmission probability per contact to a value of R0. There is a transmission rate matrix for summer of 2020 to model the pre-semester period and a transmission matrix for fall of 2020.

The term "contact" is used consistently with the literature, where a contact is defined as a two-way conversation or a physical interaction (e.g., a kiss or handshake) (30). Thus, it includes those contacts that are more brief than the CDC's definition of a close contact (6 feet or less and 15 minutes or more).

We now describe our estimation methodology for both the summer 2020 and fall 2020 matrices in an algorithmic manner.

1. Choose a nominal value of R0 in the general US population under normal circumstances. We used 2.5 as per CDC Planning Scenarios (11).

2. Choose a number of contacts per day for each age group based on the literature. We use contacts per day from (30).

3. Choose a transmission probability per contact that matches R0 to get transmissions/day as computed from (contacts / day) * (transmission / contact) for individuals, broken down by age. Based on the contact rate matrix from Step 2 and the age distribution within the US, the average number of contacts per day within the US population is 12.7. Given an R0 of 2.5 and the expected infectious period of the disease, the transmission probability is estimated to be 1.8%.

4. For each of the groups UG student, Graduate/Professional student, staff/faculty, non-Cornell Tompkins County resident, use the age distribution to calculate transmissions / day for each group, under pre-social-distancing conditions. We will subsequently adjust for social distancing.

   Transmissions per day for each group under pre-social-distancing conditions, based on the age-stratified contact rates in (30)

   - Undergraduate Student: assuming age group 15-19 in (30)
     - 17.58 contacts / day * 1.8% infectivity rate = 0.32 transmissions per day
   - Graduate Students: age group 20-29 in (30)
     - 13.57 contacts / day * 1.8% infectivity rate = 0.24 transmissions per day
   - Faculty / Staff: using the age distributions from Table S3
     - 12.9 contacts * 1.8% infectivity rate = 0.23 transmissions per day
   - Non-Cornell Greater Ithaca residents: assuming the same age demographics as reported by US census (20)
     - 12.7 contacts * 1.8% infectivity rate = 0.23 transmissions per day

5. Calculate the rate of transmission between groups using summer case count observations in Tompkins County as well as the pre-social-distancing contact rates assumed above.

   - Calibrate impact of social distancing among the Cornell summer-population (staff/faculty + summer-resident graduate/professional and UG students) and the Greater Ithaca population. Set R0 in this population to 0.75 based on the Ithaca Summer 2020 R0 argument below. This means transmissions per day is reduced 70% from our pre-social-distancing calculation (which is calibrated to R0 = 2.5).
   - Literature also suggests that younger people are less likely to abide by social distancing regulations (31). Therefore we will assume that the impact (multiplier) of social distancing is 50% less effective for students during the summer. A 70% reduction for this group becomes a 70%/1.5 = 47% reduction.
   - Using this estimate and the following additional assumptions, we can create an estimate of the summer transmission matrix. Assumptions:
     - Undergraduates and course-based graduate students all leave Ithaca over the summer.
     - 75% of research-based graduate students remain in Ithaca.
       * Transmissions per day during summer: 0.24 (1 - 0.47) = 0.127
     - All faculty/staff remained in the Ithaca area during the summer and worked remotely.
       * Transmissions per day during summer: 0.23 (1 - 0.7) = 0.069
     - The non-Cornell Ithaca community observed 70% social distancing.
       * Transmissions per day during summer: 0.23 (1 - 0.7) = 0.069
     - Breakdown of contacts by group:
       * Percent of contacts with outside community. From Figure 2A in (30), about 60% of contacts are from home, work, school, or multiple. About 20% are leisure. We will assume that social distancing scaled down transmissions proportionately, and will model 60% of transmissions for faculty/staff as Cornell-related. For faculty/staff Cornell transmissions, the majority of the contacts are within their own group (student facing, not student facing, off campus).

* Graduate students will have 75% of contacts, and thus transmissions, be Cornell-related. About 25% of these Cornell contacts are with faculty/staff and all others are with grad students. The majority of the contact with faculty/staff is with people that will be on campus and student-facing in the fall.

– Symmetry condition for daily transmissions: The expected daily transmissions between group 1 and group 2 is the expected daily transmissions between group 2 and group 1. Therefore, selecting the daily transmission rate per person in group 1 with group 2 determines the daily transmission rate of someone in group 2 with group 1.

6. This results in the summer transmission rate matrix in Table S8. The overall average transmission rate per day (within the Cornell community) for summer is 0.0828.

Table S8. Summer 2020 transmission rate matrix for Cornell.

Groups	GS research	FS student	FS not student	FS off	Greater Ithaca	Expected transmissions per day
GS research	0.072	0.021	0.0009	0.0036	0.0324	0.127
FS student	0.0169	0.018	0.0028	0.0054	0.029	0.071
FS not student	0.0013	0.0051	0.033	0.0036	0.029	0.071
FS off	0.0020	0.0041	0.0015	0.033	0.029	0.068
Greater Ithaca	0.0014	0.0016	0.00087	0.0021	0.064	0.069

7. To derive the transmission matrix for Fall 2020, we assume that the pairwise rates of interaction between grad students, faculty/staff and the Ithaca community remain the same as during the summer, but there will be an increase in overall transmission due to an influx of students arriving to campus.

- Younger people are less likely to wear masks and socially distance (31). We assume that students (undergraduates, graduate students (course-based)) reduce their pre-social-distancing transmissions by 30%, about half as effective social distancing as in Ithaca during the summer. This is more pessimistic than our previous assumption regarding graduate research students who reduced their transmissions by 47%. We do not assume an increase in transmissions per day of graduate research students with faculty/staff or the Ithaca community.
- Undergraduates (off campus): Edmunds 2006 (32) surveys undergraduate students and finds that 15.2% of their contacts are with people over the age of 30. This represents the percent of their contacts with faculty/staff and the Ithaca community. We reduce this number to 10% to reflect the reduced staff on campus. Almost all of these contacts are with student-facing staff and there is some contact with the Ithaca community.
- Undergraduates (high-density housing) have more transmissions per day with other people in high-density housing, half the transmissions per day with the Ithaca community, and the same transmissions to faculty/staff and grad students as undergraduates (off campus).
- Graduate students (course-based) have the same transmissions per day to graduate students (research), faculty/staff, and Ithaca as undergraduates (off campus). Inter-group transmissions are selected to approximate expected transmissions per day for the group.
- Grad student (research): we assume 100% of graduate research students are in Ithaca in the fall semester, while this number is assumed to be 75% during the summer.
- All rates between grad student (research), faculty/staff and Ithaca community remain the same as in the summer transition matrix.

8. This leads to the Fall 2020 transmission rate matrix in Table S9. The average transmission rate per day within the Cornell community is 0.198.

Table S9. Fall 2020 transmission rate matrix for Cornell.

Groups	UG high	UG low	GS research	GS class	FS student	FS not student	FS off	Greater Ithaca
UG high	0.22	0.072	0.0018	0.0018	0.018	0.0009	0.0009	0.0018
UG low	0.061	0.15	0.0018	0.0018	0.018	0.0009	0.0009	0.0036
GS research	0.0034	0.0039	0.072	0.0018	0.021	0.0009	0.0036	0.033
GS class	0.0025	0.0031	0.0013	0.16	0.018	0.0009	0.0009	0.0036
FS student	0.035	0.040	0.022	0.024	0.018	0.0028	0.0054	0.029
FS not student	0.0033	0.0038	0.0018	0.0023	0.0051	0.033	0.0036	0.029
FS off	0.0013	0.0016	0.0028	0.0009	0.0041	0.0015	0.033	0.029
Greater Ithaca	0.0002	0.00047	0.0019	0.00029	0.0016	0.00087	0.0021	0.064

Ithaca Summer 2020 R0 Case counts in Tompkins County in the summer of 2020 are consistent with R0 < 1 among the non-Cornell Tompkins County and summer-resident Cornell population. However, the R0 was large enough that importing

new cases created a not insubstantial number of additional cases. For the purposes of estimating the R0 of the non-Cornell community, we focus on July 2020 data.

First, according to the Tompkins County Health Department (TCHD), the number of new cases per day rose at the beginning of July when prevalence nationwide rose, but gradually declined after. If R0 were bigger than 1 in Tompkins County, then we would expect that new cases would grow exponentially. The fact that this did not happen suggests that R0 < 1.

Second, the TCHD reports that 16 out of 31 cases between July 1 and July 21 had relevant travel to a high-prevalence region. Let us make the following assumptions:

- Assume reporting bias is the same for both individuals infected locally and infected due to travel.
- Assume that all of these cases between July 1 and July 21 resulted from clusters initiated by external travel that happened in July, predominantly July 4, and not from clusters that were present in Tompkins County before July. This is based on the observation that prevalence in June in Tompkins County was very low. Also, if one assumes that some local July cases began due to pre-existing clusters then this will cause our R0 estimate to decrease further.
- Let us momentarily assume that all clusters initiated by July travel concluded by July 21. This assumption is too optimistic, and will create an R0 estimate that is too low — we will adjust for this in a moment.

In general, in a large fully susceptible population with $R0 < 1$, each new case creates a cluster that infects $1 + R0 + (R0)^2 + (R0)^3 + \ldots = 1/(1 - R0)$ individuals, including the original case. (This ignores the effect of immunity and is accurate for R0 sufficiently below 1.)

Then, under these assumptions, to find R0 in Tompkins County in July, we need to find a number such that $16/(1-R0) = 31$. Solving for R0 we get $R0 = 1 - (16/31) = 0.48$.

Finally, our third assumption above was too optimistic. In fact, some clusters that started in July due to known travel likely still had not finished infecting new people. In light of this, we increase our estimate of R0 to 0.75.

I. Virtual Instruction. This section looks at the scenario of *virtual instruction*, where research-based graduate students are on campus and subject to mandatory testing and asymptomatic screening and other students are asked not to return. In this scenario, some of these students choose to return to Ithaca despite this request. Cornell has reduced ability to enforce behavior changes and regular asymptomatic screening as compared with the residential instruction setting.

This section describes the methodology for selecting parameters for this virtual instruction scenario. In addition to the change in undergraduate and class-focused graduate student test compliance, which reflects Cornell's reduced ability to enforce behavior changes among the returning undergraduate population, two sets of additional parameters are changed relative to the Cornell re-open scenario: the group sizes (Table S7) and the transmission rate matrix (Table S9).

1. Group Sizes

   Table S10 gives the population size for each group for virtual instruction. We assume that the last three columns — Faculty/Staff not student-facing, Faculty/Staff off-campus, and Greater Ithaca — are independent of the policy change since the people in those groups are very likely to obey the same routines regardless of the scenario.

Table S10. Group sizes for virtual instruction scenario.

Groups	UG high	UG low	GS research	GS class	FS student	FS not student	FS off	Greater Ithaca
Group size	0	3468	1594	1434	3598	1907	4778	62000

   To estimate the population sizes for the student groups, we used results from a survey sent out on May 29, 2020 to all students enrolled at the time, while attending to two concerns:

   (a) Not all of the students who received the survey responded.
   (b) The survey result does not include students who would enroll in the fall of 2020 for the first time, namely rising undergraduate freshmen and new graduate students.

   For the first concern, since 71% of the undergraduates and 48% of the graduates responded, we assume these percentages generalize to the whole population. For the second concern, we will explain group by group how we handle it.

   - Undergraduate students:
     - For the UG high-density housing ("UG high") group: we set the group size to be 0, since on-campus dorms would be closed.
     - For the UG low-density housing ("UG low") group: the number is calculated from 11186*0.31=3486 where 11186 is the number of undergrads surveyed and 31% is the percentage who responded "very likely" to return for a virtual semester. The number of survey recipients, 11186, does not include any of the incoming first year students. Using this number, we are implicitly assuming that no freshman students come to Ithaca under a virtual instruction scenario, which is conservative in the sense that it under-estimates unsurveilled students in this scenario.

- Graduate students:
  - There are two graduate student groups, GS class and GS research. In the residential scenario, these groups have population sizes 4921 and 3645, respectively.
  - From the May 29 survey results, we estimate that 53% of the graduate student population would return under a virtual instruction scenario. We assume this percentage applies evenly across both class-based and research-based graduate students.
  - We assume that 25% of research graduate students are first years, and 50% of class-based graduate students are first years. We assume that non-first-year students in each group are subject to the 53% return percentage, from which we obtain $4921 \times 0.5 \times 0.53 = 1304$, and $3645 \times 0.75 \times 0.53 = 1449$, corresponding to the number of non-first-year students who return to Ithaca from each of the GS class and GS research groups.
  - For the first-year graduate students in each group, we assume that the 53% likely-to-return proportion is reduced by a further 90% in the case of class-based students, and 70% in the case of research-based grad students. This gives a total of $4921 \times 0.53 \times 0.5 \times 0.1 = 130$ and $3645 \times 0.25 \times 0.53 \times 0.3 = 149$ first year graduate students returning to Ithaca in each of the groups.
  - Combining the above, we get 1434 class-based graduate students and 1594 research-based graduate students.
- Faculty and Staff

  As we stated above, we assume faculty and staff behaviors are somewhat independent of the scenarios. Thus, we keep the faculty populations the same as an in-person semester in each group.

2. Transmission Rate Matrix

   The transmission rates for virtual instruction are based on the transmission rates for residential instruction with some adjustments. As a reminder, transmission rate = contacts / day * 1.8% infectivity rate, and we assume that the interaction between faculty/staff within themselves and with the Greater Ithaca community does not depend on scenarios. The main idea for estimating transmission rates for virtual instruction is that class-based students would interact less with faculty and staff, but more with the Greater Ithaca community. Student interactions among themselves depend on their compliance with the behavioral compact (e.g., mask-wearing and social distancing) and housing density in Collegetown. We explain each of the transmission rates we have re-calculated below.

- UG high
  - Since we assume no one in "UG high" will return, there is no transmission from this group to others.
- UG low / GS class within-group
  - The virtual scenario has two competing effects: reduced density of transmissions due to fewer people on campus, and potential increase in transmissions due to Cornell's reduced ability to enforce mask wearing, social gathering restrictions, and abundant asymptomatic testing.
  - First, we discuss the effect of social gathering and mask wearing. In the residential instruction scenario, we assumed that Cornell's ability to legally mandate mask wearing and social gathering restrictions with a behavioral compact resulted in a 30% reduction in transmission between pre-social-distancing periods and a residential fall semester. Under virtual instruction, since Cornell will not be able to enforce a behavioral compact, we assume that this reduction in transmission (between the summer and a virtual fall semester) will be less than between the summer and residential instruction. While one might imagine that there would be no reduction in transmission between the summer and a virtual instruction fall given Cornell's reduced ability to enforce a behavioral compact, we optimistically assume a 15% reduction. This has the effect of increasing the within-group transmission rates of "UG low" and "GS class" by a factor of (1-15%)/(1-30%) from the residential setting.
  - Second, Section 3.1 and Figure 4 of (33) suggest that the mortality rate of infectious diseases rises with population density up until population density reaches 200 people per square mile and then levels off. Below, we estimate that virtual instruction reduces the population density to roughly 2000 / square mile from roughly 6000 / square mile under residential instruction. Although the literature thus suggests that there will be no reduction in transmissions due to virtual instruction relative to residential instruction, we conservatively assume that virtual instruction will result in a reduction of transmissions by 20%.
  - Population-density calculation: For the people who live in Ithaca, according to the percentage in Section A5, roughly 30% of the juniors, seniors and class-based graduate students who live in Collegetown are returning this fall. Moreover, we estimate that roughly 20% of Collegetown residents are not undergraduates and not class-based graduate students. Thus, in total the density in Collegetown is around (0.8*0.3+0.2*0.5)=0.34 of Collegetown residents are returning. Since the City of Ithaca has a living density of 5893 people per square mile, Collegetown has 5893*0.34=2004 people per square mile for the virtual instruction scenario.
  - Combining the two effects described above, we multiply the residential within-group transmission rate for "UG low" and "GS class" by a factor of (1-15%)/(1-30%) * (1-20%) = 0.9712

- UG low / GS class with faculty, staff and graduate students:
  - "UG low" and "GS class" will have much less interaction with "FS student" and "FS not student" since they do not need to see any professors in person. Thus, we assume the transmissions from any "UG low" and "GS class" person to on-campus faculty will drop to minimal to be the same as transmissions to any off-campus faculty.
- UG low / GS class with Greater Ithaca:
  - A virtual semester that shuts down the campus including the dining halls would increase undergraduate interaction with Greater Ithaca for reasons like groceries and other necessary activities. However, "UG low" and "GS class" are unlikely to leave the Collegetown area very frequently. Therefore, a number larger than the transmission rate for UG low / GS class with Greater Ithaca in the residential instruction scenario but less than that of GS research would be a reasonable estimate. Thus, we set the transmission rate for UG low / GS class with Greater Ithaca to be a little over half of that of GS research with Greater Ithaca, the figures of which do not change from scenario to scenario.

In summary, Table S11 gives the virtual instruction transmission matrix.

Table S11. Virtual instruction transmission rate matrix for Cornell.

Groups	UG high	UG low	GS research	GS class	FS student	FS not student	FS off	Greater Ithaca
UG high	0	0	0	0	0	0	0	0
UG low	0	0.20	0.0018	0.0018	0.0009	0.0009	0.0009	0.0018
GS research	0	0.0039	0.072	0.0018	0.021	0.0009	0.0036	0.033
GS class	0	0.0043	0.0020	0.16	0.0009	0.0009	0.0009	0.0018
FS student	0	0.00087	0.0095	0.00036	0.018	0.0028	0.0054	0.029
FS not student	0	0.0017	0.00075	0.00068	0.0051	0.033	0.0036	0.029
FS off	0	0.00066	0.0012	0.00028	0.0040	0.0015	0.033	0.029
Greater Ithaca	0	0.0001	0.0008	0.0004	0.0016	0.00087	0.0021	0.064

J. Matrix Input for Simulation. We have previously described how we estimated transmission matrices for the Fall semester (Tables S9 and S11). These matrices represent the average number of new infections per day in the column group from each free and infectious person in the row group. Unfortunately, our code is not structured to directly take the transmission matrix as an input.

Instead, it takes the so-called "interaction matrix" as an input, where the mean number of new infections in group i from group j in a day is given by

$$p * \texttt{free\_susceptible}[i] * \texttt{interactions}[i,j] * \texttt{free\_infectious}[j]/\texttt{free\_total}[j]. \quad [1]$$

Here, p is the probability of transmission per interaction, $\texttt{interactions}[i,j]$ is the value of the matrix inputted to the simulation at row i and column j, $\texttt{free\_susceptible}[i]$ is the number of free and susceptible individuals in group i, $\texttt{free\_infectious}[j]$ is the number of free and infectious individuals in group j, $\texttt{free\_total}[j]$ is the total number of free individuals in group j.

Note that $\texttt{interactions}[i,j]$ was intended to represent the number of contacts within group j by a single person in group i on a single day and $\texttt{free\_infectious}[j]/\texttt{free\_total}[j]$ is the fraction of the free population in j that is infectious. Thus, the expected number of contacts that a free susceptible person in group i would have with a free and infectious person in group j would be $\texttt{interactions}[i,j] * \texttt{free\_infectious}[j]/\texttt{free\_total}[j]$. We then multiply by the number of free susceptible individuals in group i and the probability of transmission upon contact to get the total number of contacts with infectious people in group j by free and susceptible people in group i. This recovers Equation 1.

To convert the transmissions matrix (Tables S9 and S11) to the interaction matrix used as an input to our simulation, we will count in two ways the number of interactions between infectious people in group j and susceptible people in group i, and set them equal to each other.

First, consider the infectious people in group j and count their interactions with people in group i. There are a total of $\texttt{free\_infectious}[j] \cdot \texttt{transmissions}[j,i]$ transmissions from group j to group i. This implies $\texttt{free\_infectious}[j] \cdot \texttt{transmissions}[j,i]/p$ total interactions with susceptible people in group i.

The second way to count the number of interactions is starting with the susceptible population in group i which has a total of $\texttt{free\_susceptible}[i] \cdot \texttt{interactions}[i,j]$ contacts with members of group j. Of these contacts the following fraction are with infectious people in group j, $\texttt{free\_infectious}[j]/\texttt{free\_total}[j]$. Therefore, there are a total of $\texttt{free\_susceptible}[i] \cdot \texttt{interactions}[i,j] \cdot \texttt{free\_infectious}[j]/\texttt{free\_total}[j]$ interactions between infectious members of group j and susceptible members of group i.

Setting these two expressions equal to each other and cancelling $\texttt{free\_infectious}[j]$ gives us $\texttt{transmissions}[j,i] = p \cdot \texttt{interactions}[i,j] \cdot \texttt{free\_susceptible}[i]/\texttt{free\_total}[j]$. Given low prevalence, we then assume that the susceptible and total free populations of each group are approximately their respective population sizes. This yields $\texttt{transmissions}[j,i] = p \cdot \texttt{interactions}[i,j] \cdot \texttt{population}[i]/\texttt{population}[j]$.

2. Model Calibration

This section describes the *retrospective* parameter estimation and model calibration *after* for the fall 2020 and spring 2021 semesters. Sections A and B describe model calibrations for students and employees in the fall 2020 semester, respectively. Sections C and D describe model calibrations for students and employees in the spring 2021 semester, respectively.

Parameter estimation relies on data from the following sources:

- Aggregated de-identified positive-case, testing, and contact-tracing data collected during the semester and stored along with student life, housing, and employee data in a HIPAA-compliant database. This data was collected by Cornell to meet an urgent public health need while fighting the pandemic. This data was then aggregated and shared by the institution with the authors for research purposes. A determination was made by Cornell's Institutional Review Board (IRB) that the use of this previously collected aggregated data for research does not constitute human subjects research.

- Data in a publicly available report pursuant to the urgent public health need presented by the pandemic (4).

The data sources for all parameters are summarized in Table S12.

Table S12. Data sources of parameter estimates/calibration for the fall 2020 and spring 2021 semesters. "V" indicates that the data is obtained from the HIPAA-compliant database; "P" indicates that data is obtained from the publicly available report.

	Parameter name	Source for the fall 2020 calibration	Source for the spring 2021 calibration
Student calibration	Population size	P	V
	Observed trajectories	P	V
	Arrival schedule	V	not used
	Testing frequency	V	V
	Test compliance	V	V
	Outside infection rate	P	V
	Contact matrix	P	V
	Contact tracing effectiveness parameters	V, P	V
	Initial prevalence	V	V
Employee calibration	Observed trajectory	P	V
	Testing frequency	V	V
	Outside infection rate	V	V
	Contact tracing effectiveness parameters	V	V

A. Model Calibration for Students in the Fall 2020 semester. We use a multi-group dynamic population simulation model for the student population, which consists of three subgroups:

- Group 1: undergraduates, with Greek-life or varsity athletics affiliation;

- Group 2: undergraduates, with no Greek-life or varsity athletics affiliation;

- Group 3: graduate or professional students.

We employ this population breakdown because we observe substantial differences in infections and contacts for these specific subgroups. We set August 16, 2020 - November 24, 2020 to be the time period for our calibration, as the majority of undergraduates left the greater Ithaca area at the time of the Thanksgiving holiday. We divide the time horizon into two non-overlapping periods: the pre-semester period (8/16/2020 - 9/2/2020) and the in-semester period (9/3/2020 - 11/24/2020).

Here we describe the parameters estimated directly from fall 2020 data.

Population Size We use students' degree program information, Greek-life affiliation and varsity athlete rosters, and daily check-in data to divide students residing in Ithaca into 3 subgroups, obtaining the population sizes given by Table S13.

Table S13. Sizes of the three student groups used in fall 2020 student calibration and projection.

Group	Population size
1 (UG with Greek-life or varsity athletics affiliation)	3533
2 (Other UG)	8434
3 (Graduate and professional students)	6202

Arrival Schedule Arriving schedules for groups 1, 2 and 3 are determined based on the arrival dates indicated by students in their Fall semester checklist, and the move-in schedule for students living on campus.

Testing Frequency The model does not track individuals and their test schedules. Rather, each member of a population is assumed to test on a given day with a given probability.

- Pre-semester period:
  - Groups 1-2: We divide the total number of non-arrival tests performed (3255) during the period by the total number of person days during the pre-semester period (127466) to estimate the testing frequency for the undergraduate students in the pre-semester period, to get 0.0255 per day per person, i.e., each person has one test on average every 39 days.
  - Group 3: 0.
- In-semester period:
  - Groups 1 and 2: 2/7 per day, corresponding to being tested 2x / week.
  - Group 3: 1/7 per day, corresponding to being tested 1x / week.

The testing frequency during the in-semester period is consistent with the testing frequency for students assumed in the main simulation model.

Test Compliance We estimate student test compliance to be 97.4%. This value is calculated based on the fraction of scheduled student surveillance tests completed over the course of the fall semester (including both on-time tests and those tests that were delayed but completed).

Outside Infection Rate We consider a positive student case to be an outside infection if they satisfied both of the following conditions:

- they did not test positive in an adaptive test, nor were they in the contact trace of other positive cases;
- they had recent travel history;
- they are not classified as an "arrival positive" case.

Table S14 summarizes the number of outside infections in each group during the semester and the corresponding outside infection rate, which is the number of outside infections divided by (population size of the group × time horizon in days).

Table S14. The number of outside infections in each group during the fall 2020 semester and the corresponding outside infection rate.

Group	Outside infection case count	Outside infection rate (per person, per day)
1	5	1.42E-5
2	6	7.11E-6
3	4	6.45E-6

Note that the period considered does not include the post-Thanksgiving period. During the post-Thanksgiving period, graduate students tested positive at a higher rate due to travel.

Contact Matrix We define the *daily transmission matrix* T such that the value $T(i, j)$ gives, for each infectious non-isolated non-quarantined positive in group i, the expected number of additional positives created in group j on a given day. It is difficult to estimate the daily transmission matrix directly from data because we do not observe for how many days an individual was positive. Instead, we aim to estimate the *contact matrix* M. The value $M(i, j)$ in the contact matrix is the expected number of positive cases that an infectious individual in group i creates in group j over the course of his or her infection. We then assume that the average length of time an infectious individual in a given group spends circulating (i.e., not isolated or quarantined) during the fall semester does not depend on their group. Under this assumption, M is proportional to T. Below, in our calibration to observed infection counts during the fall semester, we estimate the proportionality constant, α, and then $T = \alpha M$.

To estimate the contact matrix, we make the following additional assumptions:

- Each case identified through adaptive testing was generated by a source case in the same group.
- All positives in the student population created by an infectious student are identified as a close contact of that student (even if they were originally identified and tested because of surveillance testing, symptoms, or adaptive testing).

We first classify the student positives in the in-person semester (184 cases between 8/16/2020 and 11/24/2020) into source cases and secondary cases. Here, "secondary cases" include those identified via contact tracing or adaptive testing. The remaining cases, identified through surveillance testing, symptomatic self-reporting, arrival testing, or testing positive after returning from travel, are classified as source cases.

Based on these assumptions, we estimate the contact matrix using the following methodology:

- We begin by identifying all positive cases in each group i. Let this be $N(i)$.
- For each group j, we count the number of positives in group j that were identified as a close contact of a person in group i. Let this be $L(i,j)$. A positive who is a close contact of people in multiple groups is counted proportionally to the groups of the people that identified them as contacts. For example, a positive person in group 2 who is identified as a close contact of one person in group 1 and two people in group 2 would contribute $\frac{1}{3}$ to $L(1,2)$ and $\frac{2}{3}$ to $L(2,2)$.
- For each group j, we additionally count the number of positive people that were identified through adaptive testing but were not identified as a close contact. In an abuse of notation, let this be $L(j)$.
- The value of $M(i,j)$ is then $(L(i,j) + \mathbb{1}\{j = i\}L(j))/N(i)$.

We use identified contacts in producing these estimates. When contacts are not identified, this could distort the estimates. Assuming that contact tracing is equally effective for all source groups and "destination" groups, thus resulting in a constant fraction of contacts missed, the fact that we only use the matrix up to a multiplicative proportionality constant should ensure that the resulting error is controlled. The resulting contact matrix M is shown in Table S15.

Table S15. The contact matrix M for the fall 2020 semester. Cell $M(i,j)$ is the average number of positive cases in group j that an infectious individual in group i creates over the course of his or her own infection.

Source cases group (counts)	Average # positive contacts in Group 1	Average # positive contacts in Group 2	Average # positive contacts in Group 3
Group 1 (125)	$(81+11)/125 = 0.736$	$3.5/125 = 0.028$	0
Group 2 (44)	$1/44 = 0.023$	$(4.5+2)/44 = 0.148$	$1/44 = 0.023$
Group 3 (15)	0	0	$1/15 = 0.067$

Contact Tracing Effectiveness Parameters Our stochastic compartmental model does not track individuals. Instead, it tracks the number of individuals in a collection of different states. This makes it difficult to simulate contact tracing at an individual level. Instead, our model relies on the following two parameters:

1. `cases_isolated_per_cluster`: The number of positive cases isolated for each contact trace (which models both contact tracing and adaptive testing) initiated by a self-reporting symptomatic individual or one identified through surveillance testing.
2. `cases_quarantined_per_cluster`: The number of negative cases quarantined for each contact trace initiated by a self-reporting symptomatic individual or one identified through surveillance testing.

In the simulation, all infected individuals are considered to be isolated, even if we would not have known in reality that the individual was positive and would have initially placed them into quarantine.

`cases_isolated_per_cluster` corresponds to the average number of secondary cases identified through an initiated trace from a positive case in real life. This can be estimated from the ratio of the number of secondary cases (105) to the number of source cases (79), which gives 1.329. In comparison, the effective `cases_isolated_per_cluster` assumed in the projections for the fall is $0.85/2 = 0.43$, which is approximately $1/3$ of the calibrated value. This in part explains the conservative projections for the fall.

`cases_quarantine_per_cluster` can be estimated from the ratio of the number of negative cases identified in contact tracing (378) to the number of sources cases (79), which gives 4.785. Individuals identified in adaptive testing are not quarantined.

In summary, our estimated parameters are

- `cases_isolated_per_cluster`: 1.329;
- `cases_quarantined_per_cluster`: 4.785.

Initial Prevalence The model relies on an initial prevalence of free and infectious cases. The calibrated values are

- Group 1: 5.77 average initial cases;
- Group 2: 3.37 average initial cases;
- Group 3: 0.

For groups 1 and 2, we consider the initial free and infectious cases at the beginning of the simulation to be the union of those imported positive cases missed by the arrival test, and those secondary cases infected by arrival positives due to the lag between arrival and taking arrival tests.

We determine the arrival positives based on whether the positive students tested positive on their first test. This produces 11 cases, out of which 5 cases are in group 1, and 6 are in group 2.

Then, we estimate the number of imported positive cases missed by the arrival tests based on the number of arrival positives, the sensitivity of the arrival testing (assumed to be 90% for nasopharyngeal sampling PCR test) for individuals in the post-exposure pre-convalescence infectious period and the probability that an infected person is in the exposed state and thus

not identifiable by a PCR test (estimated to be 0.2 based on state occupancy times in our model). Hence, for any positive case arriving in Ithaca, the probability that it is not identified by the arrival test is $P(\text{exposed state}) + P(\text{not in exposed state}) \cdot (1 - \text{sensitivity}) = 0.2 + 0.8 \times 0.1 = 0.28$. This implies that for every arrival positive case, there are $0.28/(1 - 0.28) = 0.39$ free and infected cases acting as the initial cases in the simulation. In more detail, (# observed cases) $= (1 - 0.28) \cdot$ (# cases), and (# free and infectious cases) $= 0.28 \cdot$ (# cases), so (# free and infectious cases) $= 0.28 \cdot$ (# observed cases)$/(1 - 0.28) =$ 0.39(# observed cases).

Thus, the number of free and infectious cases created immediately are:

- Group 1: $0.39 \times 5 = 1.95$;
- Group 2: $0.39 \times 6 = 2.34$.

Third, we estimate the number of secondary cases resulting from the arrival positives, due to the fact that students did not take their arrival test right upon arrival and hence could infect other students during the testing delay. This is obtained based on the contact matrix M (as described above), assuming that each arrival positive in group j infects $M(i, j)$ individuals in group i.

We summarize the number of secondary cases in each group below:

- Group 1: $5 \times 92/125 + 6 \times 1/44 = 3.82$;
- Group 2: $6 \times 6.5/44 + 5 \times 3.5/125 = 1.03$.

In summary, the average number of initial cases in groups 1 and 2 are given below:

- Group 1: $1.95 + 3.82 = 5.77$;
- Group 2: $2.34 + 1.03 = 3.37$.

For group 3, since we did not observe its first positive case after 8/16/2020 until 9/12/2020, we set the initial prevalence to be zero.

Calibration Results We calibrate our model's projected infections to the actual trajectory within 3 subgroups from 8/16/2020 - 11/24/2020, as shown below. The total number of positive cases observed within the time period is described below and the trajectories are described in Figure S2.

- Group 1: 120, excluding 5 arrival positives excluded;
- Group 2: 38, excluding 6 arrival positives excluded;
- Group 3: 15.

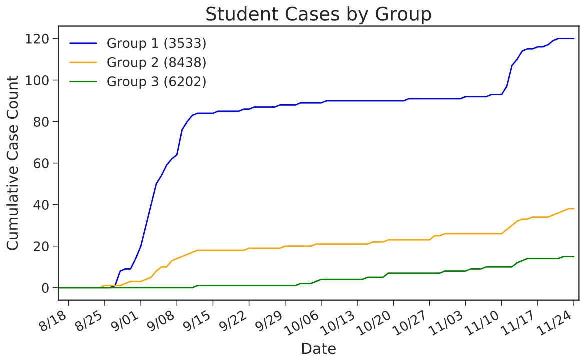

Fig. S2. Observed infections (excluding arrival positives) among students during the fall 2020 semester, shown for each of the three student groups.

Here we tune the parameter α in the simulation, i.e., the proportionality constant described in the contact matrix section above. For each value of α, we compute the mean squared error of the simulated results described as follows:

- Let $\text{sim}(t, i, j)$ denote the number of infections on day t in replication i for group j according to the simulation.
- Let $\text{actual}(t, j)$ denote the number of infections observed on day t for group j.

- Then, the error score associated with α is given by

$$\mathrm{err}(\alpha) = \sum_{j\in\{1,2,3\}} \sum_{t=1}^{T} \left(\frac{1}{N} \sum_{i=1}^{N} \mathrm{sim}(t,i,j) - \mathrm{actual}(t,j) \right)^2 /T,$$

where N is the number of simulation replications and T is the simulation horizon.

Figure S3 shows the log root mean-squared error of our model predictions versus α. We see that when $\alpha = 0.525$, the lowest error score is obtained.

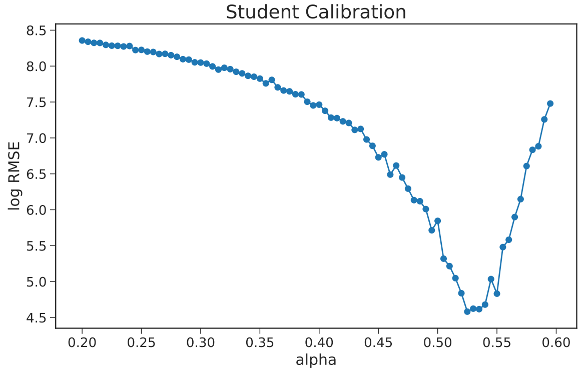

Fig. S3. For fall 2020, the log of the root mean-squared error (RMSE) of projected student infections versus α, the proportionality constant that multiplies the contact matrix to obtain the daily transmission rate.

Figure S4 shows the simulated trajectories (25 in each group) when $\alpha = 0.525$, in comparison to the actual trajectories for students cases in different subgroups.

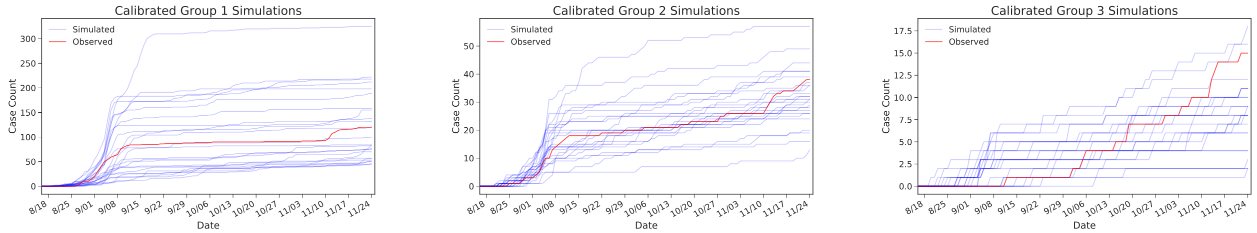

Fig. S4. Observed infection trajectories for each student group, over the course of the fall 2020 semester, plotted along with stochastic sample trajectories from the simulation under the estimated parameters.

B. Model Calibration for Employees in the Fall 2020 semester. To calibrate our model for faculty and staff we use a single-group simulation model consisting of all faculty and staff with population size 10283, and include all infections that occurred between August 16, 2020 and January 10, 2021.

We have access to less detailed data about employees compared with students. In particular, we do not have access to contact tracing data for the fall semester. Understanding the difficulties of estimating inter-group transmission rates given a lack of contact tracing data, we elect not to partition the employee group (partitions considered included those based on county of residence or job type).

Observing rising infection counts among faculty and staff after Thanksgiving, we decide to include December and early January in the period of interest. We divide the time horizon into two non-overlapping periods: the pre-semester period (8/16/2020 - 9/2/2020) and the period after (9/3/2020 - 1/10/2021).

In place of contact tracing data, we leverage "`cluster_ids`" that were generated from manual review of employee cases. An employee case is assigned a `cluster_id` if that case is believed to be linked to at least one other case at Cornell, with all linked cases being assigned the same `cluster_id`. The use of the term "cluster" is perhaps misleading, since even pairs of positive cases that are linked through off-campus contact (often, two employees living together) are given a cluster id. These cluster ids allow us to estimate outside infections and `cases_isolated_per_cluster`. In most cases, evidence suggests that those individuals without a cluster id were infected through non-Cornell interaction. This evidence, when it exists, consists of information obtained from contact tracing (e.g., that there is known close contact with a positive non-Cornell-affiliated individual) or the lack of other cases at Cornell at similar times in parts of the employee population that would interact with the positive individual on campus.

Here we describe the parameters estimated directly from fall 2020 data in the model calibration for employee group.

Testing Frequency 0 during pre-semester period; 0.098 per day after. (The latter value is an average across those tested once per week and those tested once every two weeks.)

Outside Infection Rate We classify a case as an "outside infection" if they did not contract the virus through interactions with other Cornell cases. (Transmission from one Cornell case to another is not considered an outside infection, even if the transmission occurred away from Cornell's campus.) To estimate the outside infection rate for Cornell employees (faculty/staff), we assume that

- Cases without `cluster_id`s are outside infections;
- Exactly one case in each identified cluster is an outside infection, while the remaining cases in the cluster are not outside infections.

Based on these two assumptions, we have a simple formula for calculating the number of outside infections: (# cases without a `cluster_id`) + (# clusters). Below we summarize the outside infection counts during the specified time period.

- 246 employee cases in total in the date range 8/16/2020 - 1/10/2021; 159 without a `cluster_id`; 25 distinct clusters.
- # outside infections = 159 + 25=184 (74.8%); # non-outside infections = 62 (25.2%).
- Average Daily outside infection rate: 184 / (# faculty and staff $\times$ 148 days) = 1.21E-4, i.e., in a population of 10,000 people, we should expect to see 1.2 infections per day due to travel and interaction with the outside community.

To address the rising trend in the number of employee cases, in the simulation we used a time-varying outside infection rate (measured in infections per day), which is computed by weekly faculty/staff outside infections divided by (# faculty and staff $\times$ 7 days). We assume that the outside infection associated with each `cluster_id` occurred during the week of the first identified case associated with that `cluster_id`.

Contact Tracing Effectiveness Parameters Recall that our simulation quantifies the effectiveness of contact tracing through a parameter, `cases_isolated_per_cluster`, which is the number of cases isolated for each cluster traced. Cluster traces are initiated by the discovery of a self-reporting symptomatic individual or by a case found via surveillance testing.

The number of positive cases isolated per contact trace is lower bounded by 0 and upper bounded by the average number of secondary positive cases per cluster. This is because it is only those cases in a cluster that can be linked through contact tracing. Here, we think of solo cases without a `cluster_id` as clusters of size 1.

To estimate this upper bound, we average (cluster size - 1) across all clusters. There are 25 identified clusters with size > 1, containing 87 cases in total. There are 159 cases without a `cluster_id`. Therefore, the average cluster size is (87 + 159) / (25 + 159) = 1.34, and avg (cluster size - 1) is 0.34.

We choose to use $0.34 \times 0.75 = 0.255$, assuming that 75% of the people found in clusters among Cornell employees were found via contact tracing or adaptive testing, with another 25% found via symptomatic self-reporting or surveillance testing. This is based, in part, on the observation that a large fraction of Cornell employee clusters are among family members and these would almost always be found via contact tracing. We assume that it is rare for positives in the Cornell community to be found via contact tracing of people who are not part of the Cornell community.

Thus, in summary, `cases_isolated_per_cluster` = 0.255.

Outcomes are insensitive to the parameter `cases_quarantined_per_cluster`, which determines the number of negative individuals quarantined, because its only effect on infections is to reduce the number of susceptible people that can be infected. Given that the fraction of the population quarantined is small, it has little effect on outcomes over several orders of magnitude. Because information about employee quarantines was unavailable, we set it to 2.5, a value close to half of the value observed for students, since employees were observed to have fewer contacts than students.

Calibration Results We calibrate our model's projected infections to the actual trajectory from 8/16/2020 - 1/10/2021, which is shown in Figure S5.

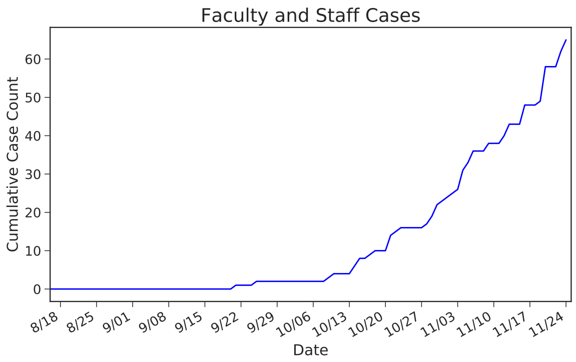

Fig. S5. Trajectory of employee infections from 8/16/2020 to 1/10/2021, the period used for fall 2020 calibration.

We then plot the log root mean-squared error (RMSE) between the observed trajectory and the average output of the simulation, versus the parameter we wish to calibrate, which is the daily transmission rate (# of other Cornell employees infected per day by a positive Cornell employee). Here, analogous to the error function used in the calibration for the student groups, the mean squared error is given by

$$\mathrm{err}(\alpha) = \sum_{t=1}^{T} \left(\frac{1}{N} \sum_{i=1}^{N} \mathrm{sim}(t,i) - \mathrm{actual}(t) \right)^2 /T,$$

where $\mathrm{sim}(t,i)$ is the number of infections on day t in replication i according to the simulation, $\mathrm{actual}(t)$ is the number of infections observed on day t, N is the number of simulation replications, and T is the simulation horizon. Note that many of these infections occurred between family members who are both Cornell employees but infected each other at home.

Figure S6 shows the log RMSE versus employee transmission rate. We see in this figure that when the daily transmission rate is 0.11, the lowest log error is obtained. Thus, according to our calibrated model, each infectious employee infects 0.11 other employees on each day they are infectious.

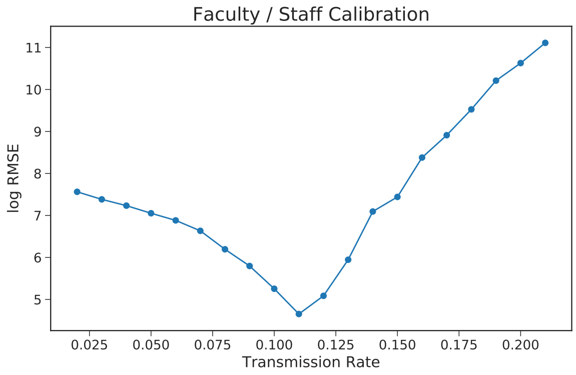

Fig. S6. For fall 2020, the log of the root mean-squared error (RMSE) of projected employee infections versus employee transmission rate.

Figure S7 shows 25 simulated trajectories when the daily transmission rate is 0.11, in comparison to the actual trajectory for faculty and staff cases. We observe that the observed case counts are reasonably well-represented by the simulation. Growth in cases during the semester is driven by an increase in outside infection rate rather than transmission within the Cornell population.

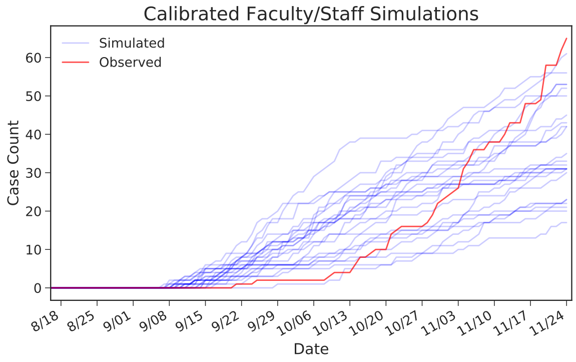

Fig. S7. Projections from our model (blue lines) using the calibrated daily transmission rate for the fall 2020 semester, compared with the observed infection trajectory (red line).

C. Model Calibration for Students in the Spring 2021 semester. We use a multi-group simulation model for the student population, which consists of four subgroups:

- Group 1: undergraduates, with Greek-life or varsity athletics affiliation;
- Group 2: undergraduates, with no Greek-life or varsity athletics affiliation;
- Group 3: students in the MBA program;

- Group 4: graduate or professional students, non MBA.

We employ this population breakdown because we observe substantial differences in infections and contacts for these specific subgroups. In particular, the population breakdown is different from that we used in the fall 2020 semester because we observe higher transmission rate and lower test compliance rate in the MBA student group. We set January 21, 2021 - May 25, 2021 to be the time period for our calibration. We divide the time horizon into two non-overlapping periods: the pre-semester arrival period (1/21/2021 - 2/7/2021) and the in-semester period (2/8/2021 - 5/25/2021).

Here we describe the parameters estimated directly from spring 2021 data. We estimate many of these parameters using the same methodology described in Section A above, for which we directly report the results.

Population Size We use the same methodology as in the fall 2020 calibration to obtain the population sizes, given by Table S16.

Table S16. Sizes of the three student groups used in the spring 2021 student calibration and projection.

Group	Population size
1 (UG with Greek-life or varsity athletics affiliation)	3329
2 (Other UG)	9033
3 (MBA students)	534
4 (Graduate and Professional Students, non MBA)	5227

Testing Frequency As is in the fall 2020 calibration, the model does not track individuals and their test schedules. Rather, each member of a population is assumed to test on a given day with a given probability. Table S17 describes the actual and scheduled testing frequencies in each student group during the spring 2021 semester. In certain student groups we observe that the actual testing frequency is higher than the scheduled testing frequency because students may seek to get additional tests even when they were not required to do so.

Table S17. The testing frequency in each group during the spring 2021 semester and the corresponding test compliance rate.

Group	Average actual testing frequency (# tests per day)	Scheduled testing frequency (# tests per day)	Ratio of actual testing frequency to scheduled testing frequency
1	0.355	3/7	0.828
2	0.285	2/7	0.998
3, on or before 3/26/2021	0.152	1/7	1.064
3, on or before 3/26/2021	0.241	2/7	0.844
4	0.148	1/7	1.036

Outside Infection Rate We use the same methodology as in the fall 2020 calibration to obtain the outside infection rate, given by Table S18.

Table S18. The number of outside infections in each group during the spring 2021 semester and the corresponding outside infection rate.

Group	Outside infection case count	Outside infection rate (per person, per day)
1	7	1.68E-5
2	3	2.66E-6
3	3	4.49E-5
4	1	1.53E-6

Contact Matrix Recall that the (i, j) entry of a contact matrix is the average number of positive cases in group j that an infectious individual in group i creates over the course of his/her infection. To compute the contact matrix for spring 2021, We use a methodology similar to that of the fall 2020 calibration but make one minor modification. In the fall 2020 calibration, we assumed that the average time an infectious individual in a given group spends circulating (i.e., not isolated or quarantined) does not depend on their group membership. For the spring 2021 semester, we instead assume that the time an infectious individual circulates for is inversely proportional to his/her test frequency. That is, the more frequently an individual is tested, the less time he/she has to generate secondary infections. This assumption is necessitated due to the heterogeneity in testing frequencies across different groups in spring, and the fact that unlike in fall 2020, a significant fraction of the cases occurred among the graduate and professional students (Group 3 and Group 4) in spring 2021.

We outline the steps of adjusting for the different testing frequencies in different groups when computing the contact matrix for spring 2021 calibration. Such adjustment would have minimal effect on the fall 2020 contact matrix, because infections were concentrated in the undergraduate population (Group 1 and Group 2) who were tested 2x/week.

- Under the assumption above, those tested 3x/week had 1/3 less circulation time on average than those tested 2x/week, while those tested 1x/week had twice circulation time on average as those tested 2x/week.
- Using 2x/week testing as a baseline, we adjust the number of positive contacts of cases in groups tested 3x or 1x/week so that they reflect the number of positive contacts over the same circulation time as those tested 2x/week.

In particular, we multiply the number of positive contacts by 1.5 for source cases in Group 1 (tested 3x/week throughout the semester) and by 0.5 for source cases in Group 4 (tested 1x/week throughout the semester). Students in Group 3 were tested 1x/week on or before 3/26/2021 and 2x/week after 3/26/2021, so the number of their positive contacts is scaled by 0.5 in the first period and unscaled in the second period. The resulting adjusted contact matrix M for the spring 2021 semester is shown in Table S19.

Table S19. The (adjusted) contact matrix M for the spring 2021 semester. Cell $M(i,j)$ is the average number of positive cases in group j that an infectious individual in group i creates over the course of his or her own infection.

Source cases group (counts)	Average # positive contacts in Group 1	Average # positive contacts in Group 2	Average # positive contacts in Group 3	Average # positive contacts in Group 4
Group 1 (194)	0.695	0.197	0	0.023
Group 2 (244)	0.074	0.285	0	0.004
Group 3 (66)	0	0	0.394	0.023
Group 4 (56)	0	0.018	0	0.076

Contact Tracing Effectiveness Parameters We use the same methodology as in the fall 2020 calibration to estimate the contact tracing effectiveness parameters, given below:

- `cases_isolated_per_cluster`: 0.854;
- `cases_quarantined_per_cluster`: 3.083.

Initial Prevalence We first summarize the estimated average number of initial cases in each student group:

- Group 1: 12.9 average initial cases;
- Group 2: 55.5 average initial cases;
- Group 3: 2.3 average initial cases;
- Group 4: 20.3 average initial cases.

These initial cases are assumed to spread uniformly over the pre-semester arrival period in our simulation.

Below we describe in detail how we derive the average number of initial cases in each group, with results summarized in Table S20. We use a slightly different methodology than that in the fall 2020 calibration because unlike fall 2020, arrival testing was carefully planned at the beginning of the spring 2021 semester as part of the arrival process. In addition, a significant fraction of the student population stayed in Ithaca during the winter break and took regular surveillance testing.

To model different behavior among students in taking their arrival tests, we partition students in each group into two categories: (1) those arriving from outside Ithaca and getting tested promptly upon arrival; (2) those arriving from outside Ithaca but not getting tested promptly, or those staying in the Ithaca over the winter break, taking regular surveillance testing but exempt from arrival tests. (See Table S20, col.a and col.d for the sizes of each category in each student group.)

We consider the initial free and infectious cases at the beginning of the simulation to be the union of

- those imported positive cases that received their first test promptly upon arrival but were missed by the arrival test (these cases belong to the first category);
- those cases imported to the Ithaca community but not tested promptly upon arrival, and those local infections during the arrival period of the simulation (these two kinds of cases belong to the second category).

First, we estimate the number of initial cases (Table S20, col.c) that belong to the first category. We infer the number of cases promptly tested upon arrival but missed by arrival tests from the observed arrival positive cases (Table S20, col.b). We classify a positive student case as an arrival positive case if it satisfies *all* of the following criteria:

- The student tested positive on their first test since 1/21/2021;
- The first (positive) test occurred between 1/21/2021 and 2/7/2021;
- The case was identified via contact tracing (including adaptive testing);
- The student was not in Ithaca during the winter;
- The student completed their first test within 3 days of their arrival date indicated in the spring 2021 semester checklist.

Then, based on the same methodology as in the fall 2020 calibration but assuming instead that the sensitivity of the arrival testing is 60% for AN sampling, we estimate that for every arrival positive case, there are 1.08 free and infected cases acting as the initial cases in the simulation. We assume that the arrival positive cases do not result in any secondary cases because they completed their first test upon arrival promptly.

Second, we estimate the number of initial cases that fall into the second category (Table S20, col.f). To do that, we calculate the prevalence level (including positive cases that were captured OR missed by arrival testing) among students that belong to the first category in each student group (Table S20, col.e). Then, assuming no heterogeneity in prevalence across the two categories of students, we estimate the number of positive cases among students in the second category by taking the product of the same prevalence estimate and the number of students in the second category. All of these positive cases in the second category are assumed to be part of the initial cases in the simulation.

The average number of initial cases in each student group (Table S20, col.g) is then the sum of estimated number of initial cases in the first and second categories, respectively.

Table S20. Average number of initial cases in each group for the spring 2021 semester.

Group	a: # students in the first category	b: # arrival positives	c: estimated # positive cases in the first category	d: # students in the second category	e: estimated prevalence	f: estimated # initial positives in the second category	g: average total number of initial positive cases
1	2707	10	10.8	622	0.342%	2.1	12.9
2	7355	43	46.4	1678	0.541%	9.1	55.5
3	231	1	1.1	303	0.401%	1.2	2.3
4	1236	5	5.4	3991	0.375%	14.9	20.3

Calibration Results We calibrate our model's projected infections to the actual trajectory within 4 subgroups from 1/21/2021 - 5/25/2021, as shown below. The total number of positive cases observed within the time period is described below and the trajectories are described in Figure S8.

- Group 1: 184, excluding 10 arrival positives;
- Group 2: 201, excluding 43 arrival positives;
- Group 3: 65, excluding 1 arrival positive;
- Group 4: 51, excluding 5 arrival positives.

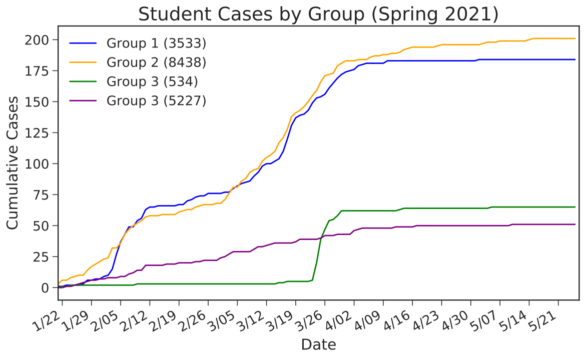

Fig. S8. Observed infections (excluding arrival positives) among students during the spring 2021 semester, shown for each of the four student groups.

As in the fall 2020 calibration, we tune the proportionality constant α so that it minimizes the log root mean-squared error of our model predictions.

Figure S9 shows the log root mean-squared error of our model predictions versus α. We see that when $\alpha = 0.8$, the lowest score is obtained.

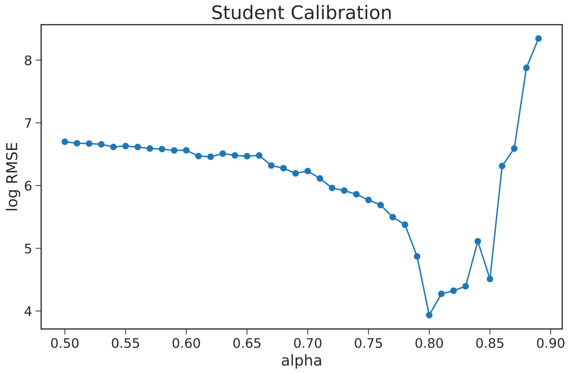

Fig. S9. For spring 2021, the log of the root mean-squared error (RMSE) of projected student infections versus α, the proportionality constant that multiplies the contact matrix to obtain the daily transmission rate.

Figure S10 shows the simulated trajectories (25 in each group) when $\alpha = 0.8$, in comparison to the actual trajectories for students cases in different subgroups. The actual trajectory appears quite high in these plots partly because we calibrate the actual trajectory to the *mean* simulated trajectory, so high simulated trajectories carry significant weight.

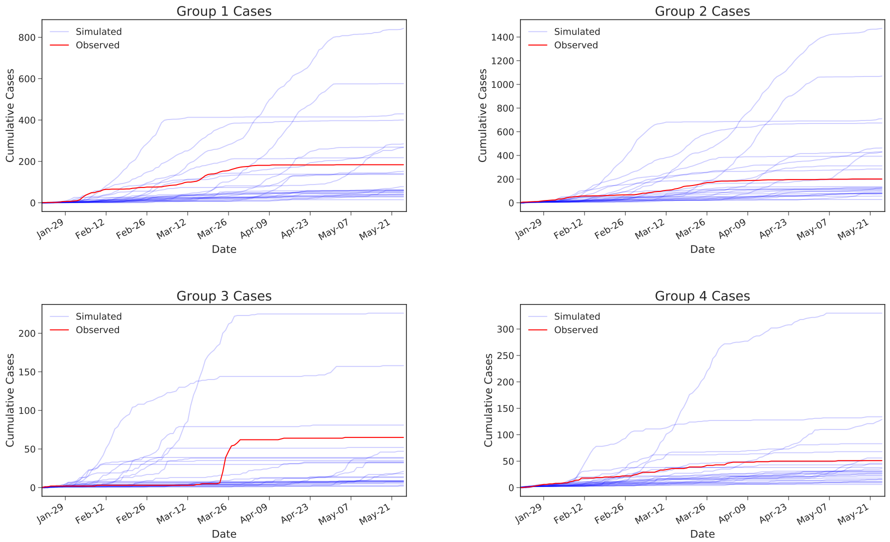

Fig. S10. Observed infection trajectories for each student group, over the course of the spring 2021 semester, plotted along with stochastic sample trajectories from the simulation under the estimated parameters.

D. Model Calibration for Employees in the Spring 2021 semester. The spring 2021 calibration for employees largely resembles the fall 2020 calibration. We use the same single-group simulation model consisting of all faculty and staff with population size 10283, and include all infections that occurred between January 21, 2021 and May 25, 2021.

Here we describe the parameters estimated directly from spring 2021 data in the model calibration for employee group.

Testing Frequency 0.146 per person per day. The same methodology as in the fall 2020 calibration is used.

Outside Infection Rate We use the same methodology as in the fall 2020 calibration to estimate outside infection rate. We continue to use time-varying outside infection rate (measured in infections per day) to address the heterogeneity in the number of outside infections across different weeks. The average daily infection rate over the entire simulation period is 9.9E-5, i.e., in a population of 10,000 people, we should expect to see 0.99 infections per day due to travel and interaction with the outside community.

Contact Tracing Effectiveness Parameters We use the same methodology as in the fall 2020 calibration and obtain the estimate of the contact tracing effectiveness parameter `cases_isolated_per_cluster` $= 0.035$.

Initial Prevalence We use an initial prevalence of zero among employees at the beginning of the spring 2021 semester. Although this likely underestimates the actual initial prevalence, we argue that the actual prevalence is low because employees took

regular surveillance tests (on average, 1x/week) even during the winter break. In addition, our outside infection rate estimates help to capture the cases imported to the Cornell community.

Calibration Results We calibrate our model's projected infections to the actual trajectory from 1/21/2021 - 5/25/2021, which is shown in Figure S11.

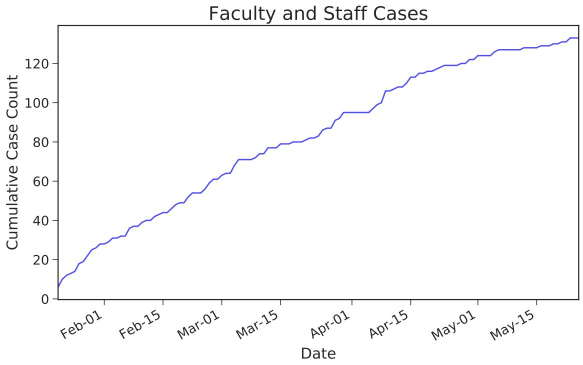

Fig. S11. Trajectory of employee infections from 1/21/2021 to 5/25/2021, the period used for spring 2021 calibration.

As in the fall 2020 calibration, we tune the daily transmission rate so that it minimizes the log root mean-squared error of our model predictions.

Figure S12 shows the log RMSE versus employee transmission rate. We see in this figure that when the daily transmission rate is zero, the lowest error is obtained. This is expected because most of the employee cases in the spring 2021 semester are considered outside infections, which are captured by the outside infection rate parameter. Hence, for the simulated trajectories to match the actual trajectory, we expect minimal transmission among employees on campus.

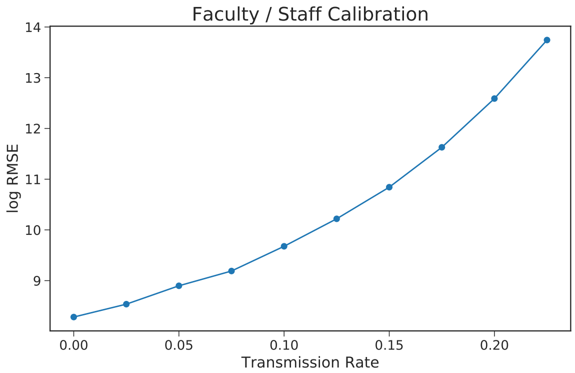

Fig. S12. For spring 2021, the log of the root mean-squared error (RMSE) of projected employee infections versus employee transmission rate.

Figure S13 shows 25 simulated trajectories when the daily transmission rate is zero, in comparison to the actual trajectory for faculty and staff cases. We observe that the observed case counts are reasonably well-represented by the simulation. The simulated trajectories fail to capture some of the cases occurring at the beginning of the spring 2021 semester but predict the cumulative case count well.

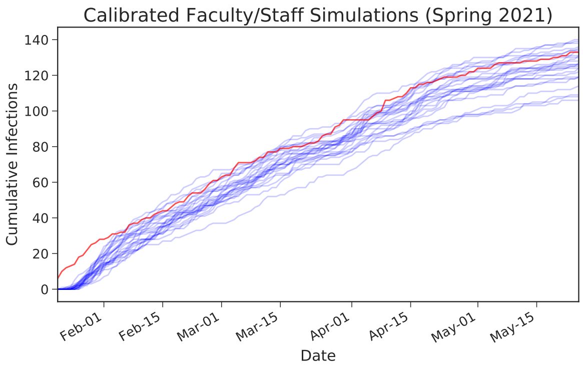

Fig. S13. Projections from our model (blue lines) using the calibrated daily transmission rate for the spring 2021 semester, compared with the observed infection trajectory (red line).

3. Parameter Uncertainty

This section presents our methodology for quantifying the effects of uncertainty in model parameters and additional results from applying this methodology not presented in the main paper.

We are specifically interested in the effect of parameter uncertainty on two outcomes: the safety of a residential semester as measured by the number of cases; and the relative safety of a residential semester compared to a virtual one, as measured by the difference in infections between these two instruction modes (residential infections - virtual infections). For both outcomes, a larger value is worse.

To quantify these effects, we perform the following steps:

1. Identify a set of key parameters and their associated uncertainty to define a (joint) prior distribution. There are 12 key parameters that govern the number of residential infections and an additional 4 parameters that govern the number of virtual infections. These 16 parameters and their corresponding 95% credible intervals are summarized in Table S21.

2. Construct linear approximations of functions relating the input parameters to 1) the median number of residential infections, and 2) the difference in the median number of infections between residential and virtual instruction.

3. Using the geometry of the prior distribution and the linear approximations constructed in Step 2, identify a family of 1-dimensional parameter configurations with varying levels of pessimism. For each level of pessimism q, and each of the two outcomes (residential infections, residential - virtual infections) identify a set of parameter configurations whose median outcome is equal to the q-quantile of this outcome under the prior, as predicted by the linear approximation. Then, for each q, select as representative the configuration in this set with the largest density under the prior.

A. Parameter Scenarios. We adapt ideas from *robust optimization* (34) to address parameter uncertainty, with the goal of identifying and understanding the worst possible outcome over the parameter configurations.

We begin by constructing an *uncertainty set* derived from reasonable *ranges* for each parameter (see the "lower bound" and "upper bound" columns in Table S21). These ranges induce a natural central point in the space of parameter configurations, where each parameter takes the value at the midpoint of its range. We place a joint multivariate normal prior with independent marginals on the parameters with mean at the central point. We assume the range for each parameter given in Table S21 is a symmetric 95% credible interval, i.e., the true parameter value lies in this interval with 95% probability.

The multivariate normal prior is used primarily for simplicity. We require a unimodal distribution with elliptical contours, the latter property of which permits straightforward calculation of pessimistic scenarios below. One could use other distributions with such contours, e.g., a multivariate t distribution. We chose the normal for simplicity and because the "core" of the prior distribution where most of the probability is concentrated, drives much of the analysis, which we believe is well captured through the normal prior. With a multivariate t with similar mean and spread we believe the outcomes would not have been substantially different. With regard to the issue about parameters potentially falling outside of meaningful ranges, such as the issue of non-negativity, we use rejection sampling to ensure that all sampled parameters fall within their meaningful range. Hence, the actual prior is a truncated multivariate normal distribution. Still, the exact form of the prior is arguable. To be more precise in what follows, we define the following notation:

- $x \in \mathbb{R}^n$: vector of parameters; $n = 12$ for the residential case and $n = 16$ for the residential-virtual case.

- LB_i, UB_i: lower and upper bound of parameter i, as specified in Table S21. By assumption, (LB_i, UB_i) is a symmetric 95% credible interval for parameter i and parameters are mutually independent under the prior.

Table S21. Parameter ranges. The first twelve are for the residential investigation; the last four are additional parameters for the virtual case. The last parameter, "virtual population size", is standardized to [0,1] which linearly interpolates between the lower and upper bounds.

Parameter	Meaning	Lower bound (LB)	Upper bound (UB)	Justification for choice of range
Asymptomatic probability multiplier	Multiplier applied to $P(\text{asymptomatic})$ for each group, other severity levels scaled accordingly	24/47	70/47	CDC planning scenarios range: (15%, 70%), we use upper bound from here and our estimate of 47% for US population to define range.
Initial prevalence multiplier	Multiplier applied to initial prevalence	0.5	1.5	Base estimate uses 10x under-reporting rate. Estimate at the time for most states was 6-10x, with a max of 23x. Our reasonable aggregate estimate is 5-20x under-reporting.
R0	The baseline transmission rate of the disease is calibrated to an estimate of R0	1	4	CDC planning scenarios indicated the best guess was 2.5 (center) and pessimistic estimate was 4 (upper bound).
Outside infection multiplier	Multiplier applied to outside infection rate	0.5	1.5	Reasonable range representing our uncertainty.
Daily self-report probability	Daily probability that symptomatic person will self-report	0.22	0.5	Lower bound estimate from CDC time for seeking care for flu (12). This is likely pessimistic due to public awareness. A reasonable upper bound is people reporting symptoms within 2 days on average.
Contact tracing multiplier	Multiplier on the effectiveness of contact tracing	1	2	Base estimate is from contact tracing effectiveness in Ithaca in the summer 2020. During the semester, we expect contact tracing to be slightly more effective (e.g. adaptive testing).
Contact tracing testing ratio	Number of individuals identified per contact trace from surveillance testing positive relative to a symptomatic self-report	0.5	1.5	Our baseline estimate is that contact tracing should be as effective in both scenarios. We construct a reasonable range with center 1.
Test sensitivity	Sensitivity of surveillance tests	0.4	0.8	Reasonable range based on our group testing protocol.
Test non-compliance	Probability a surveillance test will be skipped	0.05	0.15	All students signed a behavioral compact, giving the university the ability to enforce compliance.
Exposed time (days)	Expected time in Exposed state	1	3	Reasonable range based on disease progression.
Infectious time (days)	Expected time in Infectious state	2	4	Reasonable range based on disease progression.
Symptomatic time (days)	Expected time in Symptomatic / Asymptomatic state	11	13	Reasonable range based on disease progression.
Persistent non-compliance	Percent of students in a virtual scenario who would not enroll in the surveillance testing program	0.25	0.75	Since Cornell would have limited enforcement options, many students may not inform the university that they have returned to Ithaca.
Intermittent non-compliance	In a virtual scenario, percent of scheduled surveillance tests that will be skipped	0.25	0.75	The percent of surveillance tests being skipped will be higher than under the residential scenario since the university has limited enforcement.
Virtual transmissions per Day	Ratio of the transmission rate of students for the virtual scenario relative to the residential scenario	0.97	1.5	Due to the university's limited enforcement of masking, social distancing and testing, there will likely be an increase in transmission among students. The lower bound of 0.97 is explained in the virtual instruction section.
Virtual population size	The number of returning undergraduate and class based graduate students	0 (4500 UGs, 4770 GS other)	1 (7950 UGs, 5850 GS other)	Based on survey results of students. Lower bound is from proportions that replied "very or somewhat likely to return", upper bound students who answered "it depends / undecided".

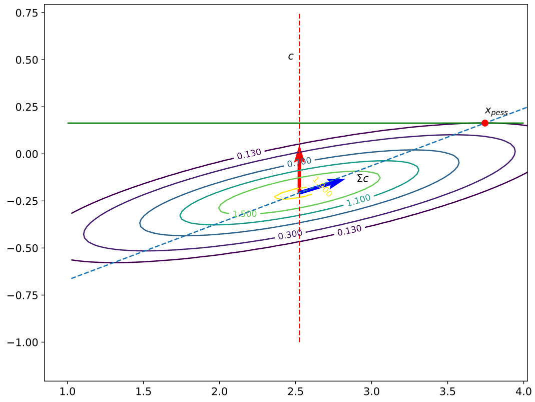

Fig. S14. Contours of the prior of the 12 parameters for the residential setting projected onto the space spanned by c (red arrow) and Σc (blue arrow). Without loss of generality, we align the vertical axis with the direction of c. The green line represents the hyperplane $A(y^*) = \{x : c_0 + c^T x = y^*\}$, which is perpendicular to c. The red dot represents $x(y^*)$, the unique point in $A(y^*)$ that lies on the line through μ in the direction Σc.

- $\Sigma = [\sigma_{ij}]$: the covariance matrix used in our multivariate normal prior. The components are specified by

$$\sigma_{ij} = \begin{cases} \sigma_i^2 & \text{if } i = j, \\ 0 & \text{otherwise.} \end{cases}$$

  Each standard deviation σ_i is derived from the range (LB_i, UB_i) that we assume for parameter i. By virtue of assuming this range defines a 95% credible interval and assuming a normal prior, the range is related to the standard deviation by the equality $\frac{1}{2}(UB_i - LB_i) = 1.96\sigma_i$.

- $\mu = [\mu_i]$: the mean of our multivariate normal prior, as well as the central point of our parameter ranges. $\mu_i = \frac{1}{2}(LB_i + UB_i)$ for $i = 1, \ldots, 12$.

Next we consider the development of the linear approximations. As described above, we are interested in two outcomes. The first outcome is the number of cases in a residential semester. The second outcome is the number of residential infections minus the number of virtual instructions. In both settings, the outcome is worse at larger values.

To estimate the outcome over the parameter space, we sample 2000 parameter vectors using Latin hypercube sampling over the hypercube defined by all 16 ranges. For each parameter vector, we run 50 residential and virtual semester simulations and calculate the median value of the outcome of interest. We then construct a linear approximation, $c_0 + c^T x$, of the median, using linear regression on the corresponding 12 or 16 parameters of interest (x). The coefficients and standard error for each parameter in the linear regressions are presented in Tables S22 and S23.

To summarize uncertainty, we develop a one-dimensional family of parameter configurations associated with increasingly pessimistic outcomes. For each $y \in \mathbb{R}$, we consider the set $A(y)$ of parameter configurations whose expected outcome under the fitted linear model is equal to y. By construction, such sets $\{A(y), y \in \mathbb{R}\}$ are hyperplanes normal to c and partition the parameter space into two half-spaces. We find y^* such that the associated half-space, over which the expected outcome under the linear model is less than or equal to y^*, contains a prior probability mass of 0.99. We then determine the pessimistic configuration by selecting the representative point in $A(y^*)$ with the highest prior density. Figure S14 provides a visualization of this setup that may prove helpful in interpreting the following more precise explanation of how we summarize uncertainty.

For any $y \in \mathbb{R}$, the set of parameter configurations with expected outcome equal to y under the linear model is the hyperplane

$$A(y) = \{x : c_0 + c^T x = y\}.$$

Consider the half-space defined by this hyperplane over which the expected outcome under the linear model is less than or equal to y, $\{x : c_0 + c^T x \leq y\}$. Let $q(y) = P(c_0 + c^T X \leq y)$ be the prior probability mass in this half space, where $X \sim \mathcal{N}(\mu, \Sigma)$. Let y^* be such that $q(y^*) = 0.99$, so that y^* is such that the median outcome is less than y^* with prior probability 0.99. To find y^*, recall that $X \sim \mathcal{N}(\mu, \Sigma)$, so $c_0 + c^T X \sim \mathcal{N}(c_0 + c^T \mu, c^T \Sigma c)$, and then

$$\begin{aligned} & P(c_0 + c^T X \leq y^*) = 0.99 \\ \iff & P\left(\frac{c^T X - c^T \mu - c_0}{\sqrt{c^T \Sigma c}} \leq \frac{y^* - c^T \mu - c_0}{\sqrt{c^T \Sigma c}}\right) = 0.99 \\ \iff & \frac{y^* - c^T \mu - c_0}{\sqrt{c^T \Sigma c}} = \Phi^{-1}(0.99) \\ \iff & y^* = \Phi^{-1}(0.99)\sqrt{c^T \Sigma c} + c^T \mu + c_0. \end{aligned}$$

Let $x(y^*)$ be the point with the largest prior density in the hyperplane $A(y^*)$. We claim that $x(y^*)$ is the unique point in $A(y^*)$ lying on the line through μ in the direction Σc, that is $x(y^*) \in \{\mu + \lambda\Sigma c : \lambda \in \mathbb{R}\}$. Why? Maximizing the density over $A(y^*)$ is equivalent to minimizing the quantity $(x-\mu)^T\Sigma^{-1}(x-\mu)$ over all $x \in A(y^*)$, i.e., over all points x satisfying $c_0 + c^Tx = y^*$. To find the optimum, define the Lagrangian $L(x;\eta) = (x-\mu)^T\Sigma^{-1}(x-\mu) - \eta(c_0 + c^Tx - y^*)$; the optimum is characterized by the equation $\nabla_x L(x;\eta) = 0$, for some Lagrange multiplier $\eta \in \mathbb{R}$. The gradient of the Lagrangian is $\nabla_x L(x;\eta) = 2\Sigma^{-1}(x-\mu) - \eta c$, so the optimal point is given by $x(y^*) = \mu + \frac{\eta}{2}\Sigma c$, which is on the line through μ in the direction Σc as originally claimed.

We can thus find $x(y^*)$ as the unique point in the intersection of the hyperplane $A(y^*)$ and the ray $\{\mu + \lambda\Sigma c : \lambda \in \mathbb{R}\}$. We find that $\lambda = \Phi^{-1}(0.99)/\sqrt{c^T\Sigma c}$, and so the pessimistic point is given by

$$x(y^*) = \mu + \frac{\Phi^{-1}(0.99)}{\sqrt{c^T\Sigma c}}\Sigma c. \tag{2}$$

We follow this same approach to create a range of parameter scenarios with varying levels of pessimism $q \in (0,1)$, by substituting q for 0.99 in the derivation above. We refer to the resulting parameter scenario as the *q-quantile pessimistic point*, and (in an abuse of notation) denote it as $x(q)$. The expression for this parameter scenario is obtained by substituting q for 0.99 in Equation 2:

$$x(q) = \mu + \frac{\Phi^{-1}(q)}{\sqrt{c^T\Sigma c}}\Sigma c.$$

This parameter scenario is such that the prior probability is q of seeing a parameter configuration with fewer infections than $x(q)$, assuming that the simulator's response is given by the fitted linear model.

Figure S15 shows the level-0.99 pessimistic scenarios where the outcome is the number of infections in a residential semester (12 parameters) and the difference in the number of infections between a residential and virtual semester (16 parameters), respectively.

B. Assessing Pessimism Level of Parameter Scenarios. We use the term "true pessimism level" of a scenario to refer to the probability under the prior of drawing a parameter configuration whose infections are worse than this scenario. The q-pessimistic scenarios described above were obtained assuming that the simulator response follows the fitted linear model, while in reality the simulator's response may be non-linear in the model parameters and parameters may interact in ways not captured by the linear model. Thus, the true pessimism level of a q-pessimistic scenario might not be q.

In this section, we support the claim that the true pessimism level of a q-pessimistic scenario actually is typically close to q, justifying their use.

We focus on the residential outcome, where there are 12 parameters. For each parameter configuration $x(q)$ with q ranging over $q \in \{0.01, 0.05, 0.1, \cdots, 0.9, 0.95, 0.99\}$, we run 20 simulation replications and record the median number of infections in a residential semester, which we denote as $\hat{y}^*(q)$.

Then, to estimate true pessimism levels, we sample $N = 1000$ parameter configurations from the 12-dimensional multivariate normal prior. For each sampled parameter configuration x_i, we run 20 simulation replications and record the median number of infections y_i in a residential semester. We let $Y = \{y_i\}_{i=1}^{1000}$ denote the set of median simulation outcomes. Note that Y does not depend on any modeling assumptions of the way simulation outcomes depend on parameter configurations such as linearity.)

Next, for each q, we use Y to estimate the true pessimism level of $x(q)$, denoted by $r(q)$. This is the fraction of outcomes (median infections) in Y that are smaller than the outcome at $x(q)$. Mathematically, $r(q) = |\{y_i \in Y : y_i \leq \hat{y}^*(q)\}|/N$, where $|\cdot|$ denotes the cardinality of a set. If $r(q)$ is close to q, then the pessimism level q claimed relying on the linear model is close to the actual pessimism level $r(q)$ of the resulting scenario.

Figure S16 shows the estimated values of $r(q)$ vs. q. For all values of q evaluated, the deviation of $r(q)$ from q is within a small range. In particular, $r(q)$ and q match each other well for q close to 1. This demonstrates that the true pessimism level associated with the 99% pessimistic point $x(y^*)$ is close to 99%.

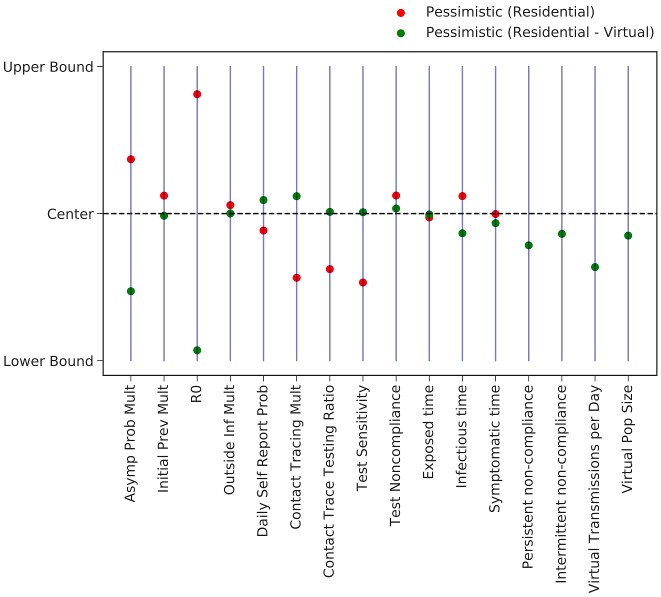

Fig. S15. Plot depicts the relative parameter values of both pessimistic scenarios.

C. Scenarios from June 2020 report. As noted in Sections 1B and 1E of the paper, the nominal scenario reported here differs from the one reported in our June 2020 report (1). The 2020 nominal scenario was developed under time pressure and was intended to play a central role in the thinking of decision makers. It was therefore chosen to be somewhat conservative (meaning erring on the side of increased infections) with regard to a number of key parameters, especially contact-tracing parameters, as opposed to the nominal scenario presented here that is instead meant to represent our best estimate of the parameter values. Except for those key parameters, the 2020 nominal scenario resembles the nominal scenario reported here. The 2020 report also defined optimistic and pessimistic scenarios that, likewise, do not coincide with scenarios presented here. Table S24 lists the parameters for the scenarios explored in the 2020 report. See, also, Table S25.

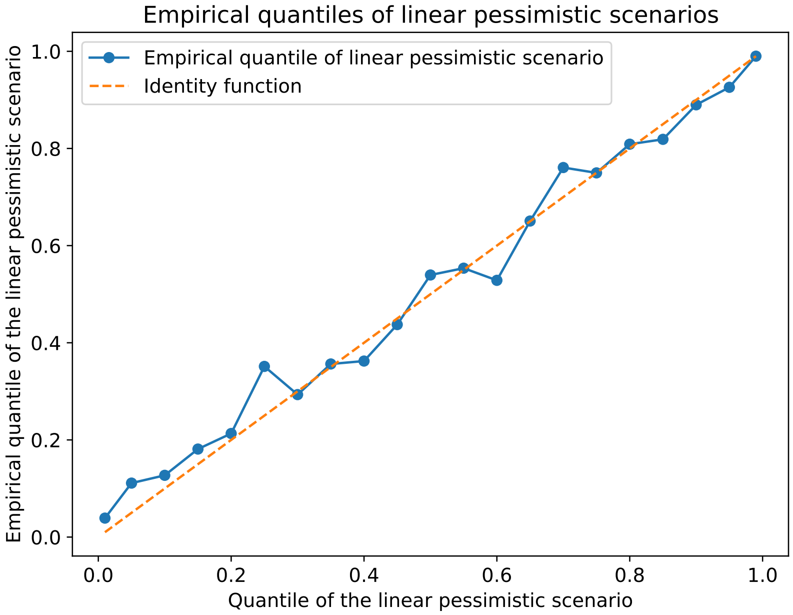

Fig. S16. For each pessimism level q, a model-free simulation-based estimate of the probability $r(q)$ under the prior of having a parameter configuration whose median number of infections is worse than the median number of infections under the pessimistic scenario $x(q)$ with pessimism level q. The scenario $x(q)$ assumes that the simulator's response is linear in the parameters and so $r(q)$ may differ from q. The data pictured here suggests that $r(q)$ is close to q despite non-linearities in the simulator's response to parameters. Estimates of $r(q)$ were calculated at $q = 0.01, 0.05, 0.1, \cdots, 0.9, 0.95, 0.99$.

Table S22. Fitted linear coefficient and computed pessimistic value for the residential instruction scenario.

Parameter	Linear coefficient	Std. Err	$P > \|t\|$	Coef $\times$ range	Pessimistic value
Regression const	1014.7	429.2	0.018		
Asymptomatic prob multiplier	570.5	61.0	0.000	558.3	1.18
Initial prevalence multiplier	184.4	59.8	0.002	184.4	1.06
R0	409.1	19.9	0.000	1227.3	3.72
Outside infection multiplier	86.7	59.5	0.146	86.6	1.03
Daily self-report probability	-623.0	213.0	0.003	-174.4	0.34
Contact tracing multiplier	-659.7	59.6	0.000	-659.7	1.28
Contact tracing testing ratio	-571.2	59.6	0.000	-571.2	0.81
Test sensitivity	-1771.7	149.2	0.000	-708.7	0.51
Test non-compliance	1855.8	596.4	0.002	185.6	0.11
Exposed time (days)	-19.7	29.8	0.510	-39.3	1.97
Infectious time (days)	89.9	29.8	0.003	179.8	3.12
Symptomatic time (days)	-2.3	29.8	0.939	-4.6	12.0

Table S23. Fitted linear coefficient and computed pessimistic value for residential - virtual infections.

Parameter	Linear coefficient	Std. Err	$P > \|t\|$	Coef $\times$ range	Pessimistic value
Regression const	19870	827.5	0.000		
Asymptomatic prob multiplier	-4341.5	109.3	0.000	-4249.2	0.74
Initial prevalence multiplier	-119.8	106.9	0.263	-119.8	0.99
R0	-2493.7	35.6	0.000	-7481.1	1.11
Outside infection multiplier	6.1	106.7	0.954	6.1	1.00
Daily self-report probability	2645.8	380.6	0.000	740.8	0.37
Contact Tracing multiplier	951.1	106.6	0.000	951.1	1.56
Contact Tracing testing ratio	92.4	106.8	0.387	92.4	1.00
Test sensitivity	184.9	267.0	0.489	74.0	0.60
Test non-compliance	2769.7	1066.2	0.009	277.0	0.10
Exposed time (days)	-24.5	53.3	0.645	-49.1	1.99
Infectious time (days)	-538.2	53.2	0.000	-1076.4	2.87
Symptomatic time (days)	-261.6	53.3	0.000	-523.2	11.94
Persistent non-compliance	-3474.3	213.4	0.000	-1737.1	0.45
Intermittent non-compliance	-2218.5	213.0	0.000	-1109.2	0.47
Virtual transmissions per Day	-5522.9	201.0	0.000	-2927.1	1.14
Virtual population size	-1207.2	106.9	0.000	-1207.2	0.43

Table S24. Parameter values for scenarios used in summer 2020 analysis. In this analysis the daily self-report probability should have been 0.22, but we used 0.18 due to a calculation error.

**Parameter**	**2020 Optimistic**	**2020 Nominal**	**2020 Pessimistic**
Asymptomatic prob multiplier	1	1	1
Initial prevalence multiplier	1	1	1
R0	1.75	2.5	3.25
Outside infection multiplier	1	1	1
Daily self-report probability	0.18	0.18	0.18
Contact tracing multiplier	1	1	1
Contact tracing testing ratio	0.5	0.5	0.5
Test sensitivity	0.7	0.6	0.5
Test non-compliance	0.1	0.1	0.1
Exposed time (days)	2	2	2
Infectious time (days)	3	3	3
Symptomatic time (days)	12	12	12

D. Comparison of Prior to Calibrated Outcomes. Table S25 summarizes key parameter differences between fall 2020 nominal, fall 2020 pessimistic, summer 2020 nominal and calibrated fall 2020 scenarios. The calibrated fall 2020 scenario includes parameter values that were directly estimated according to data from Fall 2020 or calibrated based on both our simulation model and data. Below we summarize how the 5 calibrated values compared to our prior range.

- Transmissions per day: The students with the highest transmission rate (those with Greek-life or varsity athletics affiliation) were within our prior range for transmissions. However, we overestimated the transmission rate for the remaining students.
- Cases found per contact trace: the effectiveness of contact tracing was very close to our nominal estimate.
- Initial prevalence: The students with the highest initial prevalence (those with Greek-life or varsity athletics affiliation) were within our prior range for initial prevalence. However, we overestimated the initial prevalence for the remaining groups.
- Outside infection rate: In the calibrated model, our definition for outside infection rate changed since we no longer explicitly modeled an Ithaca sub-population. Therefore, in the calibrated model an outside infection corresponds to any infection that originates outside the Cornell community. In all other scenarios, an outside infection refers to an infection from outside the Cornell or Ithaca community. Therefore, our prior range does not map conveniently to the calibrated definition.
- Test compliance for students: We underestimated the test compliance among students.

Since the groups changed between the uncertainty analysis and calibrated scenarios, some of the original 12 parameters in the uncertainty analysis are not appropriate for describing the calibrated scenario. For example, we used an outside infection multiplier to adjust all outside infection rates together in our uncertainty analysis. However, during our calibration, we arrived at group-specific rates which could not be mapped back to a single multiplier value. Therefore, we have replaced some of the 12 uncertainty parameters with new parameters that describe the same quantity (typically in different units).

As articulated in the faculty and staff calibration section, we assume that test compliance among this group is 1. This is because in the calibration for this group the testing frequency was directly estimated from data, which implies perfect compliance in the calibration simulations. Lastly, we used 0.18 as the daily self-report probability in summer 2020 scenarios because of a calibration error.

Table S26 summarizes the key calibrated parameters from the fall 2020 and spring 2021 semesters. The transmission rate and initial prevalence are higher in the spring 2021 semester than in the fall 2020 semester due to the new virus variants, COVID fatigue, increased social gatherings, etc.

Table S25. Summary of key parameter differences between fall 2020 calibrated, fall 2020 nominal, fall 2020 pessimistic (residential), and summer 2020 nominal scenarios. Blue indicates values calibrated directly to data and purple shows values calibrated via simulation. All remaining values are determined by assumption.

Parameter	Fall 2020 calibrated	Prior range	Fall 2020 nominal	Fall 2020 pessimistic (residential)	Summer 2020 nominal
Transmissions per day	0.3742 (Greek + Athlete)<br>0.0867 (UG other)<br>0.0441 (GS)<br>0.11 (Faculty / Staff)	0.1217-0.4869 (UG Dorm)<br>0.0878-0.3512 (UG Off)<br>0.0528-0.2110 (GS research)<br>0.0726-0.2906 (GS class)<br>0.0705-0.2819 (FS student)<br>0.0328-0.1310 (FS not student)<br>0.0297-0.1187 (FS off)	0.3043 (UG Dorm)<br>0.2195 (UG Off)<br>0.1319 (GS research)<br>0.1816 (GS class)<br>0.1762 (FS student)<br>0.0819 (FS not student)<br>0.0742 (FS off)	0.4291 (UG Dorm)<br>0.3095 (UG Off)<br>0.1860 (GS research)<br>0.2561 (GS class)<br>0.2622 (FS student)<br>0.1219 (FS not student)<br>0.1104 (FS off)	0.3043 (UG Dorm)<br>0.2195 (UG Off)<br>0.1319 (GS research)<br>0.1816 (GS class)<br>0.1762 (FS student)<br>0.0819 (FS not student)<br>0.0742 (FS off)
Cases found per contact trace	1.329	0.92 - 1.84	1.38	1.214	0.92
Contact tracing testing ratio	1	0.5-1.5	1	0.84	0.5
Initial prevalence	0.163% (Greek + Athlete)<br>0.040% (UG other)<br>0 (GS + Faculty / Staff)	0.095% - 0.285% (UG + GS class)<br>0.0575% - 0.1725% (GS research)<br>0.04% - 0.12% (Faculty / Staff)	0.19% (UG + GS class)<br>0.115% (GS research)<br>0.08% (Faculty / Staff)	0.20% (UG + GS class)<br>0.121% (GS research)<br>0.084% (Faculty / Staff)	0.19% (UG + GS class)<br>0.115% (GS research)<br>0.08% (Faculty / Staff)
Asymptomatic probability multiplier	1	24/47-70/47	1	1.18	1
Outside infection rate	1.42×10^{-5} (Greek + Athlete)<br>7.11×10^{-6} (UG other)<br>6.45×10^{-6} (GS)<br>Weekly rate calibrated to data (Faculty / Staff)	$0.6 \times 10^{-5} - 1.8 \times 10^{-5}$	1.2×10^{-5}	1.27×10^{-5}	1.2×10^{-5}
Daily self-report probability	0.36	0.22-0.5	0.36	0.34	0.18
Test sensitivity	0.6	0.4-0.8	0.6	0.51	0.6
Test compliance	0.974 (Students)<br>1 (Faculty / Staff)	0.85-0.95	0.9	0.89	0.9
Exposed time	2	1-3	2	1.97	2
Infectious time	3	2-4	3	3.12	3
Symptomatic time	12	11-13	12	12.0	12

Table S26. Summary of key calibrated parameters from the fall 2020 and spring 2021 semesters. Transmission rate was calibrated via simulation and all other parameters were calibrated directly from data.

Parameter	Fall 2020	Spring 2021
Transmissions per day	0.37 (Greek + Athlete)<br>0.09 (UG other)<br>0.04 (GS)<br>0.11 (Faculty / Staff)	0.62 (Greek + Athlete)<br>0.40 (UG other)<br>0.32 (MBA)<br>0.10 (GS other)<br>0 (Faculty / Staff)
Cases found per contact trace	1.329	0.854
Initial prevalence	0.163% (Greek + Athlete)<br>0.040% (UG other)<br>0 (GS + Faculty / Staff)	0.388% (Greek + Athlete)<br>0.614% (UG other)<br>0.431% (MBA)<br>0.388% (GS other)<br>0 (Faculty / Staff)
Outside infection rate	1.42×10^{-5} (Greek + Athlete)<br>7.11×10^{-6} (UG other)<br>6.45×10^{-6} (GS)<br>Weekly rate calibrated to data (Faculty / Staff)	1.68×10^{-5} (Greek + Athlete)<br>2.66×10^{-6} (UG other)<br>4.49×10^{-5} (MBA)<br>1.53×10^{-6} (GS other)<br>Weekly rate calibrated to data (Faculty / Staff)

E. Sensitivity Analysis for Individual Parameters. This section includes sensitivity analysis for model inputs. For the first 12 parameters, we show the sensitivity of residential infections and for the final 4 we show the sensitivity for virtual infections.

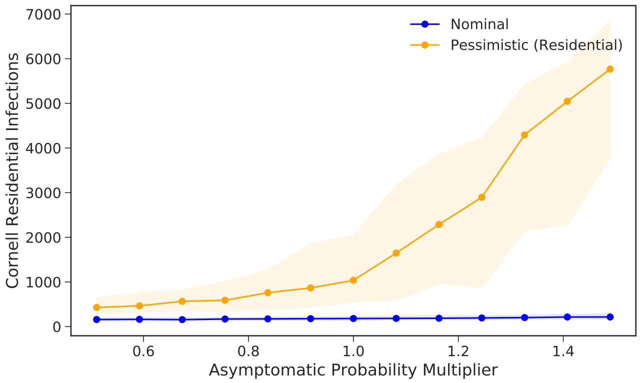

(a) Asymptomatic probability

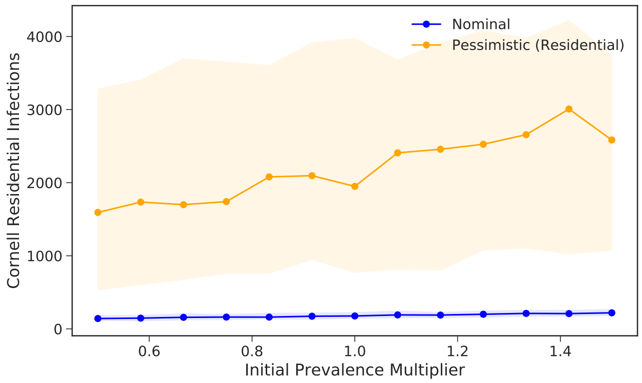

(b) Initial prevalence

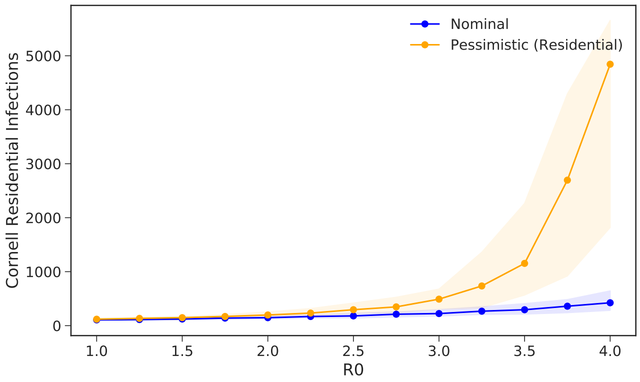

(c) R0

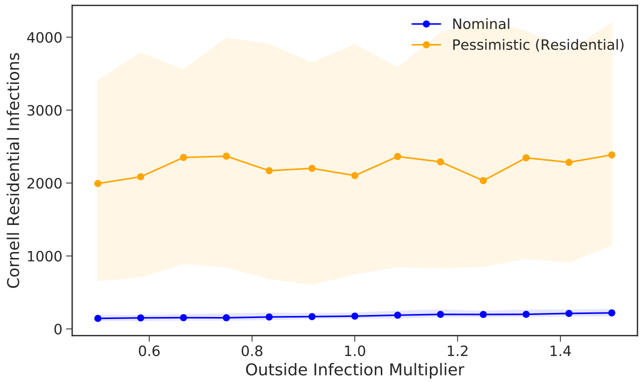

(d) Outside infection rate

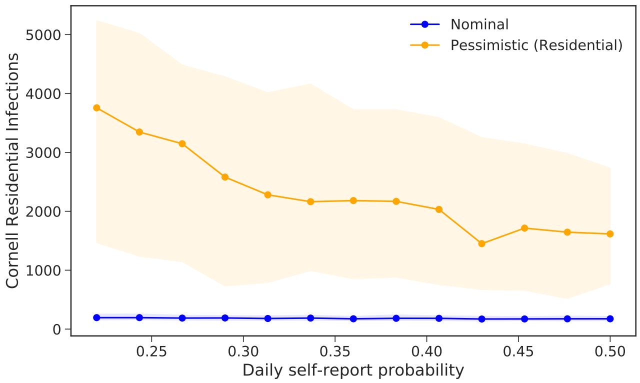

(e) Daily self-report probability

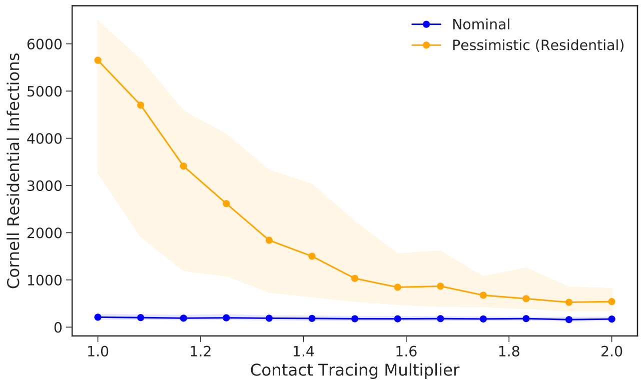

(f) Contact tracing effectiveness

Fig. S17. Each plot depicts the 50th percentile of infections, with a wider range corresponding to the 10-90th percentile range, as the stated parameter varies, for both the nominal and pessimistic (residential) scenarios.

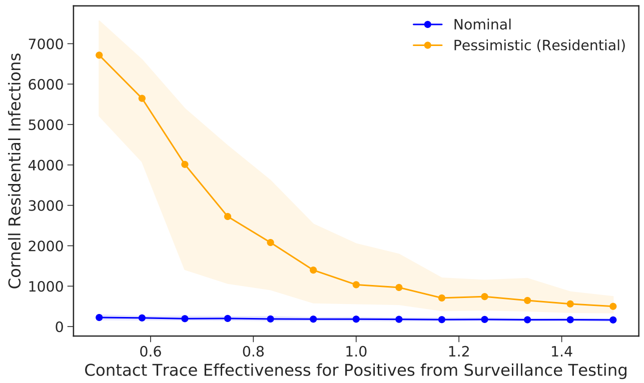

(a) Contact tracing effectiveness for surveillance testing positives

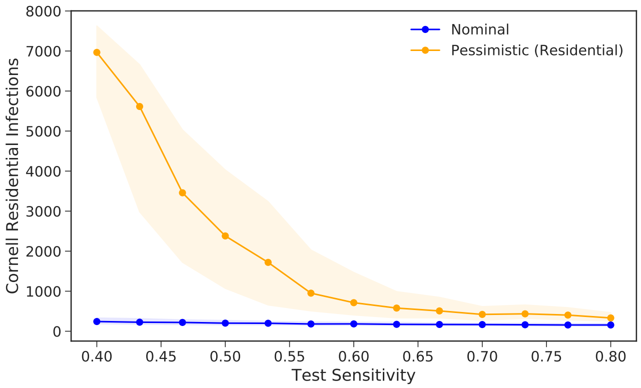

(b) Test sensitivity

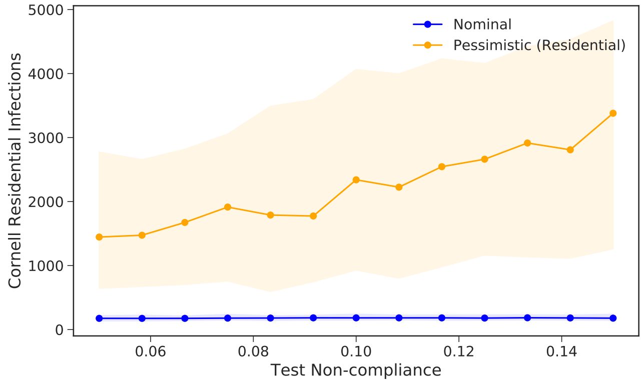

(c) Test non-compliance rate

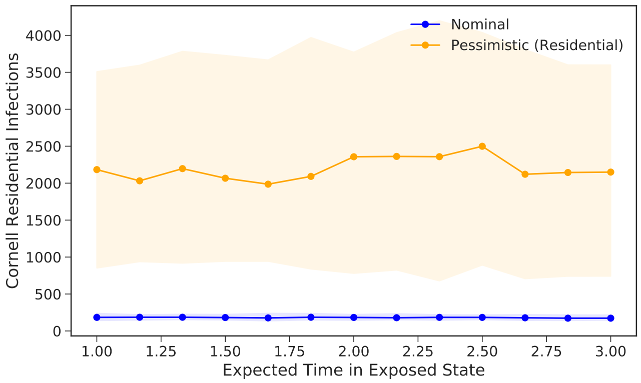

(d) Expected time in Exposed state

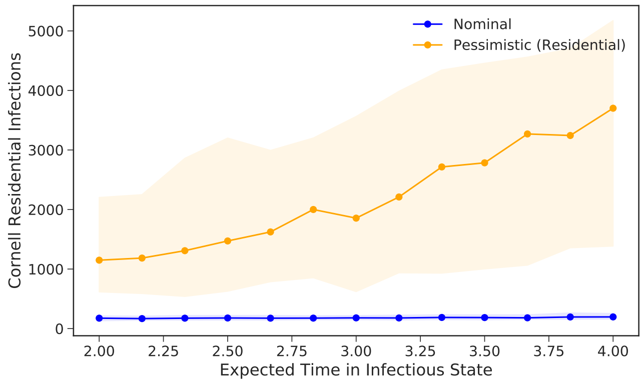

(e) Expected time in Infectious state

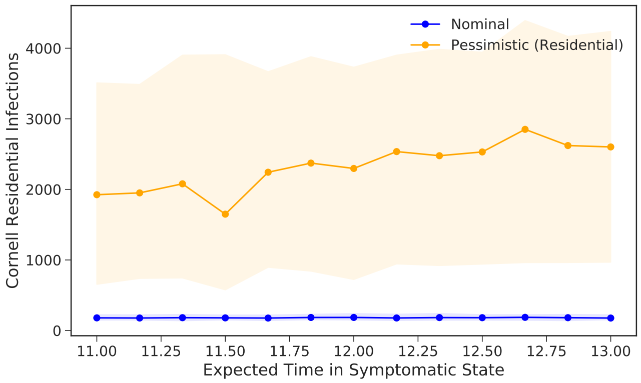

(f) Expected time in Symptomatic and Asymptomatic states

Fig. S18. Each plot depicts the 50th percentile of infections, with a wider range corresponding to the 10-90th percentile range, as the stated parameter varies, for both the nominal and pessimistic (residential) scenarios.

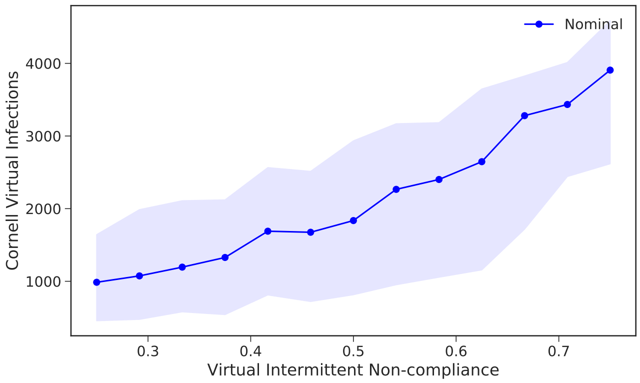

(a) Intermittent non-compliance in virtual scenario

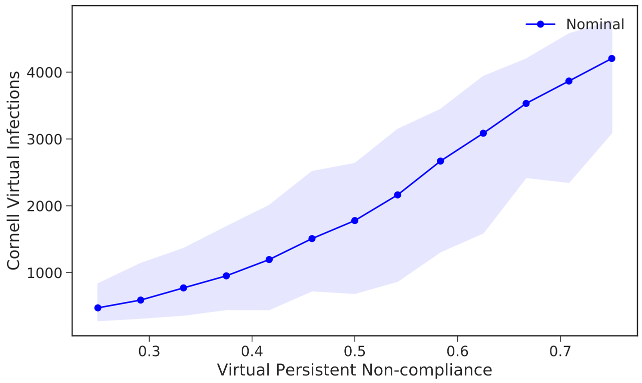

(b) Persistent non-compliance in virtual scenario

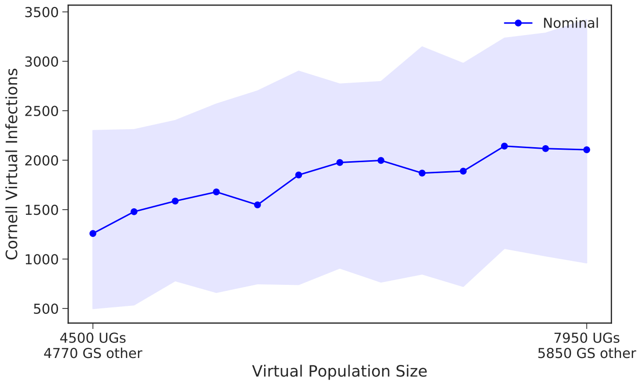

(c) Population size in virtual scenario

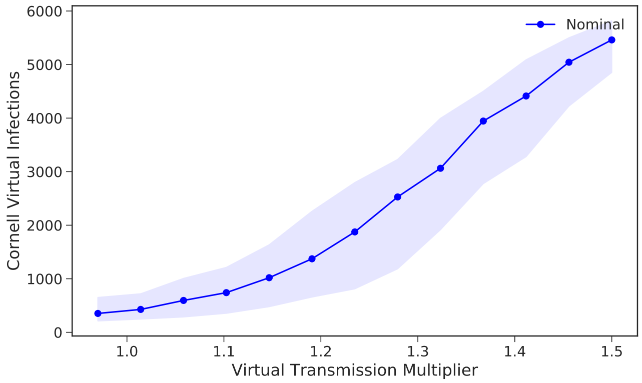

(d) Virtual scenario transmission multiplier

Fig. S19. Each plot depicts the 50th percentile of virtual instruction infections, with a wider range corresponding to the 10-90th percentile range, as the stated parameter varies for the nominal scenario. Non-monotonicity is due to simulation error.

F. Correlation of Infection and Hospitalization metrics. In this section, we present graphs that demonstrate that the simulated number of Cornell infections is positively correlated with the number of Ithaca infections and Cornell and Ithaca hospitalizations. Due to this correlation, we use the number of Cornell infections as our primary metric.

In Figure S20, each point corresponds to a parameter vector sampled from the prior described earlier in this section.

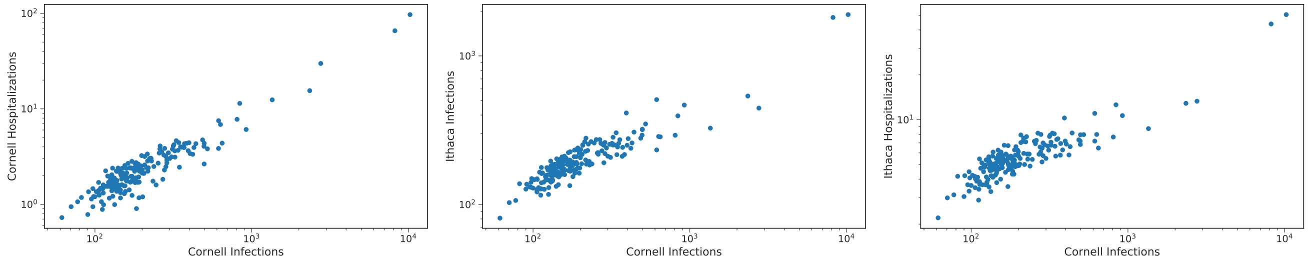

Fig. S20. Plot depicts the correlation of alternative metrics (Cornell hospitalizations, Ithaca hospitalizations, Ithaca infections) with the number of Cornell infections for parameter vectors sampled from the prior distribution.

4. Bayesian Analysis for Fall 2021 Projections

This section describes how we use our model to explore the interventions needed in the fall 2021 semester. We leverage information gathered from fall 2020 to the present and adjust for changes such as the Delta variant and vaccination level. Then, we perform a Bayesian analysis on the key uncertain parameters. To obtain the prior, we place ranges on each of these parameters, which then induce a prior using the same methodology for modeling the fall 2020 semester. We then sample parameters from the prior, run simulations at each parameter configuration, and approximate the posterior distribution using a heuristic choice of likelihood function. Sampling parameters from the approximated posterior distribution and simulating trajectories based on these sampled parameter configurations provides potential epidemic outcomes for the fall 2021 semester.

A. Parameter Adjustments. To model the spread of COVID-19 at Cornell in Fall 2021, we use the calibrated parameters from Fall 2020 and make the following adjustments:

1. Delta adjustment: We increase the transmissibility of the virus by a factor of 2.5 because of the delta variant. This factor is estimated based on the estimated R0 of 5-7 for the delta variant (35) and the estimated R0 of 2.5 for the original strain (11). Taking the middle value of the R0 range for the delta variant and dividing it by the R0 for the original strain gives 2.4. We use a slightly more pessimistic value of 2.5 as our estimate.

2. Initial Prevalence: we estimate a range for initial prevalence in each student group as described below.

3. We assume that 95% of students are vaccinated. This is lower than the student vaccination rate in steady state during the bulk of the semester, once student vaccinations upon arrival are complete. Vaccination decreases the probability that a person becomes infected when exposed and also decreases the rate at which they infect others when infected. The precise effect of the vaccine is uncertain and is controlled through two parameters described below.

4. The outside infection rate and number of contacts per day are increased relative to fall 2020 because of a relaxation in pandemic restrictions and changing attitudes to risk.

5. Contact tracing effectiveness (the number of positives isolated per contact trace) is altered because of a change to quarantine and isolation protocols (asymptomatic vaccinated close contacts are not quarantined) and because of the challenges presented to contact tracers by relaxation of social distancing and the increase in contacts it creates.

6. On August 29, 2021 the university changed policies and the simulation reflects these changes.

    - Delay in processing tests was reduced from 2 days to 1 day.
    - At the beginning of the semester, all vaccinated students were tested once a week and unvaccinated students were tested twice a week. The testing policy was updated so that all greek-affiliated students and varsity athletes were tested twice a week.

7. Add new parameters to reflect vaccination and the impact of relaxing social distancing. The parameters are detailed in Table S27.

B. Parameter Range Justification. We identify the key parameters with uncertainty and place ranges on each of these parameters, summarized in Table S27.

Vaccine Susceptibility Multiplier We collect estimates from the literature, including 42% (36), 79% (37), 88% (38), 40% (39) for Pfizer and 76% (36), 66% (40) for Moderna. We aggregate these estimates using a mixture model, accounting for the number of observations and uncertainty reported for each of them.

This estimate is optimistic in the sense that some of these results are measured shortly after vaccination, while (39) observed that the protection provided by vaccination decays over time.

This estimate is conservative in that the studies above were performed on general populations. Cornell has a larger fraction of young people, and vaccine efficacy was observed to be higher for younger people and lower for older (39). However, there is not sufficient evidence in the literature to support further investigation of age-stratified vaccine efficacy.

Vaccine Transmission Multipliers The literature reports varying results, ranging from a 2.8-4.5 fold reduction in viral load (41), 40%-50% reduction of transmission risk (42) to no reduction in viral load (43) or peak viral load (39). Given the significant uncertainty around this parameter, we use 0% reduction as a pessimistic estimate and 75% reduction as an optimistic estimate (consistent with a 4 fold reduction in viral load).

Contact Multiplier We use the SafeGraph foot traffic data to estimate the mean close contact multiplier modeling the elevation in contacts due to loosening social distancing interventions. We find that the foot traffic in Ithaca Collegetown in Fall 2019 is 80% higher than that in Fall 2020. Assuming that people's physical contact in Fall 2021 returns to the same level seen in Fall 2019 and foot traffic is a reasonable proxy for physical contact, we estimate that the mean close contact multiplier is 1.8.

We estimate an upper bound for the close contact multiplier by comparing the transmission of COVID-19 in the US in fall 2020 to its basic reproduction number R0.

Table S27. Parameter ranges for fall 2021 simulations.

Parameter	Meaning	Lower bound (LB)	Upper bound (UB)
Vaccine Susceptibility Multiplier	In an interaction with an infectious person, if the exposed person is vaccinated, what is the reduction in risk of becoming infected?	9.8%	94%
Vaccine Transmission Multiplier	In an interaction with a susceptible person, if the source is vaccinated and infectious, what is the reduction in risk of their becoming infected?	25%	100%
Contact Multiplier	Relative to Fall 2020, how much more physical contact are students having with others? This is realized both through interacting with more people and through reduced masking (which exposes others to more respiratory particles). 1x corresponds to the same amount of transmission per day as Fall 2020. The effect of the delta variant is modeled separately.	0.9×	2.7×
Outside Infection Rate Multiplier	Relative to Fall 2020, how often do we expect cases to be imported from the outside community (Ithaca and beyond)?	1×	5×
Contact Tracing Effectiveness Multiplier	Relative to Fall 2020, how many positive individuals are we finding and preventing from infecting others per student found via surveillance or symptomatic self-reporting? This implicitly includes the effect of both contact tracing and adaptive testing.	0.5×	1.5×
Initial Prevalence	What percentage of students are infected upon arrival?	0.3%	0.54%

The R0 for the original strain of COVID-19 is best estimated to be 2.5 (11). The effective reproduction number (Rt) in fall 2020 is lower bounded by 0.9 (44).

Assuming that reduction in transmission above results from social distancing interventions, we can estimate that loosening social distancing interventions leads to an increase in contacts by a factor of 2.5 / 0.9 = 2.7. This estimate is considered to be the upper bound for the close contact multiplier because it ignores the effects of other interventions such as contact tracing in reducing transmission and therefore overestimates the elevation in contacts due to loosening social distancing interventions.

We then set the lower bound by assuming a symmetric credible interval centered at 1.8. This provides a lower bound of 1.8 - (2.7-1.8) = 0.9. Thus we use (0.9, 2.7) as the (lower bound, upper bound) range for the close contact multiplier.

Outside Infection Rate Multiplier With the relaxation of social distancing guidelines and travel restrictions in Fall 2021, it was possible there would be an increase in the rate of cases imported into the Cornell community. Optimistically, this rate would be the same as a year prior, and pessimistically this rate would be 5 times higher. The value 5 is chosen somewhat arbitrarily and as our posterior analysis shows, is a reasonable upper bound.

Contact Tracing Effectiveness Multiplier The “Contact Tracing Effectiveness Multiplier” determines the number of additional positive individuals identified per contact trace relative to Fall of 2020. In constructing a prior on this parameter, there are two countervailing effects:

- The amount of contact is larger, which leads to more close contacts.
- Health department policies on quarantine have changed and close contacts are not quarantined if they are vaccinated and asymptomatic.

We set the range to (0.5 to 1.5), reflecting a prior belief that the number of positives contained per contact trace is within the range of 50% to 150% of the fall 2020 number with 95% probability.

Initial Prevalence We calibrate the initial prevalence of our model to the number of observed positives detected by a student’s first test of the semester. The upper bound counts all cases which tested positive on their first test. However, the data illustrated that there were likely some clusters related to Greek letter organizations. The lower bound is derived by counting only non-Greek letter organization positives.

C. Posterior Approximation and Projections. Here we describe a Bayesian analysis that leverages recently observed student case counts in fall 2021 at Cornell to approximate the posterior distribution for the parameters. We first specify a prior distribution based on the ranges described above. Then, sampling parameters from this prior and running simulations at each parameter configuration provides, via a heuristic choice of likelihood function, the means to update the prior to a posterior distribution. Equipped with an approximation of the posterior distribution, we use selected sets of parameters from the posterior approximation and simulate potential trajectories based on those sets of parameters. These trajectories represent possible infection trajectories over the fall 2021 semester.

Sampling from the Prior Let $\boldsymbol{\theta}$ be a vector denoting a parameter configuration. We think of each uncertainty range in Section B above as the 95% credible interval for a normal prior distribution on that parameter and then form a joint normal prior over all parameters in which each parameter is independent, with one additional correction. We truncate the prior so that each parameter takes values in the stated range, so the true prior is actually a truncated multivariate normal distribution, denoted by $\pi(\boldsymbol{\theta})$. We then sample 3,171 parameter configurations from the truncated multivariate normal distribution. Let $\mathcal{S} = \{\boldsymbol{\theta}_1, \cdots, \boldsymbol{\theta}_{3171}\}$ denote the set of the sampled parameter configurations.

Simulation at Sampled Parameter Configurations To model the spread of COVID-19 among students at Cornell in the fall 2021 semester, we use a multi-group simulation to model individuals that belong to different student groups or have different vaccination status. The changes in testing processing delay and testing frequency of certain student groups as described at the beginning of SI Section 4 are also reflected in the simulation model. At each sampled parameter configuration $\boldsymbol{\theta}_i \in \mathcal{S}$, we generate 50 simulation replications and compute the corresponding 50 trajectories, each of which describes the total number of newly confirmed student cases across all student groups per day.

Calculating the Log-likelihood of the Observed Trajectory At the time of this analysis we had observed a 35-day trajectory that describes the number of daily newly confirmed infections among Cornell students and employees between 8/23/2021 and 9/25/2021 (referred to as the “observed trajectory”). We aggregate both the observed trajectory and the sampled trajectories to weekly level and estimate the log-likelihood of the observed trajectory under each parameter configuration, with details described below.

Let $y(t)$ be the total number of newly confirmed student cases across all student groups in week t. We assume that, for any week t, $y(t)$ follows a log-normal distribution and that the $y(t)$’s are conditionally independent across weeks given the simulation parameters.

Let $m(t, \boldsymbol{\theta})$ and $s(t, \boldsymbol{\theta})$ be the sample mean and sample standard deviation of the log of the count of new infections in week t across all 50 replications from the simulation under parameter configuration $\boldsymbol{\theta}$. We use these values as plug-in estimates for the hyperparameters in the log-normal distributions described above.

Then, we estimate the log-likelihood of the observed trajectory under parameter configuration θ using

$$\ell(\boldsymbol{\theta}) = \sum_{t=1}^{5} \log\left(p(y(t); m(t, \boldsymbol{\theta}), s(t, \boldsymbol{\theta}))\right),$$

where p is the density of a log-normal random variable with the given parameters.

Approximating the Posterior The log posterior density of parameter configuration $\boldsymbol{\theta}$ is given by

$$\log(\pi(\boldsymbol{\theta}|\boldsymbol{y})) = \ell(\boldsymbol{\theta}) + \log(\pi(\boldsymbol{\theta})) - \log(Z), \qquad [3]$$

where $\boldsymbol{y} = \{y(t)\}$ is the full observed trajectory, $\pi(\boldsymbol{\theta})$ is the prior for $\boldsymbol{\theta}$ and Z is the normalization constant for the posterior distribution of $\boldsymbol{\theta}$. As discussed previously, we assume that the prior for the parameters is a truncated multivariate normal distribution. We approximate it using a uniform prior across the 3171 parameter configurations sampled from the truncated normal distribution. Under this approximated prior, the posterior for $\boldsymbol{\theta}$ is also a discrete distribution over $\mathcal{S}$. To calculate the posterior probabilities, since the prior for $\boldsymbol{\theta}$ is the same for all $\boldsymbol{\theta}_i \in \mathcal{S}$, we simply compute the likelihoods $\exp(\ell(\boldsymbol{\theta}_i))$ for all $\boldsymbol{\theta}_i \in \mathcal{S}$ and normalize them to sum to one.

Based on the approximated posterior densities, we can further compute the marginal posterior distribution for individual parameters and pairs of parameters. Contour plots of the joint posterior density over pairs of parameters are given in Figure S21 (for these plots, we sample an additional 8,130 parameter configurations from the prior and use a total of 11,301 parameter configurations).

Sampling from the Posterior Distribution Equipped with the approximated posterior distribution, we are able to project the potential epidemic outcomes over the fall 2021 semester under a variety of conditions such as different testing regimes and vaccination levels. These projected outcomes may provide value for other college campuses with different situations from Cornell.

For each condition, we sample 100 parameter configurations from the posterior approximation and for each parameter configuration we simulate a single potential trajectory based on those parameters. The collection of sample trajectories represents the set of plausible outcomes over the fall 2021 semester.

We conduct two sets of simulations, one based on a set of general conditions, and one specifically targeted at Cornell:

- We consider a wide range of testing frequencies (from 0 tests per person per week to 3 tests per person per week) and vaccination level (from 25% to 100%). This analysis accommodates different college campuses with various vaccination levels and availability of testing resources.

- We specifically model the spread of COVID at Cornell using simulations that are the same as those performed on the parameter configurations sampled from the prior (see paragraph "Simulation at sampled parameter configurations") but for the full fall 2021 semester.

A Confirmatory Approach: Approximating the Posterior Using Quadratic Regression Beyond the empirical approach described above, we conduct a separate analysis to approximate the posterior distribution using a multivariate normal distribution. We use 11,301 parameter configurations sampled from the truncated multivariate normal prior in this analysis. Among the six model parameters in Table S27, we aggregate the vaccine susceptibility multiplier, vaccine transmission multiplier, and contact multiplier into one parameter called "combined spread multiplier". This is reasonable as the three parameters affect the simulation only through their product. The combined spread multiplier reflects the compound effect of vaccination and relaxation of social distancing on transmission in fall 2021. Hereafter, we treat our parameter space as four-dimensional. Let $\mathcal{S}' = \{\boldsymbol{\theta}_1, \cdots, \boldsymbol{\theta}_{11301}\}$ denote the set of the four-dimensional sampled parameter configurations.

As before, we compute the log posterior density of a parameter configuration using Equation 3. We compute $\ell(\boldsymbol{\theta}_i) + \log(\pi(\boldsymbol{\theta}_i))$ for all $\boldsymbol{\theta}_i \in \mathcal{S}'$ and ignore the constant term $\log(Z)$ as it does not have an impact on our estimation of the posterior. Here, the combined spread multiplier is the product of three variables each with a truncated normal prior. As a result, its prior does not have a closed-form expression. We compute this prior using Monte Carlo simulation. We sample 10^6 points from the prior of the vaccine susceptibility multiplier, vaccine transmission multiplier, and contact multiplier respectively, and use the empirical distribution of their product to approximate the prior distribution of the combined spread multiplier.

Let $\boldsymbol{\theta}_*$ denote the maximizer of the posterior density $\pi(\boldsymbol{\theta}|y)$. A second-order Taylor approximation of the log-posterior density around $\boldsymbol{\theta}_*$ (45) is given by

$$\log \pi(\boldsymbol{\theta}|\boldsymbol{y}) = \log \pi(\boldsymbol{\theta}_*|\boldsymbol{y}) - \frac{1}{2}(\boldsymbol{\theta} - \boldsymbol{\theta}_*)^T H_*(\boldsymbol{\theta} - \boldsymbol{\theta}_*), \qquad [4]$$

where H_* is the negative Hessian of the log-posterior at $\boldsymbol{\theta}_*$. As a result, the posterior can be interpreted as a normal distribution with mean $\boldsymbol{\theta}_*$ and covariance $\Sigma_* = (H_*)^{-1}$.

We do not, however, expect our simulated parameter configurations, which are randomly sampled from a four-dimensional space, to contain $\boldsymbol{\theta}_*$ exactly. Instead, we can use the simulated parameter configuration with the largest log-posterior value to guide the search for $\boldsymbol{\theta}_*$. Formally, let this point be denoted $\boldsymbol{\theta}'_* = \arg\max_{\boldsymbol{\theta}_i \in \mathcal{S}} \log \pi(\boldsymbol{\theta}_i|\boldsymbol{y})$. A second-order Taylor approximation of the log posterior density around $\boldsymbol{\theta}'_*$ is given by

$$\log \pi(\boldsymbol{\theta}|\boldsymbol{y}) = \log \pi(\boldsymbol{\theta}'_*|\boldsymbol{y}) + g^T(\boldsymbol{\theta} - \boldsymbol{\theta}'_*) - \frac{1}{2}(\boldsymbol{\theta} - \boldsymbol{\theta}'_*)^T H(\boldsymbol{\theta} - \boldsymbol{\theta}'_*), \qquad [5]$$

where g is the gradient of the log posterior at $\boldsymbol{\theta}'_*$ (the gradient is nonzero because $\boldsymbol{\theta}'_*$ is not the true maximizer) and H is the negative Hessian of the log posterior at $\boldsymbol{\theta}'_*$.

Given the large sample size, we assume $\boldsymbol{\theta}'_*$ is sufficiently close to $\boldsymbol{\theta}_*$ that $\boldsymbol{\theta}_*$ is the local optimum of the posterior closest to $\boldsymbol{\theta}'_*$. Then, given g and H, we can estimate $\boldsymbol{\theta}_*$ by completing the square on the right hand side of Equation 5, so that it aligns with the right hand side of Equation 4:

$$\log \pi(\boldsymbol{\theta}|\boldsymbol{y}) = \left(\log \pi(\boldsymbol{\theta}'_*|\boldsymbol{y}) + \frac{1}{2} g^T H^{-1} g\right) - \frac{1}{2}\left(\boldsymbol{\theta} - (\boldsymbol{\theta}'_* + H^{-1}g)\right)^T H \left(\boldsymbol{\theta} - (\boldsymbol{\theta}'_* + H^{-1}g)\right). \qquad [6]$$

Matching Equation 6 and Equation 4, we obtain the following estimates:

$$\hat{\boldsymbol{\theta}}_* = \boldsymbol{\theta}'_* + H^{-1}g$$
$$\log \hat{\pi}(\hat{\boldsymbol{\theta}}_*|\boldsymbol{y}) = \log \pi(\boldsymbol{\theta}'_*|\boldsymbol{y}) + \frac{1}{2} g^T H^{-1} g$$
$$\hat{\Sigma}_* = H^{-1}.$$

To find $\hat{\boldsymbol{\theta}}_*$ and $\hat{\Sigma}_*$, it suffices to find g and H. We perform a quadratic regression on the following model to estimate g and H from simulation data:

$$\log \pi(\boldsymbol{\theta}|\boldsymbol{y}) - \log \pi(\boldsymbol{\theta}'_*|\boldsymbol{y}) \sim g^T(\boldsymbol{\theta} - \boldsymbol{\theta}'_*) - \frac{1}{2}(\boldsymbol{\theta} - \boldsymbol{\theta}'_*)^T H(\boldsymbol{\theta} - \boldsymbol{\theta}'_*). \qquad [7]$$

For the regression, we select a subset of the sampled configurations that are close to $\boldsymbol{\theta}'_*$, for which the second-order Taylor approximation at $\boldsymbol{\theta}'_*$ (Equation 5) holds reasonably well. We outline the steps of performing the regression:

- For any sampled parameter configurations $\boldsymbol{\theta}_i \in \mathcal{S}'$, we compute its element-wise difference from $\boldsymbol{\theta}'_*$. Denote this difference by $\boldsymbol{d}_i = \boldsymbol{\theta}_i - \boldsymbol{\theta}'_*$.
- Next, we develop a distance metric to select points close to $\boldsymbol{\theta}'_*$. We notice that the components of $\boldsymbol{d}_i$ have vastly different scales. As a result, the L_2 norm $\|\boldsymbol{d}_i\|_2$ is dominated by a few components, which may bias the selection. Thus, we standardize each parameter component to a standard deviation of 1.
- Based on the norms of the standardized distance vectors $\{\|\tilde{\boldsymbol{d}}_i\|_2\}$, we select a fraction q of $\mathcal{S}'$ that are closest to $\boldsymbol{\theta}'_*$ to be included in the regression, denoted by $\mathcal{J}$.
- Let $K = \{1, 2, 3, 4\}$ denote the set of indices of individual parameter components. For each $\boldsymbol{\theta} \in \mathcal{J}$, we construct its features for the regression model in Equation 7, namely the linear and quadratic terms of individual parameters:

  $$\left\{\{\boldsymbol{\theta}[k]\}_{k\in K}, \{\boldsymbol{\theta}[k_1] \cdot \boldsymbol{\theta}[k_2]\}_{k_1,k_2\in K}\right\},$$

  where $\boldsymbol{\theta}[k]$ is the kth parameter component of $\boldsymbol{\theta}$. The response variable is given by $\log \pi(\boldsymbol{\theta}|\boldsymbol{y}) - \log \pi(\boldsymbol{\theta}'_*|\boldsymbol{y})$.
- Given regression results, the gradient $\hat{g}$ directly corresponds to the coefficients on the linear terms $\{\boldsymbol{\theta}[k]\}_{k\in K}$; the Hessian $\hat{H}$ can be computed from the coefficients on the quadratic terms $\{\boldsymbol{\theta}[k_1] \cdot \boldsymbol{\theta}[k_2]\}_{k_1,k_2\in K}$.

Given $\hat{g}$ and $\hat{H}$, the posterior distribution of the parameters is approximately multivariate normal with mean $\hat{\boldsymbol{\theta}}_* = \boldsymbol{\theta}'_* + (\hat{H})^{-1}\hat{g}$ and covariance $\hat{\Sigma}_* = \hat{H}^{-1}$.

Among the 11,301 sampled parameter configurations, we find $\boldsymbol{\theta}'_*$ and run the quadratic regression on $q = 1\%$ points with the smallest standardized distance to $\boldsymbol{\theta}'_*$. Table S28 shows the estimated posterior mean $\hat{\boldsymbol{\theta}}_*$ and marginal 95% credible intervals of the four parameters.

Table S28. Mean values and lower and upper bounds of marginal 95% posterior credible intervals (CI).

Parameter	Mean	Lower bound	Upper bound
Outside Infection Rate Multiplier	3.00	2.42	3.58
Contact Tracing Effectiveness Multiplier	1.13	0.96	1.30
Initial Prevalence	4.17E-3	3.86E-3	4.48E-3
Combined Spread Multiplier	0.45	0.35	0.55

The estimated posterior covariance matrix is given by

$$\hat{\Sigma}_* = \begin{bmatrix} 8.83\text{E-}2 & -1.21\text{E-}2 & -3.11\text{E-}6 & -6.77\text{E-}3 \\ -1.21\text{E-}2 & 7.61\text{E-}3 & 3.00\text{E-}7 & 3.34\text{E-}3 \\ -3.11\text{E-}6 & 3.00\text{E-}7 & 2.47\text{E-}8 & 2.44\text{E-}6 \\ -6.77\text{E-}3 & 3.34\text{E-}3 & 2.44\text{E-}6 & 2.61\text{E-}3 \end{bmatrix},$$

where parameters are in the order of outside infection rate multiplier, contact tracing effectiveness multiplier, initial prevalence, and combined spread multiplier.

To understand the interaction between different parameter components, we compute the correlation matrix. Let $\mathrm{diag}(\hat{\Sigma}_*)$ denote the diagonal matrix with ith diagonal element equal to the (i,i) entry of $\hat{\Sigma}_*$. The correlation matrix is given by

$$R = \left(\mathrm{diag}(\hat{\Sigma}_*)\right)^{-\frac{1}{2}} \hat{\Sigma}_* \left(\mathrm{diag}(\hat{\Sigma}_*)\right)^{-\frac{1}{2}} = \begin{bmatrix} 1 & -0.47 & -0.07 & -0.45 \\ -0.47 & 1 & 0.02 & 0.75 \\ -0.07 & 0.02 & 1 & 0.30 \\ -0.45 & 0.75 & 0.30 & 1 \end{bmatrix}.$$

We now compare the outcomes from the two approaches for approximating the posterior. We observe that the posterior marginal distributions from the empirical approximation (as presented in the main text) are consistent with the credible intervals from the regression-based analysis. The former observed at most weak correlation between the parameters (Fig S21), apart from the negative correlation between the constituents of the combined spread multiplier as expected. However, the latter estimated the correlations to be nontrivial between most pairs. We acknowledge an important limitation of the regression-based analysis: the correlation estimates are sensitive to the statistical fit, yet our ability to precisely estimate the derivatives of the log-likelihood is limited. It is plausible that the correlations between parameters are lower in reality.

D. Supplemental Results for Fall 2021 Projections. This section contains additional results for fall 2021 projections. Figure S22a compares simulated trajectories with the actual trajectory of infections to date. The actual trajectory appears to be tracking the lower part of the plot, but there is a high density of simulated trajectories around the actual trajectory. Figure S22b provides predictions for infections under various testing policies as a function of vaccination level. Except at high vaccination levels, the percentage of the population infected is large, even under vigorous testing.

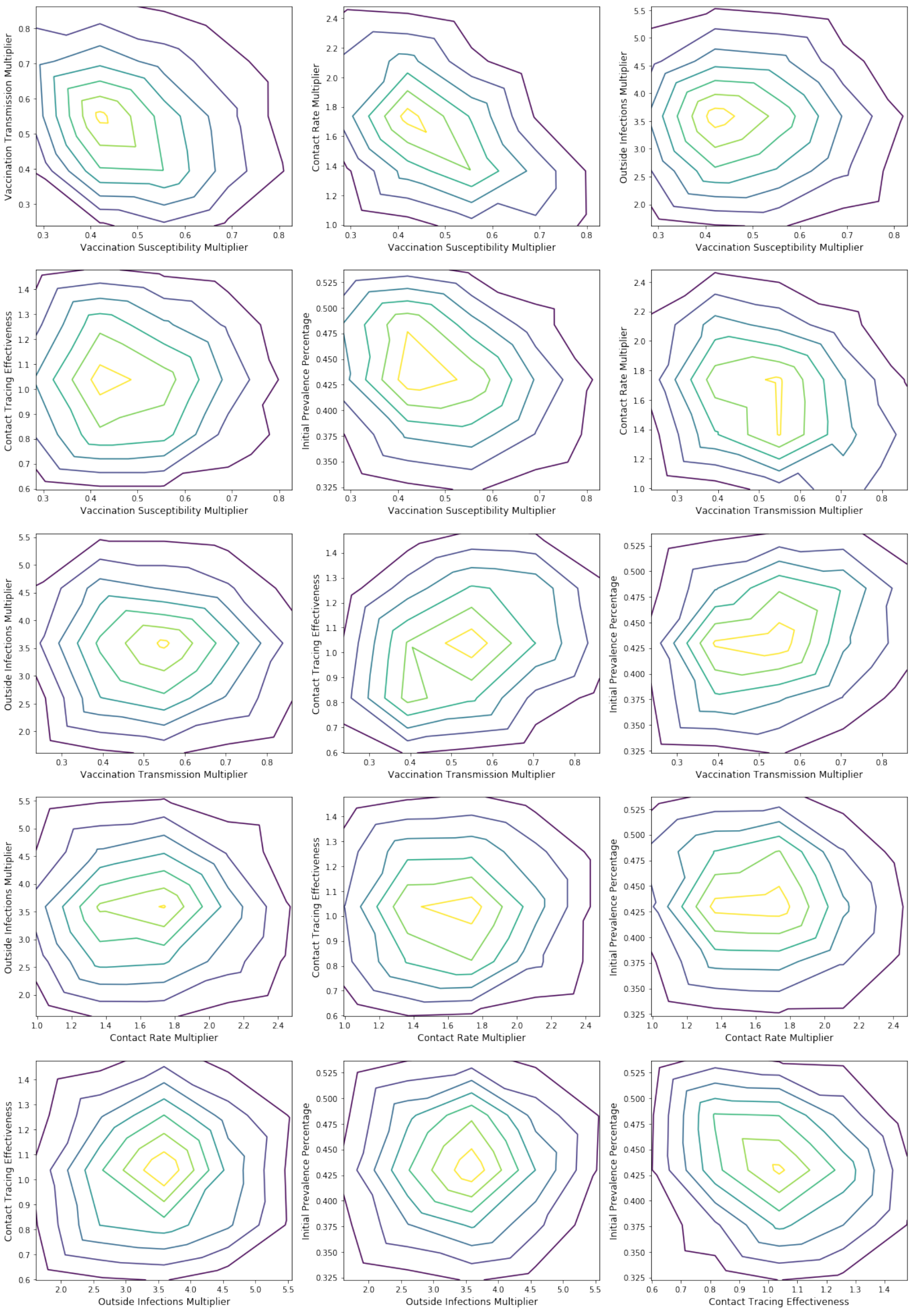

Fig. S21. Contour plots of the joint posterior density for all pairs of points for the Fall 2021 analysis.

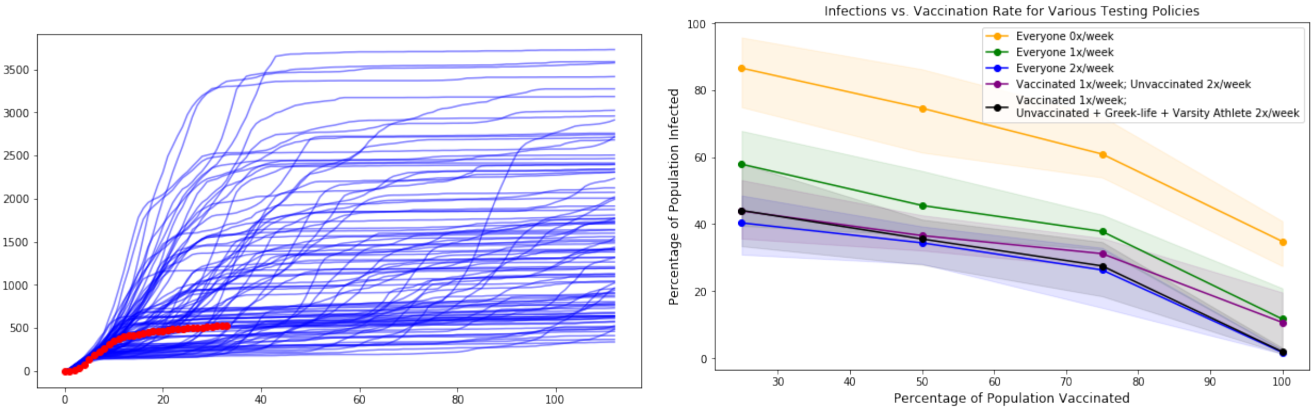

(a) **(b)**

Fig. S22. Fall 2021 modeling. (a) Fall 2021 simulated trajectories (blue) and actual trajectory (red). (b) The percentage of population infected versus vaccination rate for various testing policies (lines provide the median; shading indicates the 10-90th percentile range across simulation replications).

References

1. Cornell COVID-19 Modeling Team, COVID-19 Mathematical Modeling for Cornell's Fall Semester (https://covid.cornell.edu/_assets/files/covid_19_modeling_main_report.pdf) (2020) Accessed: 2021-10-10.
2. JM Cashore, et al., Addendum: COVID-19 Mathematical Modeling for Cornell's Fall Semester (https://covid.cornell.edu/_assets/files/covid_19_modeling_addendum.pdf) (2020) Accessed: 2021-10-10.
3. A Janmohamed, et al., Modeling Subpopulations in the Cornell Community and Greater Ithaca (https://docs.google.com/document/d/1gBoRecBRAbyONU7CgDV_rzkCVmrJJbTkSP2TpVBNl8g/edit#) (2020) Accessed: 2021-10-10.
4. Cornell COVID-19 Modeling Team, Mathematical Modeling for Cornell's Spring Semester (https://covid.cornell.edu/_assets/files/general-audience-spring-modeling-20210216.pdf) (2021) Accessed: 2021-10-10.
5. Cornell COVID Modeling Team, Modeling for Cornell's Fall 2021 Semester (https://covid.cornell.edu/_assets/files/modeling-for-cornells-fall-2021-semester.pdf) (2021) Accessed: 2021-10-10.
6. SA Lauer, et al., The incubation period of coronavirus disease 2019 (COVID-19) from publicly reported confirmed cases: estimation and application. *Annals internal medicine* **172**, 577–582 (2020).
7. L Tindale, et al., Transmission interval estimates suggest pre-symptomatic spread of COVID-19. *MedRxiv* (2020).
8. MM Arons, et al., Presymptomatic SARS-CoV-2 infections and transmission in a skilled nursing facility. *New Engl. J. Medicine* (2020).
9. WHO, Coronavirus disease 2019 (COVID-19) situation report - 73 (https://www.who.int/docs/default-source/coronaviruse/situation-reports/20200402-sitrep-73-covid-19.pdf) (2020) Accessed: 2021-10-10.
10. WHO, Report of the WHO-China joint mission on coronavirus disease 2019 (COVID-19) (https://www.who.int/docs/default-source/coronaviruse/who-china-joint-mission-on-covid-19-final-report.pdf) (2020) Accessed: 2021-10-10.
11. Centers for Disease Control and Prevention, COVID-19 pandemic planning scenarios (https://www.cdc.gov/coronavirus/2019-ncov/hcp/planning-scenarios.html) (2020) Accessed: 2021-10-10.
12. M Biggerstaff, et al., Influenza-like illness, the time to seek healthcare, and influenza antiviral receipt during the 2010–2011 influenza season—United States. *The J. infectious diseases* **210**, 535–544 (2014).
13. L Luo, et al., Modes of contact and risk of transmission in COVID-19 among close contacts. *medRxiv* (2020).
14. China CDC, The epidemiological characteristics of an outbreak of 2019 novel coronavirus diseases (COVID-19)—China, 2020. *China CDC Wkly.* **2**, 113–122 (2020).
15. Y Dong, et al., Epidemiological characteristics of 2143 pediatric patients with 2019 coronavirus disease in China. *Pediatrics* (2020).
16. CDC COVID-19 Response Team, Severe outcomes among patients with coronavirus disease 2019 (COVID-19)—united states, february 12–march 16, 2020. *MMWR Morb Mortal Wkly Rep* **69**, 343–346 (2020).
17. R Li, et al., Substantial undocumented infection facilitates the rapid dissemination of novel coronavirus (SARS-CoV-2). *Science* **368**, 489–493 (2020).
18. A Kimball, Asymptomatic and presymptomatic SARS-CoV-2 infections in residents of a long-term care skilled nursing facility—King County, Washington, March 2020. *MMWR. Morb. mortality weekly report* **69** (2020).
19. Central Intelligence Agency, The world factbook - United States (https://www.cia.gov/library/publications/the-world-factbook/geos/us.html) (2020) Accessed: 2021-10-10.
20. Kaiser Family Foundation, US population distribution by age (https://www.kff.org/other/state-indicator/distribution-by-age/) (2018) Accessed: 2021-10-10.
21. K Mizumoto, K Kagaya, A Zarebski, G Chowell, Estimating the asymptomatic proportion of coronavirus disease 2019 (COVID-19) cases on board the Diamond Princess cruise ship, Yokohama, Japan, 2020. *Eurosurveillance* **25**, 2000180 (2020).
22. R Yang, X Gui, Y Xiong, Comparison of clinical characteristics of patients with asymptomatic vs symptomatic coronavirus disease 2019 in Wuhan, China. *JAMA Netw. Open* **3**, e2010182–e2010182 (2020).
23. PopulationPyramid.net, 2019 china population (https://www.populationpyramid.net/china/2019/) (2020) Accessed: 2021-10-10.
24. Cornell Institute for Research and Planning, Cornell University factbook (http://irp.dpb.cornell.edu/university-factbook/employees) (2019) Accessed: 2021-10-10.
25. S McMullen, COVID-19 isolation and quarantine discussion with Tompkins County Health Department (2020).
26. S Mallett, et al., At what times during infection is SARS-CoV-2 detectable and no longer detectable using RT-PCR-based tests? a systematic review of individual participant data. *BMC medicine* **18**, 1–17 (2020).
27. B Giri, et al., Review of analytical performance of COVID-19 detection methods. *Anal. bioanalytical chemistry*, 1–14 (2020).
28. A Piras, et al., Inappropriate nasopharyngeal sampling for SARS-CoV-2 detection is a relevant cause of false-negative reports. *Otolaryngol. Neck Surg.* **163**, 459–461 (2020).
29. TC Jones, et al., An analysis of SARS-CoV-2 viral load by patient age. *MedRxiv* (2020).
30. J Mossong, et al., Social contacts and mixing patterns relevant to the spread of infectious diseases. *Plos Medicine* **5**, e74 (2008).
31. V Capraro, H Barcelo, The effect of messaging and gender on intentions to wear a face covering to slow down COVID-19 transmission. *arXiv preprint arXiv:2005.05467* (2020).
32. W Edmunds, G Kafatos, J Wallinga, J Mossong, Mixing patterns and the spread of close-contact infectious diseases. *Emerg. themes epidemiology* **3**, 10 (2006).

33. H Hu, K Nigmatulina, P Eckhoff, The scaling of contact rates with population density for the infectious disease models. *Math. biosciences* **244**, 125–134 (2013).
34. A Ben-Tal, A Nemirovski, Robust convex optimization. *Math. Oper. Res.* **23**, 769–805 (1998).
35. R Nunes-Vaz, C Macintyre, Observations on the current outbreak of the SARS-CoV-2 Delta variant in Sydney. *Glob. Biosecurity* **3** (2021).
36. A Puranik, et al., Comparison of two highly-effective mRNA vaccines for COVID-19 during periods of Alpha and Delta variant prevalence. *MedRxiv* (2021).
37. A Sheikh, J McMenamin, B Taylor, C Robertson, SARS-CoV-2 Delta VOC in Scotland: demographics, risk of hospital admission, and vaccine effectiveness. *The Lancet* (2021).
38. JL Bernal, et al., Effectiveness of COVID-19 vaccines against the B. 1.617. 2 variant. *medRxiv* (2021).
39. KB Pouwels, et al., Impact of Delta on viral burden and vaccine effectiveness against new SARS-CoV-2 infections in the UK. *medRxiv* (2021).
40. A Fowlkes, et al., Effectiveness of COVID-19 vaccines in preventing SARS-CoV-2 infection among frontline workers before and during B. 1.617. 2 (Delta) variant predominance—eight US locations, December 2020–August 2021. *Morb. Mortal. Wkly. Rep.* **70**, 1167 (2021).
41. M Levine-Tiefenbrun, et al., Initial report of decreased SARS-CoV-2 viral load after inoculation with the BNT162b2 vaccine. *Nat. medicine* **27**, 790–792 (2021).
42. RJ Harris, et al., Effect of vaccination on household transmission of SARS-CoV-2 in England. *New Engl. J. Medicine* (2021).
43. CM Brown, et al., Outbreak of SARS-CoV-2 infections, including COVID-19 vaccine breakthrough infections, associated with large public gatherings—Barnstable County, Massachusetts, July 2021. *Morb. Mortal. Wkly. Rep.* **70**, 1059 (2021).
44. Mike Krieger, Melanie H. Chitwood, Ted Cohen, Kenneth Gunasekera, Joshua Havumaki, Fayette Klaassen, Nicolas A. Menzies, Virginia E. Pitzer, Marcus Russi, Joshua Salomon, Nicole Swartwood, Joshua L. Warren, Daniel M. Weinberger, Ryan O'Rourke, Thomas Dimson, COVID-19 nowcasting (covidestim.org) (2021) Accessed: 2021-10-10.
45. AB Owen, *Monte Carlo theory, methods and examples.* (2013).
